# The acute effects of cannabis with and without cannabidiol in adults and adolescents: A randomised, double‐blind, placebo‐controlled, crossover experiment

**DOI:** 10.1111/add.16154

**Published:** 2023-02-26

**Authors:** Will Lawn, Katie Trinci, Claire Mokrysz, Anna Borissova, Shelan Ofori, Kat Petrilli, Michael Bloomfield, Zarah R. Haniff, Daniel Hall, Natalia Fernandez‐Vinson, Simiao Wang, Amir Englund, Edward Chesney, Matthew B. Wall, Tom P. Freeman, H. Valerie Curran

**Affiliations:** ^1^ Department of Psychology, Institute of Psychiatry Psychology and Neuroscience King's College London London UK; ^2^ Department of Addictions, Institute of Psychiatry Psychology and Neuroscience King's College London London UK; ^3^ Clinical Psychopharmacology Unit, Clinical Educational and Health Psychology University College London London UK; ^4^ Department of Neuroimaging, Institute of Psychiatry Psychology and Neuroscience King's College London London UK; ^5^ NIHR University College London Hospitals Biomedical Research Centre University College Hospital London UK; ^6^ Addiction and Mental Health Group (AIM), Department of Psychology University of Bath Bath UK; ^7^ Translational Psychiatry Research Group, Research Department of Mental Health Neuroscience, Division of Psychiatry University College London London UK; ^8^ Department of Psychosis Studies, Institute of Psychiatry Psychology and Neuroscience King's College London London UK; ^9^ Invicro London Burlington Danes Building, Hammersmith Hospital, Du Cane Road London UK

**Keywords:** adolescence, cannabis, CBD, cognition, memory, psychosis, subjective drug effects, THC

## Abstract

**Background and Aims:**

Long‐term harms of cannabis may be exacerbated in adolescence, but little is known about the acute effects of cannabis in adolescents. We aimed to (i) compare the acute effects of cannabis in adolescent and adult cannabis users and (ii) determine if cannabidiol (CBD) acutely modulates the effects of delta‐9‐tetrahydocannabinol (THC).

**Design:**

Randomised, double‐blind, placebo‐controlled, crossover experiment. The experiment was registered on ClinicalTrials.gov (NCT04851392).

**Setting:**

Laboratory in London, United Kingdom.

**Participants:**

Twenty‐four adolescents (12 women, 16‐ to 17‐year‐olds) and 24 adults (12 women, 26‐ to 29‐year‐olds) who used cannabis 0.5–3 days/week and were matched on cannabis use frequency (mean = 1.5 days/week).

**Intervention:**

We administered three weight‐adjusted vaporised cannabis flower preparations: ‘THC’ (8 mg THC for 75 kg person); ‘THC + CBD’ (8 mg THC and 24 mg CBD for 75 kg person); and ‘PLA’ (matched placebo).

**Measurements:**

Primary outcomes were (i) subjective ‘feel drug effect’; (ii) verbal episodic memory (delayed prose recall); and (iii) psychotomimetic effect (Psychotomimetic States Inventory).

**Findings:**

Compared with ‘PLA’, ‘THC’ and ‘THC + CBD’ significantly (*P* < 0.001) increased ‘feel drug effect’ (mean difference [MD] = 6.3, 95% CI = 5.3–7.2; MD = 6.8, 95% CI = 6.0–7.7), impaired verbal episodic memory (MD = –2.7, 95% CI = −4.1 to −1.4; MD = −2.9, 95% CI = −4.1 to −1.7) and increased psychotomimetic effects (MD = 7.8, 95% CI = 2.8–12.7; MD = 10.8, 95% CI = 6.2–15.4). There was no evidence that adolescents differed from adults in their responses to cannabis (interaction *P* ≥ 0.4). Bayesian analyses supported equivalent effects of cannabis in adolescents and adults (Bayes factor [BF_01_] >3). There was no evidence that CBD significantly modulated the acute effects of THC.

**Conclusions:**

Adolescent cannabis users are neither more resilient nor more vulnerable than adult cannabis users to the acute psychotomimetic, verbal memory‐impairing or subjective effects of cannabis. Furthermore, in adolescents and adults, vaporised cannabidiol does not mitigate the acute harms caused by delta‐9‐tetrahydocannabinol.

## INTRODUCTION

Cannabis is the most commonly used internationally controlled drug in the world and is particularly popular among adolescents. Between 15% and 20% of English 15‐year‐olds [1, 2] and 28% of American 15‐ to 16‐year‐olds [3] report using cannabis in the last year. Globally, roughly half of those who try cannabis do so are aged 18.5 years or younger [4]. Adolescents are approximately three times more likely than adults to develop cannabis addiction in the years immediately after initiating cannabis use [5, 6, 7]. Meanwhile, cannabis laws are rapidly evolving across the world [8], which is likely to have profound impacts on young people [9, 10, 11]. Accordingly, there is an urgent need to understand the acute psychopharmacological effects of cannabis in teenagers.

Adolescence is a period during which important biological, neural, psychological, social and personal changes occur [12, 13, 14, 15]. Crucially, the endocannabinoid (eCB) system continues to develop, with changing levels of cannabinoid receptors and endocannabinoids [16, 17]. It is also a time when mental health problems typically begin to emerge [18], with 14 years being the average age of onset of mental illness [19]. In epidemiological studies, long‐term cannabis use in adolescence has been associated with an increased likelihood of psychosis [20], mood disorders [21] and poorer cognitive function and educational achievement [22]. Therefore, there is concern that the potential harms of cannabis use may be accentuated in adolescents who are at a critical neurodevelopmental stage [23]. However, the acute effects of cannabis intoxication in adolescents have rarely been studied in controlled settings.

Acutely, cannabis reliably produces an array of transient and dose‐dependent effects including intoxication, euphoria, memory impairment, psychotomimetic effects, anxiogenic effects, attention deficits and tachycardia [24, 25, 26, 27]. Notably, there is wide interindividual variation [27]. The major psychoactive effects of cannabis arise from delta‐9‐tetrahydrocannabinol (THC), which is a partial cannabinoid‐1 receptor (CB1R) agonist. Cannabidiol (CBD), which is the second most abundant phytocannabinoid [28], has a complex pharmacology [29], including inhibition of anandamide metabolism [27], agonism of the serotonin‐1A receptor [30] and negative allosteric modulation of CB1 receptors [31]. CBD may moderate some of the acute effects of THC [32]; however, findings have been inconsistent. CBD has sometimes been shown to attenuate the short‐term harmful effects of THC [33] and sometimes not [34], which may be related to CBD dose, route of administration and user characteristics. High‐dose oral (600 mg) and intravenous (5 mg) CBD attenuated the psychotomimetic effects [35, 36] and episodic memory impairing effects of THC [36], but 8 mg of vaporised CBD did not [34, 37]. The moderating influence of CBD co‐administration on the acute effects of cannabis in adolescents is unknown.

Despite the pervasiveness of adolescent cannabis use, only one controlled human experiment has compared the acute effects of cannabis in adolescents under the age of 18 years and adults [38]. Participants were 20 adolescent males (16–17 years old) and 20 adult males (24–28 years old) who used cannabis an average of 2.4 and 1.8 days/week, respectively. Participants inhaled vaporised cannabis (8 mg THC for a 75 kg person) or placebo cannabis. Notably, adolescents reported less intense subjective effects, fewer psychotomimetic symptoms and exhibited less episodic memory impairment than adults. However, in this study, adolescents used cannabis significantly more frequently than adults and weighed less than adults, so received a lower absolute dose of THC.

More recently, one placebo‐controlled study compared the impact of oral THC (7.5 and 15 mg) in young people 18–20 years old and adults 30–40 years old with limited cannabis experience [39]. They found similar responses on all subjective effects, cardiovascular measures, working memory and response inhibition; however, the 18‐ to 20‐year‐olds experienced stronger electroencephalography and psychomotor changes compared with adults [39]. One observational, ecological momentary assessment study examined the associations between age and subjective response in 15‐ to 24‐year‐old frequent cannabis users [40]. Contrastingly, they reported that being older was associated with diminished self‐reported stimulatory response and subjective ‘high’. Moreover, non‐human animal research has found mixed results; acute cannabinoid agonists elicited stronger anxiogenic effects in adults [41], but provoked greater impairments in learning in adolescents [42].

In sum, no studies have investigated the combined effects of CBD and THC in adolescents, in whom the impact may be different from adults. Only one controlled experiment to date has compared the acute effects of cannabis in human adolescents under the age of 18 years with adults [38], which had the following limitations: (i) no female participants; (ii) the adolescents used cannabis more frequently than the adults; (iii) the modulatory impact of CBD was not studied; and (iv) no pharmacokinetic data were collected.

To address these limitations, we tested equal numbers of female and male participants, included a combined THC and CBD (‘THC + CBD’) condition as well as THC‐only (‘THC’) and placebo (‘PLA’) conditions, carefully matched cannabis use frequency between the groups and collected pharmacokinetic data. In the largest study of its kind, we aimed to replicate the adolescent versus adult difference in acute effects of cannabis and to explore the modulatory impact of CBD co‐administration in these age groups.

First, we hypothesised that, across age‐group, there would be a main effect of drug, such that (i) subjective ‘feel drug effect’, (ii) verbal episodic memory impairment, and (iii) psychotomimetic effects, would be greater on ‘THC’ and ‘THC + CBD’ than placebo. Second, we hypothesised that CBD in ‘THC + CBD’ would reduce the (i) verbal episodic memory impairing, and (ii) psychotomimetic effects, relative to ‘THC’, but (iii) subjective ‘feel drug effect’ would be similar on ‘THC’ and ‘THC + CBD’. Third, we hypothesised there would be an interaction between age‐group and drug, such that the expected difference between the ‘THC’ and ‘PLA’ conditions on measures (i)–(iii) would be greater for adults than for adolescents.

## METHODS

### Study design and participants

The experiment was registered on ClinicalTrials.gov (NCT04851392), and the protocol was uploaded to the Open Science Framework (OSF) [43].

We conducted a randomised, double‐blind, placebo‐controlled, crossover experiment on the acute effects of cannabis in adolescents and adults. The experiment had a between‐subjects factor of age group (adolescent and adult) and a within‐subjects factor of drug (‘THC’, ‘THC + CBD’, ‘PLA’).

This was a single‐centre study conducted at University College London and Invicro (Hammersmith Hospital, United Kingdom [UK]). We recruited healthy participants from the community in London, UK. The study received full ethical approval from UCL Ethics Committee (project code: 5929/005). This study was registered on Clinical.Trials.gov (NCT04851392). All participants provided written, informed consent and provided verbal ongoing consent at the beginning of each study session.

We continued recruitment until we reached our target sample size of 48 completed participants, comprising 24 adolescents (50% female; 16–17 years old) and 24 adults (50% female; 26–29 years old). Participants who did not complete all sessions were replaced. Key inclusion criteria were self‐reported use of cannabis 0.5, 1, 2 or 3 days/week; healthy or overweight body mass index (BMI); and right‐handed. Key exclusion criteria were The Diagnostic and Statistical Manual of Mental Disorders fifth edition (DSM‐5) severe cannabis use disorder; currently receiving treatment for a mental health disorder; history of personal or familial psychosis; pregnant or breast‐feeding; use of any illicit drug (apart from cannabis) more than 2 days per month; nicotine dependence as defined by a Heaviness of Smoking score >1 [44]; currently taking a psychotropic medication that interacts with cannabis or directly affects outcome variables; magnetic resonance imaging (MRI) contraindications; and if an adult, cannabis use at a frequency ≥1 day/week before turning 18 years old. For full eligibility criteria, see Table S1. Cannabis use frequency (days per week) was purposefully matched between the adolescents and adults.

### Randomisation and blinding

There were six drug orders. Each order was used with eight participants (one sixth of total sample), to ensure that drug order was balanced. Drug order was balanced within age‐group and gender. Within these groups, participants were randomly allocated to drug order using a blocked randomisation sequence. All experimental researchers and participants were blinded to treatment allocation. At the end of each session, participants were asked to guess which drug they thought they had received that day.

### Procedures

Participants were screened online and via telephone. Potentially eligible participants attended an in‐person (pre‐coronavirus disease [COVID]) or virtual (post‐COVID) baseline session. In‐person eligibility criteria were confirmed at either the in‐person baseline or first drug administration session.

Eligible participants then completed three drug administration sessions. At the start of each session, recent alcohol and illicit drug use were checked to ensure abstinence via an alcohol breathalyser, a saliva drugs test and self‐report. Female participants completed a urine pregnancy test. Drug administration sessions lasted 5–6 h in total. Participants completed assessments before and after cannabis inhalation (Table 1 and Figure S1). MRI data, other neurocognitive task data and detailed pharmacokinetic and physiological data will be reported elsewhere, as these results are beyond the scope of this manuscript.

**TABLE 1:** Schedule of experiment events for drug administration sessions to the nearest 5 min, relative to the start of drug administration (T‐inh)

Time (min)	Timepoint	Event
−70		In‐person eligibility checks: alcohol breathalyser, saliva drugs test, pregnancy test, recent drug use, TLFB, physical checks, BMI
−40		Cannulation, radiographer checks
−5	T1	Blood sample, BP/HR, VAS
0	T‐inh	Drug administration
+20	T2	Blood sample, BP/HR, VAS
+25		Prose recall (immediate)
+30	T3	Blood sample, BP/HR, VAS
+45		MRI scan
+120	T4	BP/HR, subjective effects
+125		Prose recall (delayed)
+125		Psychotomimetic states inventory
+140		Neurocognitive tasks
+160	T5	Blood sample, BP/HR, VAS
+180		PANSS interview
+210		Drug guess, subjective ratings, sobriety tests, discharge

*Note*: MRI data, neurocognitive task data and pharmacokinetic and physiological data at T3, T4 and T5 will be reported elsewhere.

Abbreviations: BMI = body mass index; BP = blood pressure; HR = heart rate; MRI = magnetic resonance imaging; PANSS = Positive and Negative Syndrome Scale; PSI = Psychotomimetic States Inventory; T‐inh = the time that drug inhalation started; TLFB = timeline follow‐back; VAS = visual analogue scale.

Dried medical cannabis flower was procured from Bedrocan (https://bedrocan.com/) in the Netherlands and imported under H.V.C.'s UK Home Office licence. Cannabis types used were ‘Bedrocan’ (20.2% THC; 0.1% CBD), ‘Bedrolite’ (0.4% THC; 8.5% CBD) and ‘Bedrobinol Placebo’. Dose was weight‐adjusted [38]: The ‘THC’ condition contained 0.107 mg/kg THC (8 mg THC for a 75 kg person); the ‘THC + CBD’ condition contained 0.107 mg/kg THC and 0.320 mg/kg CBD (8 mg THC and 24 mg CBD for a 75 kg person); and the ‘PLA’ condition contained no THC or CBD and was matched in appearance and smell to the other conditions. This dose of THC corresponds to approximately one quarter of a typical ‘joint’ [45, 46], or 1.6 standard THC units [47, 48]. The total weight of cannabis flower material was kept constant across the conditions through the addition of placebo cannabis. Cannabis was vaporised into a ‘balloon’ using a Volcano Medic Vaporiser (Storz and Bickel), and participants inhaled the contents of the balloon according to a standardised, timed procedure. The minimum washout period was 3 days (72 h) between drug sessions [49]; the elimination half‐life of THC is 21.5 h [50, 51], giving adequate time for THC elimination.

### Outcomes

The registered primary outcome variables were (i) strength of the subjective drug effect, as measured by self‐reported ‘feel drug effect’ [38] 20 min after the start of cannabis inhalation (i.e. almost immediately after inhalation completion) (T2); (ii) verbal episodic memory, as measured by delayed prose recall [52] 2 h after the start of cannabis inhalation (T4); and (iii) psychotomimetic effect, as measured by the total Psychotomimetic States Inventory (PSI) score [53] 2 h after the start of cannabis inhalation (T4).

The subjective ‘feel drug effect’ visual analogue scale (VAS) is a single‐item scale rated from 0 (not at all) to 10 (extremely). The prose recall task requires participants to listen to a 30‐s story presented via headphones, immediately write down as much as they can remember, and then again write down as much as they can remember 100 min later. The PSI is a 48‐item self‐report questionnaire assessing psychotic‐like symptoms.

Secondary variables were (i) psychotomimetic effect, as measured by the Positive and Negative Syndrome Scale (PANSS) positive and negative subscales [54] 3 h after the start of cannabis inhalation, when the participant is sober and reflects on their experiences; (ii) psychotomimetic effect measured via PSI subscales, including delusory thinking, perceptual distortion, cognitive disorganisation, anhedonia, mania and paranoia, 2 h after the start of drug inhalation (T4); (iii) subjective effects of ‘anxious’, ‘paranoid’, ‘want cannabis’, ‘feel drug effect’, ‘like drug effect’, ‘dislike drug effect’ and ‘happy’ measured via 10‐point visual analogue scales (VAS; 0 not at all to 10 extremely) at 5 min pre‐cannabis (T1), 20 min (T2), 30 min (T3), 120 min (T4) and 160 min (T5) after the start of cannabis inhalation; (iv) THC and CBD plasma levels 20 min after the start of cannabis inhalation (T2); and (v) heart rate 20 min after the start of cannabis inhalation (T2). See Supporting Information for a detailed description of all measures.

### Statistical analyses

The study was powered to replicate our previous finding that adolescents experienced a weaker psychotomimetic effect of cannabis than adults [38]. We found an effect size of Cohen's f = 0.33 for the age‐group (adolescent, adult) by drug (THC, placebo) interaction on total PSI score. To obtain 80% power in our current experiment to replicate this 2 × 2 interactive result, with ɑ = 0.05, number of groups = 2, number of repeated measures = 2 (‘THC’ and ‘PLA’) and a conservative repeated‐measures correlation = 0.2, 32 participants were required. We, therefore, recruited 48 participants to complete the study and account for winner's curse (+50%) [55]. For other analyses, with our set *n* = 48, three repeated measures, ɑ = 0.05, we had an estimated 80% power to detect a within‐subjects main effect (drug) and a within‐between interaction (age group by drug) with effect sizes of Cohen's f ≥0.24.

The analysis followed our OSF analysis plan [56], which was published before data collection was completed and before data were unblinded. A per‐protocol analysis was used; only participants who completed all three experimental sessions were included. Data were first pre‐processed, and assumptions were examined (see Supporting Information). For each of the three primary outcome variables, a 2 × 3 mixed ANOVA with a between‐subjects factor of age group (adolescent, adult) and a within‐subjects factor of drug (‘PLA’, ‘THC’, ‘THC + CBD’) was conducted. The main effect of drug (‘PLA’ vs ‘THC’ vs ‘THC + CBD’) and the interaction between drug and age group were first examined. Where significant main effects or interactions were detected (α cut‐off of 0.05), these were explored with *post hoc* pairwise Bonferroni‐corrected *t* tests, which compared all conditions against one another. We corrected for multiple comparisons within each primary outcome variable, but not across all primary outcome variables. If a Greenhouse–Geisser correction was implemented, we report uncorrected degrees of freedom. Where data violated assumptions for parametric analyses, we proceeded with parametric tests, but non‐parametric analyses were also conducted to support findings. We re‐ran all primary analyses with drug order included as a between‐subjects covariate to test whether drug order altered the pattern of results.

*Post hoc* Bayesian *t* tests were conducted to support null differences. Bayes factors (BF_01_) >3 were interpreted as evidence in favour of no difference. For primary outcome variables, differences between ‘THC’ and ‘PLA’ (‘THC minus PLA’) were calculated and underwent Bayesian independent *t* tests to test for equivalence in response to cannabis in adolescents and adults. Across age‐group, Bayesian paired *t* tests were used to test for equivalence in ‘THC + CBD’ and ‘THC’ conditions. All analyses were conducted using SPSS (Version 25).

Secondary outcome variables were primarily analysed via two‐way or three‐way mixed ANOVAs similar to the ANOVAs described above. Three‐way ANOVAs included a within‐subject factor of time. For subjective effect VASs, time had levels: T1/T2 to T5, and separately area‐under‐the‐curve and peak effects were analysed. For prose recall, time had levels: immediate and delayed. For THC and CBD plasma results at T2, mixed effects models were performed to account for eight missing data points out of 144. Positive and negative PANSS scores were analysed using mixed ANOVAs and supported with generalised estimating equations models and χ^2^ and McNemar's tests. See Supporting Information for full details.

## RESULTS

Data collection started on 11 March 2019 and concluded on 16 June 2021 when 48 participants (*n* = 24 adolescents, 50% female and *n* = 24 adults, 50% female) had completed all three drug administration sessions, as planned (Consolidated Standards of Reporting Trials diagram; Figure S3). Data collection was suspended from March 2020 until November 2020 because of the COVID‐19 pandemic, after which the experiment resumed in a safe way according to government and local regulations.

After weight adjustment by participants' actual weight, adolescents received a mean THC dose of 6.8 mg (SD = 0.9; range = 5.4–8.7 mg), and adults received a mean THC dose of 7.4 mg (SD = 1.4; range = 5.4–10.7 mg) on both ‘THC’ and ‘THC + CBD’ conditions. On ‘THC + CBD’ adolescents received a mean CBD dose of 20.3 mg (SD = 2.6; range = 16.3–26.1 mg), and adults received a mean CBD dose of 22.2 mg (SD = 4.3; range = 16.3–32.2 mg) of CBD. These differences were not statistically significant (THC: *t*(45) = 1.760, *P* = 0.085; CBD: *t*(45) = 1.762, *P* = 0.085).

For a summary of participant characteristics, see Table 2 (for full details see Tables S4–S8). Adolescents had a mean age of 17.17 years (SD = 0.43), and adults had a mean age of 27.77 years (SD = 1.04). Cannabis use frequency was purposefully matched between adolescents (1.41 days/week, SD = 0.77) and adults (1.45 days/week, SD = 0.77). Adolescents and adults had similar socio‐economic status, ethnicity, problematic alcohol consumption levels, cigarette/roll‐up use and other illicit drug use. However, adolescents had higher depression, anxiety and impulsiveness scores and adults had higher verbal IQ and alcohol use frequency.

**TABLE 2:** Summary of participants' baseline sociodemographic characteristics and drug use

	Adolescents (*n* = 24)	Adults (*n* = 24)	Both (*n* = 48)
Gender			
Female	12 (50.0%)	12 (50.0%)	24 (50.0%)
Male	12 (50.0%)	12 (50.0%)	24 (50.0%)
Age (years)*	17.17 (0.43) [16.50–17.92]	27.77 (1.04) [26.33–29.58]	22.47 (5.41) [16.50–29.58]
BMI (kg/m^2^)	21.89 (2.81) [17.36–26.67]	23.18 (2.73) [19.40–27.80]	22.53 (2.82) [17.36–27.80]
Ethnicity			
White	17 (70.8%)	18 (75.0%)	35 (72.9%)
Mixed	4 (16.7%)	1 (4.2%)	5 (10.4%)
Asian	1 (4.2%)	3 (12.5%)	4 (8.3%)
Black	0 (0.0%)	2 (8.3%)	2 (4.2%)
Other	1 (4.2%)	0 (0.0%)	1 (2.1%)
Prefer not to say	1 (4.2%)	0 (0.0%)	1 (2.1%)
SES			
Mother's education below undergraduate degree	8 (33.3%)	8 (33.3%)	16 (33.3%)
Mother's education undergraduate degree or above	16 (66.7%)	16 (66.7%)	32 (66.7%)
BDI*	10.38 (8.55) [0.00–28.00]	5.29 (6.45) [0.00–22.00]	7.83 (7.92) [0.00–28.00]
s‐UPPS‐P*	48.17 (7.51) [34.00–61.00]	42.75 (8.87) [30.00–64.00]	45.46 (8.58) [30.00–64.00]
CUDIT‐R*	10.17 (3.14) [5.00–16.00]	7.21 (3.31) [3.00–15.00]	8.69 (3.53) [3.00–16.00]
AUDIT*	5.88 (5.39) [0.00–21.00]	7.71 (4.59) [1.00–18.00]	6.79 (5.04) [0.00–21.00]
Cannabis use frequency (days/week) measured at baseline	1.41 (0.77) [0.25–3.50]	1.45 (0.77) [0.25–2.75]	1.43 (0.76) [0.25–3.50]
Cannabis use frequency (days/week) measured at screening			
0.5	4 (16.7%)	4 (16.7%)	8 (16.7%)
1	7 (29.2%)	5 (20.8%)	12 (25.0%)
2	8 (33.3%)	7 (29.2%)	15 (31.3%)
3	5 (20.8%)	8 (33.3%)	13 (27.1%)
Estimated amount of cannabis used on day of use (grams)	0.81 (0.56) [0.25–2.50]	0.50 (0.52) [0.10–2.00] {n = 23}	0.66 (0.55) [0.10–2.50] {n = 47}
Age of first cannabis use (years)*	14.55 (1.03) [11.92–16.08]	18.17 (2.62) [14.00–24.42]	16.36 (2.69) [11.92–24.42]
Lifetime days of cannabis use*	153.67 (89.97) [11.00–418.00]	544.29 (630.94) [136.00–3120.00]	349.13 (487.63) [11.00–3127.00]
Weekly alcohol use*			
No	19 (79.2%)	6 (25.0%)	25 (52.1%)
Yes	5 (20.8%)	18 (75.0%)	23 (47.9%)
Weekly cigarette roll‐up use			
No	15 (62.5%)	19 (79.2%)	34 (70.8%)
Yes	9 (37.5%)	5 (20.8%)	14 (29.2%)
Monthly use of any other illicit drug			
No	22 (91.7%)	22 (91.7%)	44 (91.7%)
Yes	2 (8.3%)	2 (8.3%)	4 (8.3%)

*Note*: For continuous data, mean (SD) [minimum–maximum] is shown, for categorical data, *n* (%) is shown.

Abbreviations = AUDIT, Alcohol Use Disorders Identification Test; BDI = Beck Depression Inventory; CUDIT‐R = Cannabis Use disorder Identification Test‐Revised; SES = socio‐economic status; s‐UPPS‐P = Short Urgency‐Premeditation‐Perseverance‐Sensation Seeking‐Positive Urgency.

* *P* < 0.05.

Pre‐drug administration (T1) minimal THC was detected in plasma. Across the three conditions, four participants had a plasma THC level >0.5 ng/mL; all other participants had plasma THC levels that were zero. The non‐zero levels were all <1.25 ng/mL. Pre‐drug administration, at T1, across the three conditions, all participants had a zero plasma CBD level (Table S17a).

### Primary outcomes

**FIGURE 1:**
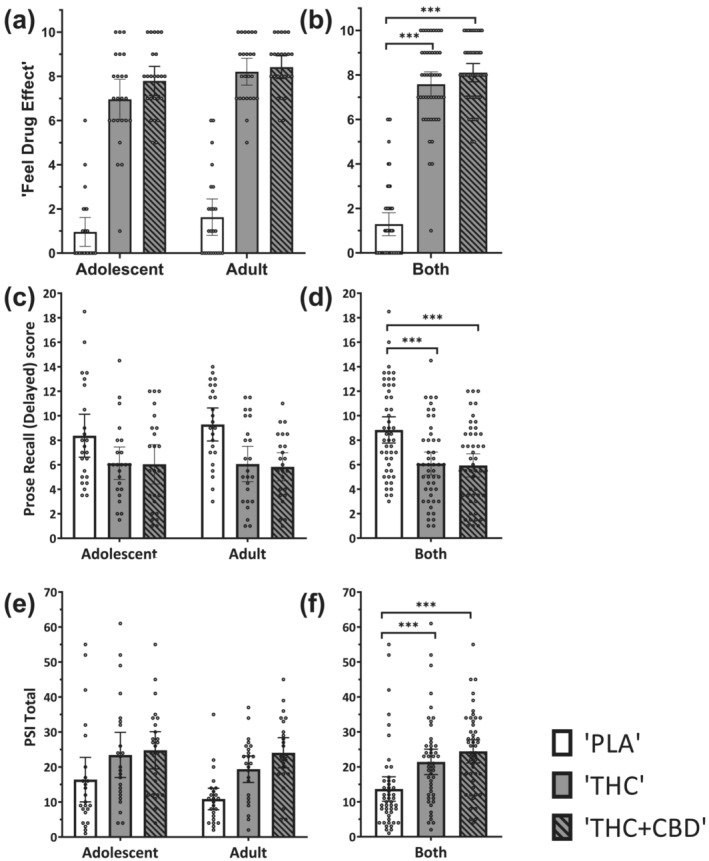
Mean values with data points overlaid and 95% CI displayed for primary outcomes. (a) VAS ‘feel drug effect’ at T2, (c) prose recall score (delayed) at T4 and (e) PSI total at T4, for adolescents (*n* = 24) and adults (*n* = 24) during ‘THC’, ‘THC + CBD’ and ‘PLA’ conditions. Main effect of drug across age groups displayed in (b), (d), and (f) where their data are combined (*n* = 48). **P < 0.01, ***P < 0.001. PSI = psychotomimetic states inventory; VAS = visual analogue scale; THC = delta‐9‐tetrahydocannabinol; CBD = cannabidiol; PLA = placebo.

For subjective ‘feel drug effect’ (at T2, 20 min; Table S9; Figure 1a,b), a Greenhouse–Geisser correction was implemented. There was no significant interaction between drug and age‐group (*F*[2,92] = 0.557; *P* = 0.547; η^2^_p_ = 0.012). There was a main effect of both drug (*F*[2,92] = 262.214; *P* < 0.001; η^2^_p_ = 0.851) and age group (*F*[1,45] = 8.190; *P* = 0.006; η^2^_p_ = 0.151). ‘THC’ led to greater ratings than ‘PLA’ (mean difference [MD] = 6.292; 95% CI = 5.343–7.240; *t*(46) = 16.480; *P* < 0.001), and ‘THC + CBD’ led to greater ratings than ‘PLA’ (MD = 6.813; 95% CI = 5.964–7.661; *t*(46) = 19.957; *P* < 0.001); there was no significant difference between ‘THC’ and ‘THC + CBD’ (MD = 0.521; 95% CI = −0.121 to 1.163; *t*(46) =2.017; *P* = 0.149). Overall, across the three drug conditions (including ‘PLA’), adults had larger ‘feel drug effect’ ratings than adolescents (MD = 0.847; 95% CI = 0.251–1.443; *t*(45) =2.862; *P* = 0.006). However, null age‐group differences in the ‘THC minus PLA’ value were supported by Bayesian analysis (BF_01_ = 3.591), providing evidence that adolescents and adults responded similarly. When comparing ‘THC’ and ‘THC + CBD’, Bayesian results were inconclusive (BF_01_ = 1.328). These results did not significantly change when controlling for drug order or when removing participants with outlying subjective or plasma data.

For prose recall delayed scores (Table S9 and Figure 1c,d), there was no significant interaction between drug and age group (*F*[2,92] = 0.727; *P* = 0.486; η^2^_p_ = 0.016). There was a main effect of drug (*F*[2,92] = 20.616; *P* < 0.001; η^2^_p_ = 0.309), but not of age group (*F*[1,45] = 0.073; *P* = 0.789; η^2^_p_ = 0.002). ‘THC’ led to lower (delayed) prose recall scores than ‘PLA’ (MD = −2.740; 95% CI = −4.059 to −1.420; *t*(46) = 5.158; *P* < 0.001). ‘THC + CBD’ also led to lower scores than ‘PLA’ (MD = −2.896; 95% CI = −4.130 to −1.662; *t*(46) = 5.830; *P* < 0.001). Furthermore, there was no significant difference between scores during ‘THC’ and ‘THC + CBD’ (MD = 0.156; 95% CI = −1.069 to 1.381; *t*(46) = 0.317; *P* = 1.00). Null age‐group differences in ‘THC minus PLA’ were supported by Bayesian analysis (BF_01_ = 3.192), providing evidence that adolescents and adults responded similarly. Additionally, Bayesian analysis provided evidence that CBD did not impact THC effects on prose recall (BF_01_ = 8.423). These results did not significantly change when controlling for drug order or when removing participants with outlying subjective or plasma data.

For psychotomimetic effects (PSI total score; Table S9, Figure 1e,f), there was no significant interaction between drug and age group (*F*[2,92] = 0.932; *P* = 0.398; η^2^_p_ = 0.020). There was a main effect of drug (*F*[2,92] = 18.796; *P* < 0.001; η^2^_p_ = 0.290), but not of age‐group (*F*[1,45] = 1.582; *P* = 0.215; η^2^_p_ = 0.033). Across age group, ‘THC’ led to greater PSI ratings than ‘PLA’ (MD = 7.771; 95% CI = 2.844–12.698; *t*(46) = 3.919; *P* = 0.001), and ‘THC + CBD’ led to greater ratings than ‘PLA’ (MD = 10.792; 95% CI = 6.172–15.411; *t*(46) = 5.805; *P* < 0.001); there was no significant difference between ‘THC’ and ‘THC + CBD’ (MD = –3.021; 95% CI = −6.954 to 0.912; *t*(46) =1.908; *P* = 0.188). Null age‐group differences for PSI ratings in ‘THC minus PLA’ were supported by Bayesian analysis (BF_01_ = 4.385), providing evidence that adolescents and adults responded similarly. When comparing ‘THC’ and ‘THC + CBD’, Bayesian results were inconclusive (BF_01_ = 1.589). These results did not significantly change when controlling for drug order or when removing participants with outlying subjective or plasma results.

### Secondary outcomes

Across secondary outcomes, the results generally followed a similar pattern (see Table 3 for summary) such that there were typically no drug by age‐group interactions and there were main effects of drug. Post hoc pairwise drug comparisons generally showed significant differences between ‘PLA’ compared with ‘THC’ and ‘THC + CBD’ and occasionally differences between outcomes during ‘THC’ and ‘THC + CBD’.

**TABLE 3:** Summary of key results for primary and secondary outcome variables

Outcome	Drug*age‐group interaction	Main effect of drug	‘THC’ > ‘PLA’	‘THC + CBD’ > ‘THC’
Primary				
Feel drug effect (T2)	–	Y	Y	–
Prose recall delayed	–	Y	Y	–
PSI total	–	Y	Y	–
Secondary				
Subjective measures (VAS)				
Feel drug effect (T2–T5)	–	Y	Y	Y
Like drug effect (T2–T5)	–	Y	Y	–
Dislike drug effect (T2–T5)	–	–	–	–
Anxious (T1–T5)	–	Y	Y	–
Paranoid (T1–T5)	–	Y	Y	–
Want cannabis (T1–T5)	–	–	–	–
Happy (T1–T5)	Y^a^	–	–	–
Prose recall				
Prose recall immediate	–	Y	Y	–
PSI subscales				
Delusory thinking	–	–	–	–
Perceptual distortion	–	Y	Y	‐
Cognitive disorganisation	–	Y	Y	Y
Anhedonia	–	–	–	–
Mania	–	Y	–	–
Paranoia	–	Y	–	–
PANSS subscales				
PANSS positive	–	Y	Y	–
PANSS negative	–	Y	Y	–
Plasma and heart rate				
THC plasma concentration (T2)	–	Y	Y	Y
CBD plasma concentration (T2)	Y^b^	Y	–	Y
Heart rate (T2)	–	Y	Y	Y

*Note*: Significant results (*P* < 0.05) are denoted ‘Y’ and non‐significant results are denoted ‘**–**’. Abbreviations: CBD = cannabidiol; PANSS = Positive and Negative Syndrome Scale; PLA = placebo; PSI = Psychotomimetic States Inventory; THC = delta‐9‐tetrahydocannabinol; VAS = visual analogue scale.

^a^ For ‘Happy’, adults had higher ratings than adolescents on ‘PLA’, but similar ratings on ‘THC’ and ‘THC + CBD’. Moreover, within adolescents, ratings during ‘THC’ and ‘THC + CBD’ were higher than ‘PLA’, but within adults ratings during ‘THC’ and ‘THC + CBD’ were lower than during ‘PLA’.

^b^ For CBD plasma levels, this interaction was driven by higher CBD levels on ‘THC + CBD’ in adults compared to adolescents, but similar levels in adults and adolescents during ‘PLA’ or ‘THC’.

Exceptions to this pattern included the following results. There were drug by age‐group interactions for ‘happy’ (across T1–T5; Figure S5g‐i) and CBD plasma levels (T2; Figure S7 and Table S17b). There were significant differences between active drug conditions (‘THC + CBD’ > ‘THC’) for: ‘feel drug effect’ (across T2–T5; Figure S4a–c), PSI subscale cognitive disorganisation (Table S14), heart rate (Figure S7e,f) and THC and CBD plasma levels (at T2) (Figure S7a–d; Table S17b). Notably, T2 plasma THC levels were lower on ‘THC’ (14.88 ng/mL; SD = 7.15) than ‘THC + CBD’ (27.86 ng/mL; SD = 14.20) (*t*(90.514) = 7.760, *P* < 0.001).

For subjective effects over time, there was no drug by age group‐by‐time interactions for any secondary outcome (Tables S10 and 11). For peak subjective effects and area‐under‐the‐curve data, there were no drug by age‐group interactions, and the pattern of results was mainly consistent with the previous analyses. Detailed results for all secondary outcomes are presented in the Supporting Information.

## DISCUSSION

We carried out the first controlled experiment testing the acute effects of vaporised cannabis with and without cannabidiol in both adolescents and adults. On our three primary outcomes, subjective ‘feel drug effect’, verbal episodic memory and psychotomimetic effect, we found no evidence that age group moderated the impact of cannabis. Furthermore, Bayesian analyses supported these null results. Additionally, we found no evidence that vaporised CBD (24 mg for a 75 kg person) modulated the acute impact of vaporised THC (8 mg for a 75 kg person) on our primary outcome variables. Our results were supported by analysis of our secondary outcome variables, which showed, almost unanimously, that there were null moderating effects of age group and CBD. The mostly consistent pattern of results was (i) ‘THC’ and ‘THC + CBD’ produced expected changes from ‘PLA’; (ii) ‘THC’ and ‘THC + CBD’ did not differ from one another; and (iii) the impact of cannabis did not differ in adolescents and adults.

Based on Mokrysz *et al*. [38], we hypothesised that adolescents would be more resilient to the acute effects of cannabis than adults. We did not find evidence for this; indeed, our Bayesian analyses supported equivalence of the impact of cannabis on adolescents and adults in all three of our primary outcome variables. This partially supports Murray *et al*. [39], which reported no differences in the impact of oral THC (fixed, 7.5 and 15 mg doses) on 18‐ to 20‐year‐olds and 30‐ to 40‐year‐olds on all subjective effects recorded, cardiovascular measures, working memory and response inhibition. In comparison to Mokrysz *et al*. [38], in which adolescents used cannabis significantly more frequently (median, 2.4 days/week) than adults (median, 1.8 days/week), our adolescent and adult participants were matched on cannabis use frequency. The discrepancy in our findings may have been partially due to the greater frequency of cannabis use in the adolescent group in the previous experiment, therefore, contributing to a higher tolerance to THC's effects [57]. Furthermore, in the experiment conducted by Mokrysz *et al*. [38], adolescents weighed significantly less and received an absolutely lower dose of THC than adults.

On our primary outcome variables, CBD did not significantly moderate the acute effects of THC. This is in line with previous controlled cannabis vaporisation experiments [34, 37, 58]. Significant modulation of THC's acute effects may require pre‐dosing with larger oral [36] or intravenous [35] CBD. Additionally, stronger THC‐induced psychotomimetic effects may be necessary before the protective effect of CBD is observed [59].

However, there were some notable exceptions to the primarily null findings. First, there was some limited evidence that ‘THC + CBD’ produced stronger effects than ‘THC’: ‘feel drug effect’ was half a point greater on ‘THC + CBD’ compared to ‘THC’, when collapsing across all timepoints, and this was supported by an area‐under‐the‐curve difference too; the PSI cognitive disorganisation subscale was greater by ~2 points on ‘THC + CBD’ compared to ‘THC’; and heart rate was greater on ‘THC + CBD’ than ‘THC’ by 8 beats/min. To contextualise these differences, the ‘THC’ versus ‘PLA’ conditions gave differences of 4.5 points, 4 points and 18 beats/min, respectively. Notably, these were secondary outcome variables. They should also be considered against a backdrop of null CBD moderation effects on ‘like drug’, ‘dislike drug’, ‘paranoid’, ‘want cannabis’, all other PSI subscales, prose recall performance and both positive and negative PANSS scores. The supplementary analyses of area‐under‐the‐curve and peak subjective effects (Supporting Information, Section 3.5.2) confirmed there was no significant moderation by CBD, apart from on ‘feel drug effect’. On the whole, CBD did not appear to moderate THC's acute effects.

Mean THC plasma concentrations at T2 for ‘THC’ (15 ng/mL) and ‘THC + CBD’ (28 ng/mL) conditions (Table S17b) are in line with some previous THC vaporisation studies [60, 61], but slightly lower than other studies [62, 63]. Mean plasma CBD concentration at T2 for the ‘THC + CBD’ condition was 55 ng/mL, with zero or trace levels in the ‘THC’ condition. Some [61, 63, 64], but not all [60, 65], previous research has reported that CBD co‐administration enhances THC plasma levels. It is unclear why THC levels were greater under the ‘THC + CBD’ condition relative to the ‘THC’ condition. A pharmacokinetic explanation, whereby CBD augments THC levels by inhibiting the metabolism of THC [66], is possible. However, another possible explanation is the difference in inhalation time. Participants took significantly longer to inhale the ‘THC + CBD’ vapour and coughed significantly more during ‘THC + CBD’ compared with ‘THC’. Our supplementary analysis (Table S21) demonstrated that those who took longer to complete the second balloon had higher plasma THC levels at T2, therefore, partially explaining the impact of CBD on plasma THC levels. Future, detailed analyses of our pharmacokinetic data may shed light on this, but this is beyond the scope of the current manuscript. Despite the greater plasma THC levels in the ‘THC + CBD’ condition, we did not observe stronger effects on our primary outcome variables. Speculatively, this could be because CBD pharmacokinetically increases THC levels, but partially pharmacodynamically blocks THC's effects. Alternatively, there may not be a simple linear relationship between plasma THC levels and subjective, psychological effects [67, 68, 69]. Additional research into the pharmacokinetics of co‐administered THC and CBD is required.

We also detected an age‐group by drug interaction for CBD plasma level, in which adults had greater CBD levels than adolescents (*P* < 0.001; MD = 21 ng/mL). Adults weighed slightly more than the adolescents and therefore, received more CBD (20 mg vs 22 mg), although that difference was not statistically significant. The two age groups took similar times to inhale the balloons. The variance in CBD plasma levels was much greater in adults than adolescents, with many adults surpassing 100 ng/mL, but no adolescents surpassing 80 ng/mL. The reason for this difference is unknown. This age difference in CBD absorption appears not to have meaningfully altered our psychological outcomes. We only found one significant drug by age‐group interaction, on ‘happy’. This unhypothesised interaction was driven by adults being slightly happier on placebo than active drug, whereas adolescents were slightly happier on active drugs than on placebo.

Our study has numerous strengths. In addition to its novelty, we biochemically verified drug abstinence; we improved on previous work by matching adolescent and adult users on cannabis use; including equal numbers of men and women; and we powered our study to detect an age‐group by drug interaction and took account of winner's curse [55]. One limitation is the absence of a CBD‐only condition. A further limitation is that our results may not generalise beyond adolescents ages 16 to 17 years and adults ages 26 to 29 years, who use cannabis 0.5–3 days/week. It is possible that younger adolescents or older adults could respond differently to the administration of cannabis. Moreover, it is possible that some age differences may be apparent in less frequent [39] or more frequent cannabis users.

Our findings have important scientific, policy and educational implications. First, they cast doubt on the claim that adolescent cannabis users are more vulnerable to some short‐term effects of cannabis than adult cannabis users. Second, our results add further weight to the claim that doses of vaporised CBD, which are near to naturally occurring levels, do not mitigate the acute harms of THC [34, 61]. Harm reduction messages should avoid stating that CBD found in recreational cannabis protects against the acute psychotomimetic or verbal memory‐impairing effects of cannabis.

## AUTHOR CONTRIBUTIONS

**Will Lawn**: Conceptualisation, data curation, formal analysis, investigation, methodology, project administration, supervision, validation, visualisation, writing—original draft and writing—review and editing. **Katie Trinci**: Data curation, formal analysis, investigation, project administration, validation, visualisation, writing—original draft and writing—review and editing. **Claire Mokrysz**: Conceptualisation, data curation, investigation, funding acquisition, methodology, project administration, supervision and writing—review and editing. **Anna Borissova**: Conceptualisation, formal analysis, investigation, methodology, project administration, supervision and writing—review and editing. **Shelan Ofori**—Investigation, project administration, writing—original draft, and writing—review and editing. **Kat Petrilli**: investigation, project administration, methodology, writing—original draft and writing—review and editing. **Michael Bloomfield**: Conceptualisation, supervision and writing—review and editing. **Zarah R. Haniff**: Investigation, project administration, writing—original draft and writing—review and editing. **Daniel Hall**: Investigation, project administration, writing—original draft and writing—review and editing. **Natalia Fernandez‐Vinson**: Investigation, project administration, writing—original draft and writing—review and editing. **Simiao Wang**: Project administration, writing—original draft and writing—review and editing. **Amir Englund**: Conceptualisation, methodology and writing—review and editing. **Edward Chesney**: Conceptualisation, methodology and writing—review and editing. **Matthew B. Wall**: Conceptualisation, formal analysis, investigation, methodology, project administration, supervision and writing—review and editing. **Tom P. Freeman**: Conceptualisation, funding acquisition, investigation, methodology, project administration, supervision, validation, visualisation, writing—original draft and writing—review and editing. **H. Valerie Curran**: Conceptualisation, funding acquisition, investigation, methodology, project administration, supervision, validation, visualisation, writing—original draft and writing—review and editing.

## CLINICAL TRIAL REGISTRATION DETAILS

Although not deemed to be a clinical trial by the UK Medicines and Healthcare products Regulatory Agency, given international variations in defining a clinical trial, this study was registered on Clinical.Trials.gov (NCT04851392).

## PROTOCOL AND ANALYSIS PRE‐REGISTRATION

https://osf.io/z638r/

https://osf.io/v4d5x/

## DECLARATION OF INTERESTS

W.L., K.T., C.M., A.B., S.O., M.B., Z.H., D.H., N.F.V., S.W., A.E., T.P.F. and H.V.C. declare no competing interests. A.E. has received speaker's honoraria from Jazz Pharmaceuticals. M.W. is primarily employed by Invicro.

## Supporting information


**Figure S1.** Drug administration session schedule indicating when outcome measures were assessed, where time‐inh = start of drug inhalation. Administration 1 and 2 = inhalation of first and second balloon filled with cannabis vapour (‘THC’, ‘THC + CBD’ or ‘PLA’), respectively. Relative to time‐inh: T1 = −5 min, T2 = +20 min, T3 = +30 min, T4 = +120 min and T5 = +160 min. Adm = administration, imm = immediate, del = delayed, min = minutes, VAS = visual analogue scale, PSI = psychotomimetic states inventory, MRI = magnetic resonance imaging, PANSS=Positive and Negative Syndrome scale.
**Figure S2.** Example of subjective effects visual analogue scale (VAS) for a single response variable.
**Figure S3.** Consolidated Standards of Reporting Trials (CONSORT) flowchart for all participants. Participants' eligibility was first assessed online and subsequently on the telephone, when we recorded reasons for ineligibility. Participants' eligibility was further checked at an in‐person or video‐call baseline session. Participants who passed screening and baseline sessions were randomly allocated a drug order. We continued the experiment until 48 participants had completed all three drug administration sessions, in a per‐protocol fashion. Participants who dropped out after one or two drug administration sessions were excluded. Other reasons for dropping out include scheduling conflicts, personal reasons, and no reason given. COVID‐related restrictions were primarily because of government restrictions in March 2020 (after which the study was paused for seven months) and January 2021.
**Figure S4.** Mean values for subjective effect VASs (‘feel drug effect’, ‘like drug effect’, and ‘dislike drug effect’) with 95% confidence intervals over time for adolescents and adults (*n* = 24 per group for full datasets) during ‘PLA’, ‘THC’ and ‘THC + CBD’ conditions. VASs ranged from 0 = ‘not at all’ to 10 = ‘extremely’. Data from the two age‐groups has been combined to show overall drug effects in c, f and i (*n* = 48). Relative to the start of drug inhalation (time‐inh) the timepoints correspond as following: T2 = +20 min, T3 = +30 min, T4 = +120 min, and T5 = +160 min. Subjective effect VASs was not collected before cannabis inhalation (−5mins*).* VAS = visual analogue scale. Data is missing for 1 adult during T5 of ‘THC + CBD’.
**Figure S5.** Mean values for subjective effect VASs (‘anxious’, ‘paranoid’, ‘want cannabis’, and ‘happy’) with 95% confidence intervals over time for adolescents and adults (*n* = 24 per group for full datasets) during ‘PLA’, ‘THC’ and ‘THC + CBD’ conditions. VASs ranged from 0 = ‘not at all’ to 10 = ‘extremely’. Data from the two age groups has been combined to show overall drug effects in c, f, i, and l (*n* = 48). Relative to the start of drug inhalation (time‐inh) the timepoints correspond as following: T1 = −5 min, T2 = +20 min, T3 = +30 min, T4 = +120 min, and T5 = +160 min. VAS = visual analogue scale. Data is missing for 1 adult during T5 of ‘THC + CBD’.
**Figure S6.** PANSS positive and negative subscale score, presented as mean values with 95% confidence intervals for adolescents (*n* = 24) and adults *(n* = 24) during
**Figure S7.** Mean values with 95% confidence intervals displayed with all data points for a) THC plasma levels at T2, c) CBD plasma levels at T2 and e) heart rate at T2 for adolescents and adults during ‘THC’, ‘THC + CBD’ and ‘PLA’ conditions. Main effect of drug across age‐groups displayed in b, d, and f where their data is combined. **p < 0.01, ***p < 0.001. Relative to the start of drug administration T2 = +20 minutes. Bpm = beats per minute.
**Table S1.** Inclusion and exclusion criteria for all participants, where criteria only relate to specific group (e.g. adolescent/adult/female/male) this is stated within the table.
**Table S2.** Number of participants with missing data for all variables *(n).*
**Table S3.** Number of extreme outliers for all outcome.
**Table S4.** Baseline and demographic data. Either *n* (%) or mean (SD) [min‐max].
**Table S5.** Cannabis use information. Either *n* (%) or mean (SD) [min‐max].
**Table S6.** Lifetime drug use variables. *N* (%).
**Table S7.** Sociodemographic and cannabis use information of participant**s** who dropped‐out, compared with participants who were included. Either n (%) or mean (SD).
**Table S8.** Time since last cannabis use (hours) before each session. Mean (SD) [min – max].
**Table S9.** Descriptive statistics of primary outcomes.
**Table S10a‐g.** Results from 2x3x4 and 2x3x5 mixed ANOVA analyses of subjective effect VASs, showing main effects and interactions. Where each analysis had a within subject factors of drug (‘PLA’, ‘THC’, and ‘THC + CBD’) and time (T1‐T5, or T2‐T5*) and a between subject factor of age‐group (adolescent, *n* = 24; and adult *n* = 24). ‘Feel drug effect’, ‘like drug effect’ and ‘dislike drug effect’ were completed T2‐T5; ‘anxious’, ‘paranoid’, ‘want cannabis’ and ‘happy' were completed T1‐T5.
**Table S11.** Prose recall immediate and delayed 2x2x3 mixed ANOVA, with within subject factors of time (immediate and delayed*) and drug (‘PLA’, ‘THC’, and ‘THC + CBD’), and a between subject factor of age‐group (adolescent, *n* = 24; and adult *n* = 24).
**Table S12.** PSI subscale scores for adolescents (*n* = 24), adults (*n* = 24), and both (*n* = 48) across each drug session. Mean (SD), [median, min‐max].
**Table S13.** Two‐way mixed ANOVA results for PSI subscale scores with a within subjects factor of drug (‘PLA’, ‘THC’, and ‘THC + CBD’) and a between subjects factor of age‐group (adolescent, *n* = 24; and adult *n* = 24).
**Table S14.** Pairwise comparisons (following mixed‐ANOVA) of each PSI subscale scores between drug conditions following a main effect of drug.
**Table S15.** Descriptive Statistics for PANSS Positive and Negative subscale scores across each drug condition for adolescents (*n* = 24) and adults (*n* = 24), mean (SD) [median, min‐max].
**Table S16.** Clinically significant psychotic reaction on PANSS positive and negative (i.e. score >3 greater than minimum) for adolescents (*n* = 24) and adults (*n* = 24). *n* (%) is shown.
‘THC’, ‘THC + CBD’ and ‘PLA’ conditions. Main effect of drug across age‐groups displayed in ‘Both’ (b and d) graphs where participant data is combined (*n* = 48). **P < 0.01, ***P < 0.001. PANSS = positive and negative syndrome scale.
**Table S17a.** Plasma THC and CBD concentrations (ng.ml^−*1*^) in adolescents and adults at T1 (5 minutes before start of cannabis inhalation). Adolescent (*n* = 24), adult (*n* = 24) and both (*n* = 48) for complete datasets. Mean (SD) [median, range].
**Table S17b.** Plasma THC and CBD concentrations (ng.ml^*−*1*)*^ in adolescents and adults at T2 (20 minutes after start of cannabis inhalation). Adolescent (*n* = 24), adult (*n* = 24) and both (*n* = 48) for complete datasets. Mean (SD) [range].
**Table S18.** Heart rate at T2 (20 minutes after beginning cannabis inhalation). Adolescent (*n* = 24), adult (*n* = 24) for complete datasets. Mean (SD) [min‐max].
**Table S19.** Participants' guess regarding which drug they received at the end of each drug administration session. Adolescent (*n* = 24), adult (*n* = 24) for complete datasets. n(%).
**Table S20.** Cumulative time taken (minutes) to inhale both balloons filled with cannabis vapour during each session. Adolescents (*n* = 24) and adults (*n* = 24) unless otherwise stated. Mean (SD), [median, min‐max].
**Table S21.** Multilevel model results for THC plasma levels (T2)

## References

[1] NHS Digital . Smoking, drinking and drug use among young people in England in 2021 NHS Digital; 2022.

[2] NHS Digital . Smoking, drinking and drug use among young people in England in 2018 NHS Digital; 2019. https://digital.nhs.uk/data-and-information/publications/statistical/smoking-drinking-and-drug-use-among-young-people-in-england/2018

[3] The University of Michigan . Monitoring the future. National Institute on Drug Abuse. 2020. 10.3389/fdgth.2020.60892

[4] Degenhardt L , Chiu WT , Sampson N , Kessler RC , Anthony JC , Angermeyer M , et al. Toward a global view of alcohol, tobacco, cannabis, and cocaine use: Findings from the WHO world mental health surveys. PLoS Med. 2008;5(7):e141. 10.1371/journal.pmed.0050141 18597549 PMC2443200

[5] Chen CY , O'Brien MS , Anthony JC . Who becomes cannabis dependent soon after onset of use? Epidemiological evidence from the United States: 2000–2001. Drug Alcohol Depend. 2005;79(1):11–22. 10.1016/j.drugalcdep.2004.11.014 15943940

[6] Volkow ND , Han B , Einstein EB , Compton WM . Prevalence of substance use disorders by time since first substance use among young people in the US. JAMA Pediatr. 2021:640–643. 10.1001/jamapediatrics.2020.6981 33779715 PMC8008418

[7] Lawn W , Mokrysz C , Lees R , Trinci K , Petrilli K , Skumlien M , et al. The CannTeen study: Cannabis use disorder, depression, anxiety, and psychotic‐like symptoms in adolescent and adult cannabis users and age‐matched controls. 2022. 10.1177/02698811221108956 PMC9716489 35772419

[8] Hall W , Lynskey M . Assessing the public health impacts of legalizing recreational cannabis use: The US experience. World Psychiatry. 2020;19(2):179–86. 10.1002/wps.20735 32394566 PMC7215066

[9] Sarvet AL , Wall MM , Keyes KM , Cerdá M , Schulenberg JE , O'Malley PM , et al. Recent rapid decrease in adolescents' perception that marijuana is harmful, but no concurrent increase in use. Drug Alcohol Depend. 2018;186:68–74. 10.1016/j.drugalcdep.2017.12.041 29550624 PMC6134844

[10] Cerdá M , Mauro C , Hamilton A , Levy NS , Santaella‐Tenorio J , Hasin D , et al. Association between recreational marijuana legalization in the United States and changes in marijuana use and cannabis use disorder from 2008 to 2016. JAMA Psychiat. 2020;77(2):165–71. 10.1001/jamapsychiatry.2019.3254 PMC6865220 31722000

[11] Wilson J , Freeman TP , Mackie CJ . Effects of increasing cannabis potency on adolescent health. Lancet Child Adolesc Health. 2019;3(2):121–8. 10.1016/S2352-4642(18)30342-0 30573419

[12] Blakemore S , Choudhury S . Development of the adolescent brain: Implications for executive function and social cognition. J Child Psychol Psychiatry. 2006;47(3–4):296–312. 10.1111/j.1469-7610.2006.01611.x 16492261

[13] Dumontheil I . Adolescent brain development. Curr Opin Behav Sci. 2016;10:39–44. 10.1016/j.cobeha.2016.04.012

[14] Giedd JN , Blumenthal J , Jeffries NO , Castellanos FX , Liu H , Zijdenbos A , et al. Brain development during childhood and adolescence: A longitudinal MRI study. Nat Neurosci. 1999;2(10):861–3. 10.1038/13158 10491603

[15] Shaffer DR , Kipp K . Developmental psychology: Childhood and adolescence. 2010.

[16] Meyer HC , Lee FS , Gee DG . The role of the endocannabinoid system and genetic variation in adolescent brain development. Neuropsychopharmacology. 2018;43(1):21–33. 10.1038/npp.2017.143 28685756 PMC5719094

[17] Galve‐Roperh I , Palazuelos J , Aguado T , Guzmán M . The endocannabinoid system and the regulation of neural development: Potential implications in psychiatric disorders. Eur Arch Psychiatry Clin Neurosci. 2009;259(7):371–82. 10.1007/s00406-009-0028-y 19588184

[18] Kessler RC , Berglund P , Demler O , Jin R , Merikangas KR , Walters EE . Lifetime prevalence and age‐of‐onset distributions of DSM‐IV disorders in the National Comorbidity Survey Replication. Arch Gen Psychiatry. 2005;62(6):593–602. 10.1001/archpsyc.62.6.593 15939837

[19] Paus T , Keshavan M , Giedd JN . Why do many psychiatric disorders emerge during adolescence? Nat Rev Neurosci. 2008;9(12):947–57. 10.1038/nrn2513 19002191 PMC2762785

[20] Marconi A , Di Forti M , Lewis CM , Murray RM , Vassos E . Meta‐analysis of the association between the level of cannabis use and risk of psychosis. Schizophr Bull. 2016;42(5):1262–9. 10.1093/schbul/sbw003 26884547 PMC4988731

[21] Gobbi G , Atkin T , Zytynski T , Wang S , Askari S , Boruff J , et al. Association of cannabis use in adolescence and risk of depression, anxiety, and suicidality in young adulthood: A systematic review and meta‐analysis. JAMA Psychiat. 2019;76(4):426–34. 10.1001/jamapsychiatry.2018.4500 PMC6450286 30758486

[22] Lorenzetti V , Hoch E , Hall W . Adolescent cannabis use, cognition, brain health and educational outcomes: A review of the evidence. Eur Neuropsychopharmacol. 2020;36:169–80. 10.1016/j.euroneuro.2020.03.012 32268974

[23] Hall W , Leung J , Lynskey M . The effects of cannabis use on the development of adolescents and young adults. Ann Rev Dev Psychol. 2020;2(1):461–83. 10.1146/annurev-devpsych-040320-084904

[24] Curran VH , Brignell C , Fletcher S , Middleton P , Henry J . Cognitive and subjective dose‐response effects of acute oral Δ 9‐tetrahydrocannabinol (THC) in infrequent cannabis users. Psychopharmacology (Berl). 2002;164(1):61–70. 10.1007/s00213-002-1169-0 12373420

[25] D'Souza DC , Perry E , MacDougall L , Ammerman Y , Cooper T , Wu Y , et al. The psychotomimetic effects of intravenous delta‐9‐tetrahydrocannabinol in healthy individuals: Implications for psychosis. Neuropsychopharmacology. 2004;29(8):1558–72. 10.1038/sj.npp.1300496 15173844

[26] D'Souza DC , Ranganathan M , Braley G , Gueorguieva R , Zimolo Z , Cooper T , et al. Blunted psychotomimetic and amnestic effects of Δ‐9‐tetrahydrocannabinol in frequent users of cannabis. Neuropsychopharmacology. 2008;33(10):2505–16. 10.1038/sj.npp.1301643 18185500 PMC3799954

[27] Green BOB , Kavanagh D , Young R . Being stoned: A review of self‐reported cannabis effects. Drug Alcohol Rev. 2003;22(4):453–60. 10.1080/09595230310001613976 14660135

[28] Pertwee R . The diverse CB1 and CB2 receptor pharmacology of three plant cannabinoids: Δ9‐tetrahydrocannabinol, cannabidiol and Δ9‐tetrahydrocannabivarin. Br J Pharmacol. 2008;153(2):199–215. 10.1038/sj.bjp.0707442 17828291 PMC2219532

[29] Britch SC , Babalonis S , Walsh SL . Cannabidiol: Pharmacology and therapeutic targets. Psychopharmacology (Berl). 2021;238(1):9–28. 10.1007/s00213-020-05712-8 33221931 PMC7796924

[30] Russo EB , Burnett A , Hall B , Parker KK . Agonistic properties of cannabidiol at 5‐HT1a receptors. Neurochem Res. 2005;30(8):1037–43. 10.1007/s11064-005-6978-1 16258853

[31] Laprairie RB , Bagher AM , Kelly MEM , Denovan‐Wright E . Cannabidiol is a negative allosteric modulator of the cannabinoid CB1 receptor. Br J Pharmacol. 2015;172(20):4790–805. 10.1111/bph.13250 26218440 PMC4621983

[32] Freeman AM , Petrilli K , Lees R , Hindocha C , Mokrysz C , Curran HV , et al. How does cannabidiol (CBD) influence the acute effects of delta‐9‐tetrahydrocannabinol (THC) in humans? A systematic review. Neurosci Biobehav Rev. 2019;107:696–712. 10.1016/j.neubiorev.2019.09.036 31580839

[33] Englund A , Morrison PD , Nottage J , Hague D , Kane F , Bonaccorso S , et al. Cannabidiol inhibits THC‐elicited paranoid symptoms and hippocampal‐dependent memory impairment. J Psychopharmacol. 2013;27(1):19–27. 10.1177/0269881112460109 23042808

[34] Morgan CJA , Freeman TP , Hindocha C , Schafer G , Gardner C , Curran HV . Individual and combined effects of acute delta‐9‐tetrahydrocannabinol and cannabidiol on psychotomimetic symptoms and memory function. Transl Psychiatry. 2018;8(1):1–10. 30185793 10.1038/s41398-018-0191-x PMC6125482

[35] Bhattacharyya S , Morrison PD , Fusar‐Poli P , Martin‐Santos R , Borgwardt S , Winton‐Brown T , et al. Opposite effects of Δ‐9‐tetrahydrocannabinol and cannabidiol on human brain function and psychopathology. Neuropsychopharmacology. 2010;35(3):764–74. 10.1038/npp.2009.184 19924114 PMC3055598

[36] Englund A , Morrison PD , Nottage J , Hague D , Kane F , Bonaccorso S , et al. Cannabidiol inhibits THC‐elicited paranoid symptoms and hippocampal‐dependent memory impairment. J Psychopharmacol. 2013;27(1):19–27. 10.1177/0269881112460109 23042808

[37] Mokrysz C , Shaban NDC , Freeman TP , Lawn W , Pope RA , Hindocha C , et al. Acute effects of cannabis on speech illusions and psychotic‐like symptoms: Two studies testing the moderating effects of cannabidiol and adolescence. Psychol Med. 2021;51(12):2134–42. 10.1017/S0033291720001038 32340632

[38] Mokrysz C , Freeman TP , Korkki S , Griffiths K , Curran HV . Are adolescents more vulnerable to the harmful effects of cannabis than adults? A placebo‐controlled study in human males. Transl Psychiatry. 2016;6(11):e961–1. 10.1038/tp.2016.225 27898071 PMC5290352

[39] Murray CH , Huang Z , Lee R , de Wit H . Adolescents are more sensitive than adults to acute behavioral and cognitive effects of THC. Neuropsychopharmacology. 2022;47(7):1331–8. 10.1038/s41386-022-01281-w 35110688 PMC9117219

[40] Treloar Padovano H , Miranda R Jr . Subjective cannabis effects as part of a developing disorder in adolescents and emerging adults. J Abnorm Psychol. 2018;127(3):282. 10.1037/abn0000342 29672090 PMC5912694

[41] Schramm‐Sapyta NL , Cha YM , Chaudhry S , Wilson WA , Swartzwelder HS , Kuhn CM . Differential anxiogenic, aversive, and locomotor effects of THC in adolescent and adult rats. Psychopharmacology (Berl). 2007;191(4):867–77. 10.1007/s00213-006-0676-9 17211649

[42] Cha YM , White AM , Kuhn CM , Wilson WA , Swartzwelder HS . Differential effects of delta9‐THC on learning in adolescent and adult rats. Pharmacol Biochem Behav. 2006;83(3):448–55. 10.1016/j.pbb.2006.03.006 16631921

[43] Lawn W , Mokrysz C , Ofori S , Trinci K , Borissova A , Petrilli K , et al. Do adolescents and adults differ in their acute subjective, behavioural, and neural responses to cannabis, with and without cannabidiol? CannTeenA [Internet]. OSF. 2021 [cited 2022 Jul 12]. Available from: Do adolescents and adults differ in their acute subjective, behavioural, and neural responses to cannabis, with and without cannabidiol? CannTeenA.

[44] Heatherton TF , Kozlowski LT , Frecker RC , Rickert W , Robinson J . Measuring the heaviness of smoking: Using self‐reported time to the first cigarette of the day and number of cigarettes smoked per day. Br J Addict. 1989;84(7):791–800. 10.1111/j.1360-0443.1989.tb03059.x 2758152

[45] van der Pol P , Liebregts N , de Graaf R , Korf DJ , van den Brink W , van Laar M . Validation of self‐reported cannabis dose and potency: An ecological study. Addiction. 2013;108(10):1801–8. 10.1111/add.12226 23627816

[46] Freeman TP , Morgan CJA , Hindocha C , Schafer G , Das RK , Curran HV . Just say ‘know’: How do cannabinoid concentrations influence users' estimates of cannabis potency and the amount they roll in joints? Addiction. 2014;109(10):1686–94. 10.1111/add.12634 24894801

[47] Freeman TP , Lorenzetti V. A standard THC unit for reporting of health research on cannabis and cannabinoids. 2021. https://www.thelancet.com/journals/lanpsy/article/PIIS2215-0366(21)00355-2/fulltext 10.1016/S2215-0366(21)00355-2 34506750

[48] Freeman TP , Lorenzetti V. Standard THC units: a proposal to standardize dose across all cannabis products and methods of administration. 2019. https://onlinelibrary.wiley.com/doi/full/10.1111/add.14842?casa_token=hTI8t_g_SWwAAAAA%3Ah5BiRMDjJcgGzmQ3XoXOM76qujR‐_qFuKAzPQQPD0KNIphcQ2VFIlTj0vqSZi76EFVfOpEQPQD5GkooL 10.1111/add.14842 31606008

[49] D'souza DC , Fridberg DJ , Skosnik PD , Williams A , Roach B , Singh N , et al. Dose‐related modulation of event‐related potentials to novel and target stimuli by intravenous Δ9‐THC in humans. Neuropsychopharmacology. 2012;37(7):1632–46. 10.1038/npp.2012.8 22334121 PMC3358754

[50] Heuberger J , Guan Z , Oyetayo O , Klumpers L , Morrison P , Beumer T , et al. Population pharmacokinetic model of THC integrates oral, intravenous, and pulmonary dosing and characterizes short‐ and long‐term pharmacokinetics. Clin Pharmacokinet. 2015;54(2):209–19. 10.1007/s40262-014-0195-5 25316574

[51] Mørland J , Bramness JG . Δ9‐tetrahydrocannabinol (THC) is present in the body between smoking sessions in occasional non‐daily cannabis users. Forensic Sci Int. 2020;309:110188. 10.1016/j.forsciint.2020.110188 32120192

[52] Baddeley A , Wilson BA . Prose recall and amnesia: Implications for the structure of working memory. Neuropsychologia. 2002;40(10):1737–43. 10.1016/S0028-3932(01)00146-4 11992661

[53] Mason OJ , Morgan CJM , Stefanovic A , Curran HV . The psychotomimetic states inventory (PSI): Measuring psychotic‐type experiences from ketamine and cannabis. Schizophr Res. 2008;103(1–3):138–42. 10.1016/j.schres.2008.02.020 18387788

[54] Kay SR , Fiszbein A , Opler LA . The positive and negative syndrome scale (PANSS) for schizophrenia. Schizophr Bull. 1987;13(2):261–76. 10.1093/schbul/13.2.261 3616518

[55] Button KS , Ioannidis JPA , Mokrysz C , Nosek BA , Flint J , Robinson ESJ , et al. Power failure: Why small sample size undermines the reliability of neuroscience. Nat Rev Neurosci. 2013;14(5):365–76. 10.1038/nrn3475 23571845

[56] Lawn W , Mokrysz C , Ofori S , Trinci K , Haniff Z , Hall D , et al. Do adolescents and adults differ in the acute subjective, psychotomimetic, and memory‐impairing effects of cannabis. Open Science Framework. 2021:1–11. https://osf.io/bm873

[57] Colizzi M , Bhattacharyya S . Cannabis use and the development of tolerance: A systematic review of human evidence. Neurosci Biobehav Rev. 2018;93:1–25. 10.1016/j.neubiorev.2018.07.014 30056176

[58] Lawn W , Freeman TP , Pope RA , Joye A , Harvey L , Hindocha C , et al. Acute and chronic effects of cannabinoids on effort‐related decision‐making and reward learning: An evaluation of the cannabis ‘amotivational’ hypotheses. Psychopharmacology (Berl). 2016;233(19–20):3537–52. 10.1007/s00213-016-4383-x 27585792 PMC5021728

[59] Freeman AM , Petrilli K , Lees R , Hindocha C , Mokrysz C , Curran HV , et al. How does cannabidiol (CBD) influence the acute effects of delta‐9‐tetrahydrocannabinol (THC) in humans? A systematic review. Neurosci Biobehav Rev. 2019;107:696–712. 10.1016/j.neubiorev.2019.09.036 31580839

[60] Arkell TR , Lintzeris N , Mills L , Suraev A , Arnold JC , McGregor IS . Driving‐related behaviours, attitudes and perceptions among Australian medical cannabis users: Results from the CAMS 18‐19 survey. Accid Anal Prev. 2020;148:105784. 10.1016/j.aap.2020.105784 33017729

[61] Arkell TR , Lintzeris N , Kevin RC , Ramaekers JG , Vandrey R , Irwin C , et al. Cannabidiol (CBD) content in vaporized cannabis does not prevent tetrahydrocannabinol (THC)‐induced impairment of driving and cognition. Psychopharmacology (Berl). 2019;236(9):2713–24. 10.1007/s00213-019-05246-8 31044290 PMC6695367

[62] Huestis MA . Human cannabinoid pharmacokinetics. Chem Biodivers. 2007;4(8):1770. 10.1002/cbdv.200790152 17712819 PMC2689518

[63] van de Donk T , Niesters M , Kowal MA , Olofsen E , Dahan A , van Velzen M . An experimental randomized study on the analgesic effects of pharmaceutical‐grade cannabis in chronic pain patients with fibromyalgia. Pain. 2019;160(4):860. 10.1097/j.pain.0000000000001464 30585986 PMC6430597

[64] Nadulski T , Pragst F , Weinberg G , Roser P , Schnelle M , Fronk EM , et al. Randomized, double‐blind, placebo‐controlled study about the effects of cannabidiol (CBD) on the pharmacokinetics of Δ9‐tetrahydrocannabinol (THC) after oral application of THC verses standardized cannabis extract. Ther Drug Monit. 2005;27(6):799–810. 10.1097/01.ftd.0000177223.19294.5c 16306858

[65] Solowij N , Broyd S , Greenwood L , van Hell H , Martelozzo D , Rueb K , et al. A randomised controlled trial of vaporised Δ9‐tetrahydrocannabinol and cannabidiol alone and in combination in frequent and infrequent cannabis users: Acute intoxication effects. Eur Arch Psychiatry Clin Neurosci. 2019;269(1):17–35. 10.1007/s00406-019-00978-2 30661105

[66] Hložek T , Uttl L , Kadeřábek L , Balíková M , Lhotková E , Horsley RR , et al. Pharmacokinetic and behavioural profile of THC, CBD, and THC+ CBD combination after pulmonary, oral, and subcutaneous administration in rats and confirmation of conversion in vivo of CBD to THC. Eur Neuropsychopharmacol. 2017;27(12):1223–37. 10.1016/j.euroneuro.2017.10.037 29129557

[67] Matheson J , Sproule B , di Ciano P , Fares A , le Foll B , Mann RE , et al. Sex differences in the acute effects of smoked cannabis: Evidence from a human laboratory study of young adults. Psychopharmacology (Berl). 2020;237(2):305–16. 10.1007/s00213-019-05369-y 31637452

[68] Theunissen EL , Kauert GF , Toennes SW , Moeller MR , Sambeth A , Blanchard MM , et al. Neurophysiological functioning of occasional and heavy cannabis users during THC intoxication. Psychopharmacology (Berl). 2012;220(2):341–50. 10.1007/s00213-011-2479-x 21975580 PMC3285765

[69] Martin‐Santos R , Crippa JA , Batalla A , Bhattacharyya S , Atakan Z , Borgwardt S , et al. Acute effects of a single, oral dose of d9‐tetrahydrocannabinol (THC) and cannabidiol (CBD) administration in healthy volunteers. Curr Pharm des. 2012;18(32):4966–79. 10.2174/138161212802884780 22716148

