# Supplementary material for: The acute effects of cannabis with and without cannabidiol in adults and adolescents: A randomised, double‐blind, placebo‐controlled, crossover experiment
Source: Addiction. 2023 Feb 26;118(7):1282–94. doi: 10.1111/add.16154 (PMC10481756; doi:10.1111/add.16154)
Supplement: Supplementary file 1 — Figure S1. Drug administration session schedule indicating when outcome measures were assessed, where time‐inh = start of drug inhalation. Administration 1 and 2 = inhalation of first and second balloon filled with cannabis vapour (‘THC’, ‘THC + CBD’ or ‘PLA’), respectively. Relative to time‐inh: T1 = −5 min, T2 = +20 min, T3 = +30 min, T4 = +120 min and T5 = +160 min. Adm = administration, imm = immediate, del = delayed, min = minutes, VAS = visual analogue scale, PSI = psychotomimetic states inventory, MRI = magnetic resonance imaging, PANSS=Positive and Negative Syndrome scale. Figure S2. Example of subjective effects visual analogue scale (VAS) for a single response variable. Figure S3. Consolidated Standards of Reporting Trials (CONSORT) flowchart for all participants. Participants' eligibility was first assessed online and subsequently on the telephone, when we recorded reasons for ineligibility. Participants' eligibility was further checked at an in‐person or video‐call baseline session. Participants who passed screening and baseline sessions were randomly allocated a drug order. We continued the experiment until 48 participants had completed all three drug administration sessions, in a per‐protocol fashion. Participants who dropped out after one or two drug administration sessions were excluded. Other reasons for dropping out include scheduling conflicts, personal reasons, and no reason given. COVID‐related restrictions were primarily because of government restrictions in March 2020 (after which the study was paused for seven months) and January 2021. Figure S4. Mean values for subjective effect VASs (‘feel drug effect’, ‘like drug effect’, and ‘dislike drug effect’) with 95% confidence intervals over time for adolescents and adults (n = 24 per group for full datasets) during ‘PLA’, ‘THC’ and ‘THC + CBD’ conditions. VASs ranged from 0 = ‘not at all’ to 10 = ‘extremely’. Data from the two age‐groups has been combined to show overall drug effects in c, f and i [file ADD-118-1282-s001.docx]

**Supplementary Materials**

**The acute effects of cannabis with and without cannabidiol in adults and adolescents: a randomised, double-blind, placebo-controlled, crossover experiment**

**Authors**

Will Lawn*, Katie Trinci* Claire Mokrysz, Anna Borissova, Shelan Ofori, Kat Petrilli, Michael Bloomfield, Zarah R Haniff, Daniel Hall, Natalia Fernandez-Vinson, Simiao Wang, Amir Englund, Edward Chesney, Matthew B Wall, Tom P Freeman, and H Valerie Curran

*Joint first authors

**Table** **of Contents**

[1. Methodological Information 2](#_Toc117778667)

[1.1 General supplemental methods 2](#_Toc117778668)

[**Table S1:** Inclusion and exclusion criteria for all participants 3](#_Toc117778669)

[**Figure S1:** Drug administration session schedule 5](#_Toc117778670)

[1.2 Blood sample collection & Plasma THC and CBD Quantification 5](#_Toc117778671)

[1.3 Baseline Variables 6](#_Toc117778672)

[1.4 Primary & Secondary Outcomes 7](#_Toc117778673)

[**Figure S2**. Example of Subjective Effects visual analogue scale (VAS) for a single response variable 7](#_Toc117778674)

[1.5 Statistical Analyses 8](#_Toc117778675)

[2. Data Pre-processing and Assumptions 11](#_Toc117778676)

[2.1 Missing data imputation 11](#_Toc117778677)

[**Table S2.** Number of participants with missing data for all variables (n). 12](#_Toc117778678)

[**Table S3.** Number of extreme outliers for all outcomes 13](#_Toc117778679)

[2.2 Unexpected subjective and plasma results 13](#_Toc117778680)

[2.3 Testing assumptions to conduct mixed-ANOVA on Primary Outcomes 14](#_Toc117778681)

[3. Results 15](#_Toc117778682)

[3.1 Participant Screening, recruitment and adverse events 15](#_Toc117778683)

[**Figure S3:** Consolidated Standards of Reporting Trials (CONSORT) flowchart for all participants. 16](#_Toc117778684)

[3.2 Baseline characteristics, cannabis, and other drug use variables 17](#_Toc117778685)

[**Table S4.** Baseline and demographic data**.** Either n (%) or mean (SD) [min-max]. 18](#_Toc117778686)

[**Table S5.** Cannabis use information. Either n (%) or mean (SD) [min-max]. 20](#_Toc117778687)

[**Table S6.** Lifetime drug use variables. N (%). 22](#_Toc117778688)

[**Table S7.** Sociodemographic and cannabis use information of participants who dropped-out, compared with participants who were included. Either n (%) or mean (SD). 23](#_Toc117778689)

[3.3 Time since last cannabis use 24](#_Toc117778690)

[**Table S8.** Time since last cannabis use (hours) before each session**.** Mean (SD) [min – max]. 24](#_Toc117778691)

[3.4 Primary Outcomes 25](#_Toc117778692)

[**Table S9.** Descriptive statistics of primary outcomes 25](#_Toc117778693)

[3.4.1 Results from secondary analyses of primary outcomes. 25](#_Toc117778694)

[3.5 Secondary outcome results 26](#_Toc117778695)

[3.5.1 Subjective Effects-VAS over time. 26](#_Toc117778696)

[**Figure S4.** Mean values for subjective effect VASs (‘feel drug effect’, ‘like drug effect’, and ‘dislike drug effect’) 27](#_Toc117778697)

[**Figure S5.** Mean values for subjective effect VASs (‘anxious’, ‘paranoid’, ‘want cannabis’, and ‘happy’) 29](#_Toc117778698)

[**Table S10a-g.** Results from 2x3x4 and 2x3x5 mixed ANOVA analyses of subjective effect VASs 30](#_Toc117778699)

[3.5.2 Area under curve (AUC) and peak analysis for Subjective Effects-VAS over time 32](#_Toc117778700)

[3.5.3 Prose Recall 34](#_Toc117778701)

[**Table S11.** Prose Recall Immediate and Delayed 2x2x3 mixed ANOVA, 34](#_Toc117778702)

[3.5.4 PSI subscales 34](#_Toc117778703)

[**Table S12.** PSI subscale scores 35](#_Toc117778704)

[**Table S13**. Two-way mixed ANOVA results for PSI subscale scores 36](#_Toc117778705)

[**Table S14.** Pairwise comparisons (following mixed-ANOVA) of each PSI subscale scores between drug conditions following a main effect of drug. 37](#_Toc117778706)

[3.5.5 PANSS 38](#_Toc117778707)

[**Table S15**. Descriptive Statistics for PANSS Positive and Negative subscale scores across each drug condition for adolescents (n=24) and adults (n=24), mean (SD) [median, min-max]. 39](#_Toc117778708)

[**Table S16**. Clinically significant psychotic reaction on PANSS 39](#_Toc117778709)

[**Figure S6**. PANSS Positive and Negative subscale score 40](#_Toc117778710)

[3.5.6 Plasma THC and CBD levels 41](#_Toc117778711)

[3.5.6.1 Plasma levels at T1 (pre-drug administration) 41](#_Toc117778712)

[**Table S17a.** Plasma THC and CBD concentrations (ng.ml^-1^) in adolescents and adults at T1 (5 minutes before start of cannabis inhalation). 41](#_Toc117778713)

[3.5.6.2 Plasma levels at T2 (20 minutes after the start of drug inhalation) 42](#_Toc117778714)

[**Table S17b**. Plasma THC and CBD concentrations (ng.ml^-1^) in adolescents and adults at T2 (20 minutes after start of cannabis inhalation). 42](#_Toc117778715)

[**Figure S7.** Mean values with 95% confidence intervals displayed with all data points for a) THC plasma levels at T2, c) CBD plasma levels at T2 and e) heart rate at T2 43](#_Toc117778716)

[3.5.7 Heart Rate (T2) 44](#_Toc117778717)

[**Table S18.** Heart rate at T2 (20 minutes after beginning cannabis inhalation). 44](#_Toc117778718)

[3.5.8 Participant Drug Guess 44](#_Toc117778719)

[**Table S19**. Participants’ guess regarding which drug they received at the end of each drug administration session. 44](#_Toc117778720)

[3.6 Drug Administration 45](#_Toc117778721)

[3.6.1 Deviations from inhalation procedure 45](#_Toc117778722)

[3.6.2 Time taken to inhale vapourised cannabis. 45](#_Toc117778723)

[**Table S20.** Cumulative time taken (minutes) to inhale both balloons filled with cannabis vapour during each session. 45](#_Toc117778724)

[3.6.3 Acute Coughing Rating 45](#_Toc117778725)

[3.7 Exploring impact of inhalation time on THC plasma levels 46](#_Toc117778726)

[**Table S21.** Multilevel model results for THC plasma levels (T2) 46](#_Toc117778727)

[4 Supplementary Discussion 47](#_Toc117778728)

[References 49](#_Toc117778729)

# **1. Methodological Information**

## 1.1 General supplemental methods

**Table S1:** Inclusion and exclusion criteria for all participants, where criteria only relate to specific group (e.g. adolescent/adult/female/male) this is stated within the table.

| **Inclusion criteria** | **Exclusion criteria** |
| --- | --- |
| **Adolescents**: aged 16-17 years  **Adults**: aged 26-29 years | **Females**: pregnant or breast-feeding |
| Self-reported cannabis use frequency of 0.5, 1, 2 or 3 days/week (averaged over the past 3 months) | **Adults**: before the age of 18 years, had a period of three months (or more) in which cannabis was consumed at a frequency of once per week (or more) |
| **Adults**: BMI between 18.5 and 29.9  **Adolescents**: BMI between 2^nd^ percentile and 98^th^ percentile | Severe cannabis use disorder (DSM-5 number of symptoms ≥ 6) |
| Willing to be cannulated and have four blood samples taken at every acute session | Illicit drug use of any one specific drug at a frequency of more than twice per month (averaged over the past 3 months) |
| Self-reported ability to consume approximately half a typical joint of cannabis by themselves within 20 minutes | Currently receiving pharmacological or psychological treatment for a mental health disorder (within the last month) |
| Right-handed | Lifetime psychosis |
|  | Lifetime psychosis in any immediate family member |
|  | Hypertension (systolic > 160; diastolic > 100) |
|  | Nicotine dependent (Heaviness of Smoking Index > 1) |
|  | Currently taking a psychotropic medication that will likely affect dependent variables or interact with cannabis |
|  | Any physical or mental health condition, any medication, or any treatment, that the study doctor considers to be an exclusion |
|  | MRI contraindications |
|  | Significant asthma or respiratory problems, severity judged clinically |
|  | Self-reported moderate/severe acute unpleasant effects from cannabis which occur often or always |
|  | Positive saliva drug screen at any acute session (including cannabis) (rearrange session) |
|  | Positive breathalyser reading at any acute session (rearrange session) |
|  | Self-reported alcohol use within 24 hours at any acute session (rearrange session) |
|  | Self-reported illicit drug use (including cannabis) within 72 hours at any acute session (rearrange session) |

Body Mass Index (BMI), Magnetic resonance imaging (MRI)

**Ethical Approval**

The study was deemed not to be a clinical trial by the UK Medicines and Healthcare products Regulatory Agency (MHRA), as it was not attempting to research the diagnosis, prevention, or treatment of a disease. Therefore, we did not require approval from a UK National Health Service Research Ethics Committee.

**Drug orders**

There were six drug orders: (1) ‘PLA’, ‘THC’, ‘THC+CBD’; (2) ‘PLA’, ‘THC+CBD’, ‘THC’; (3) ‘THC’, ‘PLA’, ‘THC+CBD’; (4) ‘THC’, ‘THC+CBD’, ‘PLA’; (5) ‘THC+CBD’, ‘PLA’, ‘THC’; (6) ‘THC+CBD’, ‘THC’, ‘PLA’.

**Cannabinoid content in each cannabis type**

Release certificates were provided by Bedrocan. The cannabis types we used had the following cannabinoid content: ‘Bedrocan’: 20.2% THC; 0.1% CBD; 0.1% CBN, ‘Bedrolite’: 0.4% THC; 8.5% CBD: <0.1% CBN, and ‘Bedrobinol Placebo: below detectable levels of any cannabinoid.

**Eligibility Drug & Alcohol tests**

**Breathalyzer Test:**Participants blood alcohol levels were assessed using a Lion Alcometer 500 breathalyzer at baseline, to check alcohol abstinence. Only those with a blood alcohol of 0 were allowed to participate in each session.

**Saliva drug tests:**Instant saliva drugs tests (either Alere DDSV 703 or ALLTEST DSD-867MET/C) were administered at baseline, assessing THC, opiates, benzodiazepine, methamphetamine, amphetamine and opiates in saliva. Only those with negative saliva tests for all drugs completed the session.

**Inhalation Procedure**

The prepared cannabis products were stored in identical, black opaque ‘filling chambers’ so the contents could not be seen by research staff or participants.

The prepared cannabis was vaporised at 210°C from the filling chamber into a ‘balloon’ using a Volcano Medic Vaporiser (Storz and Bickel, Tuttlingen, Germany). The balloon was covered in an opaque bag to ensure blinding.

The balloons’ contents were inhaled using a standardised inhalation procedure. Participants watch a timed PowerPoint presentation which instructed them to: inhale for 3 seconds, hold the inhalation for 6 seconds, exhale for 4 seconds, and pause (breathe normally) for 10 seconds. This was repeated until the balloon was empty. On figures and throughout the document, Time-inh = the start of inhalation of the first balloon. The start of inhalation of the second balloon was at +10 minutes. Participants were given nine minutes to inhale the contents of each balloon. Experimenters timed how long participants took to inhale the contents of each balloon. During inhalation participants were standing up.

This method of administration has been shown to be safe (1,2), and produce similar pulmonary and plasma cannabinoid levels to smoked cannabis, but with lower expired carbon monoxide levels (3–5).

The modal washout period between drug sessions was 7 days (mean=9.9, SD=6.9) and the maximum was 51 days. The mean number of days between the baseline and first drug session was 16.0 days (SD=8.4, range: 5-49).

**Participant reimbursement**

Participants were reimbursed £180 for completing the study. This was split into four payments: £35 payment following baseline, drug administration sessions 1 and 2, and £75 payment following the final drug administration session. Participants also won approximately £10 extra per drug administration session through the successful completion of tasks.


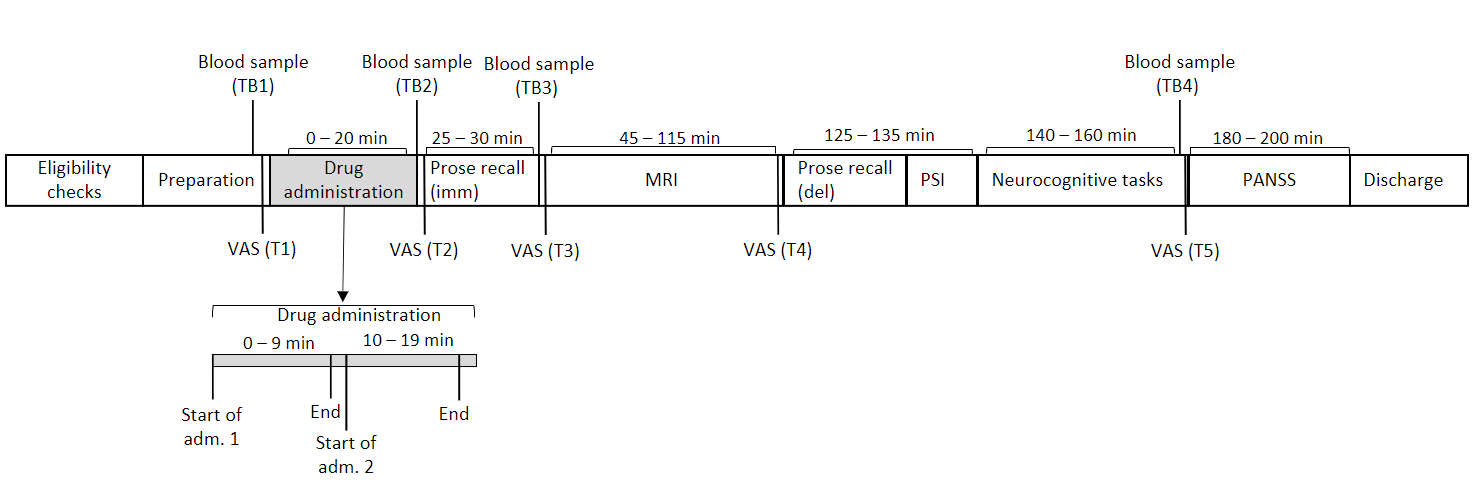


**Figure S1:** Drug administration session schedule indicating when outcome measures were assessed, where time-inh = start of drug inhalation. Administration 1 and 2 = inhalation of first and second balloon filled with cannabis vapour (‘THC’, ‘THC+CBD’ or ‘PLA’), respectively. Relative to time-inh: T1 = -5 min, T2 = +20 min, T3 = +30 min, T4 = +120 min, and T5 = +160 min. Adm=administration, imm=immediate, del=delayed, min= minutes, VAS=visual analogue scale, PSI=psychotomimetic states inventory, MRI=magnetic resonance imaging, PANSS=Positive and Negative Syndrome scale.

## 1.2 Blood sample collection & Plasma THC and CBD Quantification

Blood samples (2 x 6ml) were taken from participants via a cannula using BD Vacutainer® Heparin Tubes. Samples were inverted several times and centrifuged at 3000rpm for 10 minutes at 20°C, supernatant plasma was then decanted into four cryovials (1-2ml of plasma per cryovial) and stored at −80°C.

Cannabinoids were quantified by isotope-dilution gas chromatography-mass spectrometry (GC/MS) following solvent extraction from plasma, according to the following parameters:

- Standards and reagents: Acetonitrile, water, n-hexane and ethylacetate were of HPLC grade from Fisher Scientific, UK. *N*-*tert*-Butyldimethylsilyl-*N*-methyltrifluoroacetamide with 1% *tert*-Butyldimethylchlorosilane (MTBSTFA) was from Cerilliant via Merck-Sigma, UK
- Δ9-Tetrahydrocannabinol (Δ9-THC), 11-nor-9-Carboxy-Δ9-Tetrahydrocannabinol (COOH-Δ9-THC), 11-nor-9-Carboxy-Δ9-Tetrahydrocannabinol-d9 (COOH-Δ9-THC-d9), 11-Hydroxy-Δ9-Tetrahydrocannabinol (OH-Δ9-THC), Cannabidiol (CBD), 7-Hydroxy-Cannabidiol (7-OH-CBD) and 7-Carboxy-Cannabidiol (7-COOH-CBD) were obtained as 0.1 mg/mL or 1 mg/mL methanolic solutions from Cerilliant via Merck-Sigma, UK.
- Extraction solvent: n-hexane-ethyl acetate (9:1).
- Calibration standards were prepared in the concentration range 0.5 ng – 100 ng/mL in blank plasma. QC samples were prepared at concentrations of 1.5, 6.0 and 20.0 ng/mL in blank plasma.
- Sample preparation: 200 uL of blank, calibration, QC and test samples were spiked with a mixture of deuterium labelled standards and subjected to solvent extractions at neutral and acidic pH and the extracts combined. After evaporation to dryness under nitrogen, samples were derivatized with MTBSTFA and transferred to injection vials for analysis by GC/MS: Agilent 6890 GC/5973 MSD.
- Gas chromatography: carrier gas helium at 1 mL/min; capillary column: Agilent J&W DB-5MSUI 30m x 250µm x 0.25µm; splitless injection at 280°C; temperature program: 150°C to 320°C at 20°C/min and held for 5 min (run time 13.5 min).
- Mass spectrometry: EI 70eV, selected ion monitoring mode: Δ9-THC (m/z 371, 428), Δ9-THC-d3 (m/z 374, 431), OH-Δ9-THC (m/z 413, 501,543), COOH-Δ9-THC-d9 (m/z 524, 422),(COOH-Δ9-THC (m/z 515, 413), CBD (417, 474), CBD-d3 (m/z 417, 474), 7-OH-CBD (m/z 527, 615), 7-OH-CBD-d5 (m/z 532, 426), 7-COOH-CBD (m/z 527,629). Data acquisition control: Agilent Chemstation v. D.03.
- Data processing: Chemstation files were converted and processed using Agilent Masshunter Quantitative Analysis Version B.07.01. Concentrations were calculated by reference to the standard curves obtained.
- Concentrations below lower limit of quantification were replaced with zeros for statistical analyses.

## 1.3 Baseline Variables

**Cannabis use frequency (Screening):** Using an adapted timeline follow-back (TLFB) (6) method, we measured cannabis use frequency in days/week over the previous 4 weeks. We then asked if this pattern of cannabis use was representative of the previous 12 weeks as a whole. Participants were then asked how many days per week they used cannabis on average over the last 12 weeks. Using the TLFB estimate and participants’ answer, participants reported their average cannabis use to 0.5, 1, 2, or 3 days/week over the last 12 weeks.

**Cannabis and other substance use frequency (Baseline):** Cannabis, alcohol, tobacco and illicit drug use frequency (days per week) were all assessed using the TLFB method (6). Participants were prompted to recall the days on which they used these drugs in the 12 weeks prior to baseline. An average use frequency for each drug was calculated in days/week.

**Beck Depression Inventory-II (BDI)** (7). A 21-item self-report questionnaire. Each item is answered with ‘not at all’, ‘mildly’, ‘moderately’ or ‘severely’ and scored from 0 to 3, with total scores ranging from 0 to 63. Higher scores indicate greater levels of depression.

**Beck Anxiety Inventory (BAI)** (8). A 21-item self-report questionnaire. Each item is scored from 0 to 3, with total scores ranging from 0 to 63. Higher scores indicate greater levels of anxiety.

**Short Urgency, Premeditation, Perseverance, Sensation Seeking, Positive Urgency (SUPPS-P).** The SUPPS-P (9) is a 20-item scale assessing multiple facets of impulsivity. Each item is rated on a scale of 1 ‘strongly agree’ to 4 ‘strongly disagree’. The scale is comprised of 5 subscales, each with 4 items: Negative Urgency, Lack of Perseverance, Lack of Premeditation, Sensation Seeking, and Positive Urgency*.* The total score can range from 20 – 80, with higher scores indicating greater impulsivity.

**Socio-economic status (SES):**Assessed using participants’ mother’s educational level. Participants are categorized into those whose mothers have an educational level of undergraduate degree or above, and those whose mothers have an educational level below undergraduate degree.

**Premorbid verbal intelligence – Wechsler Test of Adult Reading (WTAR)**

Premorbid verbal intelligence was assessed using the Wechsler test of adult reading (WTAR) (10). Participants read a list of 50 words out loud and were marked on correct pronunciation. Scores were age adjusted scores and standardized to a normal IQ scale, with mean 100. Scores below 85 were replaced with the value 85.

**Alcohol Use Disorder Identification Test (AUDIT):**A 10-item questionnaire assessing hazardous drinking and dependence. Scores range from 0-40 (11).

**Cannabis Use Disorder Identification Test- Revised (CUDIT-R):**An 8-item questionnaire assessing hazardous cannabis use and dependence. Scores range from 0-32 (12).

**Diagnostic and Statistical Manual of Mental Disorders Fifth Edition (DSM-5) Cannabis Use Disorder (CUD)** (American Psychiatric Association, 2013)**using the Mini International**

**Neuropsychiatric Interview (MINI)** (13)

Severity of CUD was assessed using the MINI, in which 11 DSM-5 symptoms are assessed over the last 12 months. The presence of 0-1 symptoms is considered ‘none’, 2-3 symptoms is considered ‘mild’, 4-5 symptoms is considered ‘moderate’, and 6 or more symptoms is considered ‘severe’. We categorised users into having ‘severe CUD’, or ‘not severe CUD’.

**Cannabis type**

Participants were asked which type of cannabis they most frequently used out of the following categories: strong herbal cannabis (i.e. skunk, seedless, sinsemilla, indoor-grown, high-potency herbal), weak (i.e. seeded herbal cannabis, outdoor grown cannabis, sometimes called ‘Thai’ or ‘brickweed’), hash (i.e. resin, brown solid made from compressed resin glands), or other (e.g. THC oil). We used pictures of the former three categories to aid participants identify which cannabis type they most often used.

## 1.4 Primary & Secondary Outcomes

**Subjective effect visual analogue scales (VAS)**

Participants completed seven subjective effects visual analogue scales (VAS), each of which was a single scale probing a unique subjective effect. These included: ‘feel drug effect’, ‘like drug effect’, ‘dislike drug effect’, ‘anxious’, ‘paranoid’, ‘want cannabis’, and ‘happy’. Participants were asked to rate how strongly they felt each effect on a numeric scale of 0 ‘not at all’ to 10 ‘extremely’, as exemplified in Figure S2. The three drug effects (‘feel drug effect’, ‘like drug effect’, and ‘dislike drug effect’) were completed at T2 – T5, while the remaining four were completed at T1 – T5. Relative to the start of drug inhalation (T1) the time-points correspond as following: T1 = -5 min, T2 = +20 min, T3 = +30 min, T4 = +120 min, and T5 = +160 min.

*For each item, please circle the number that describes how you feel* ***RIGHT NOW.***

**Feel drug effect**

| Not at all | 0 | 1 | 2 | 3 | 4 | 5 | 6 | 7 | 8 | 9 | 10 | Extremely |
| --- | --- | --- | --- | --- | --- | --- | --- | --- | --- | --- | --- | --- |

**Figure S2**. Example of Subjective Effects visual analogue scale (VAS) for a single response variable.

**Psychotomimetic States Inventory**

The PSI (14), a 48-item self-report questionnaire assessing psychotic-like symptoms. Participants were given questions and asked to rate how their experiences ‘right now’. Each item is answered with ‘not at all’, ‘slightly’, ‘moderately’ or ‘strongly’ and scored from 0-3. Total scores range from 0 to 144. Higher scores indicate greater psychotic-like symptomatology. The PSI is comprised of 6 subscales; cognitive disorganisation (9 items, scores range from 0 to 27), perceptual distortion (10 items, scores range from 0 to 30), anhedonia (7 items, scores range from 0 to 21), paranoia (8 items, scores range from 0 to 24), mania (6 items, scores range from 0 to 18), delusory thinking (8 items, scores range from 0 to 24).

**Prose Recall Task**

Verbal episodic memory (VEM) was assessed using the prose recall task from the Rivermead Behavioural Memory Test battery (15). Participants were played a 30 second story via headphones 20 minutes after starting cannabis inhalation, after which they immediately wrote down what they could remember (i.e. immediate recall). After an approximately 100-minute delay filled with unrelated assessments, participants again wrote down what they could remember from the story (i.e. delayed recall). The story contained 21 ‘idea units’. For each idea unit, one point was given for a word-perfect recall or exact synonym, and half a point was given for a partial recall or close synonym. The maximum score was therefore 21. The primary outcome variable was delayed recall, and the secondary outcome variable was immediate recall.

**Positive and Negative Syndrome Scale (PANSS)**

The PANSS was created to standardise the assessment of the symptoms of schizophrenia (16). We used a version that was adapted in order to assess cannabis-induced psychotic effects (17). We used two of the three PANSS subscales – the positive symptoms scale and the negative symptoms scale, each containing 7 items**.** Each item was scored from 1 (‘absent/does not apply’) to 7 (‘extreme’). Thus, each subscale score was within the range 7 – 49. At the end of each drug-administration session, experimenters administered a clinical interview exploring any psychosis-like effects the participant had experienced that session. Experimenters scored according to the participant’s responses and experimenters’ observations.

**Time since last cannabis use.**

A TLFB was completed during each session, in which participants reported whether they had used cannabis. During the baseline session the TLFB extended back 28 days. During drug administration sessions, the TLFB extended back until the day of the previous session (either baseline or drug administration session). In cases where participants had used cannabis since their last session, and within the past 7 days, the time of last use was recorded, and ‘time since last cannabis use’ was calculated in hours. In cases where participants had used cannabis since their last session, but not within the last 7 days, the ‘time since last cannabis use’ was calculated to the nearest day as reported on the TLFB. In cases where the last instance of cannabis use was during a drug administration session (i.e. ‘THC’ or ‘THC+CBD’) then hours since last cannabis use was calculated to their last session.

**Time to inhale contents of balloon.**

Researchers used a stopwatch and timed from the first inhale of each cannabis vapour filled balloon until the balloon was empty. Total time to inhale = time to finish inhaling the first balloon (minutes) + time to finish inhaling the second balloon.

**Acute Coughing Rating.** Researchers rated participants’ coughing during drug administration on a VAS of 0 “not at all” – 10 “extremely”. This scale was introduced after several participants had completed all drug sessions.

## 1.5 Statistical Analyses

**Secondary analyses of Primary Outcomes.**

For primary outcome variables (‘feel drug effect’ at T2, PSI total, and prose recall delayed) a series of secondary mixed-ANOVAs with a between-subject factor of age-group (adolescent, adult), and within-subject factors of drug (‘PLA’, ‘THC’, ‘THC+CBD’) were conducted as listed below (1 – 2). Primary analyses (as detailed in main text) were conducted using imputed data, results from which are reported in full. Results of the secondary analyses (1-2 below) were only reported if there were any discrepancies with results from primary analyses.

1. Secondary analysis: Mixed-ANOVA with imputed data, with drug order as a covariate.
2. Secondary analysis: Mixed ANOVA with imputed data, where participants with unexpected subjective effects (n=1) or plasma THC levels (n=1) were excluded.

**Analyses of secondary outcomes**

Two-way and three-way mixed ANOVAs were conducted to analyse the secondary outcomes detailed below. Where significant main effects or interactions were found, post-hoc pairwise Bonferroni-corrected t-tests were conducted to explore them. Two-way mixed ANOVAs with a between-subject factor of age-group (adolescent, adult), and within-subject factors of drug (‘PLA’, ‘THC’, ‘THC+CBD’) were conducted for:

1. Prose recall immediate scores
2. PSI subscale scores (delusory thinking, cognitive disorganisation, perceptual distortion, mania, paranoia and anhedonia)
3. PANSS Positive and Negative subscale scores
4. Heart rate at T2
5. Time since last cannabis use (note: non-parametric analyses Mann-Whitney U and Wilcoxon rank sum tests were conducted to test age-group and drug effects due to non-normality of data)
6. Cumulative time to inhale the contents of vapour filled balloons
7. Cough rating during drug administration

A series of three-way mixed ANOVAs, which had an additional within-subjects factor of time were run for VASs (‘feel drug effect’, ‘like drug effect’, ‘dislike drug effect’, ‘anxious’, ‘paranoid’, ‘want cannabis’ and ‘happy’) rated throughout the day (time:  or T2-T5 for the former 3 measures, and T1-T5 for the latter 4) and prose recall (time: immediate and delayed). Area-under-the-curve (AUC) and peak effects were also analysed across the T1/T2-T5 timepoints, with 2x3 mixed ANOVAs.

For one adult on the ‘THC+CBD’ condition, all VASs at T5 were missing. Therefore, analyses were conducted when both imputing these values and leaving them as missing and employing listwise deletion. Any discrepancies in these results are reported.

**PANSS** **– categorical analyses of clinically significant reactions**

These data did not meet assumptions of mixed ANOVA; data were highly skewed and failed tests of normality. As such, generalised equation estimate (GEE) logistic analyses were conducted for PANSS positive subscale. Continuous scores were transformed into a binary outcome of clinically significant psychotic response (1) or not (0), where subscale scores **≥** 10 were considered clinically significant (17,18). Two GEE models were run: one included only main effects of age-group and drug, and the other also included the age-group by drug interaction. For PANSS negative subscale, we could not conduct GEEs because there were zero clinically significant reactions on ‘PLA’. Therefore, we conducted separate chi-square and McNemar’s tests between age-groups and between ‘THC’ and ‘THC+CBD’.

**Plasma THC and CBD levels - mixed effects analyses**

THC and CBD plasma concentrations that were below the lower limit of quantification (LLQ <0.5 ng.ml^-1^) were replaced with zeros for statistical analyses. There were multiple missing datapoints for THC and CBD plasma concentration at T2 (missing during ‘THC+CBD’ n=3, ‘PLA’ n=2). As such, these data were analysed via mixed effects mixed ANOVAs, which can handle missing data. These models included a random intercept, and fixed effects of age-group (time-invariant predictor) and drug (time-varying predictor). This model was also run when excluding the participant with unexpected THC plasma levels (as detailed below in section 2).

**Participant drug guess**

Participant’s guess was transformed into a binary outcome of correct guess (1) and incorrect guess (0) for each drug condition and was analysed via a GEE logistic regression. Two models were run: one contained drug, age-group, and their interaction, the other contained only drug and age-group.

**Exploratory analysis: mixed effects model analysis of THC plasma levels (T2) – impact of inhalation time**

As we identified that participants took significantly longer to complete the inhalation procedure during ‘THC+CBD’ compared to ‘THC’, we explored whether the significant differences in THC plasma levels at T2 during ‘THC+CBD’ vs ‘THC’ was impacted by inhalation time. In particular the impact of inhalation time of the second balloon on THC levels was explored.

Two multilevel linear models (MLM) were run with THC plasma concentration (T2) as the dependent variable to determine whether the time taken to inhale the second balloon accounted for any variation in ‘THC’ and ‘THC+CBD’ THC levels. Model 1 included a random intercept to model between-person variance and a fixed effect of drug (‘THC’ and ‘THC+CBD’). Model 2 also included a fixed effect, time-varying predictor of balloon 2 inhalation time. The models were compared by calculating χ^2^_Change_ from the -2 log-likelihood values. Maximum-likelihood estimation was employed.

# **2. Data Pre-processing and Assumptions**

## 2.1 Missing data imputation

In questionnaires with multiple items, where the number of missing items is <30% total items, missing items were imputed using single-value substitution (19–21) using the overall participant’s mean response for that questionnaire or subscale, rounded to the nearest integer. In instances where data or questionnaires had >30% missing items, these missing datapoints were replaced with the average for that group and drug session, rounded to the nearest integer. Table S2 summarises the number of participants with missing data. In cases where data was fully missing or >30% items were missing, analyses were conducted using both imputed and non-imputed data, and any discrepancies in findings are reported. Where there was >30% missing data/items for a particular outcome for more than one participant (as with THC and CBD plasma concentrations), data were left as missing and assessed via mixed effects analyses.

Assumptions for each analysis were first tested, results from these tests for the primary outcome variables are summarised below.

#### **Table S2.** Number of participants with missing data for all variables (n).

| **Drug** | **Outcome type** | **Variable** | **Adolescent** | **Adult** |
| --- | --- | --- | --- | --- |
| 'THC' | Primary | PSI total | 1 | 2 |
| 'THC' | Secondary | Heart rate (T2) | - | 1 |
| 'THC' | Secondary | PANSS Negative | - | 1 |
| 'THC+CBD' | Primary | PSI total | 4 | 1 |
| 'THC+CBD' | Secondary | Feel drug effect (VAS T5) | - | 1 |
| 'THC+CBD' | Secondary | Like drug effect (VAS T5) | - | 1 |
| 'THC+CBD' | Secondary | Dislike drug effect (VAS T5) | - | 1 |
| 'THC+CBD' | Secondary | Happy (VAS T5) | - | 1 |
| 'THC+CBD' | Secondary | Anxious (VAS T5) | - | 1 |
| 'THC+CBD' | Secondary | Paranoid (VAS T5) | - | 1 |
| 'THC+CBD' | Secondary | Want cannabis (VAS T5) | - | 1 |
| 'THC+CBD' | Secondary | THC plasma concentration (T2) | 2 | 1 |
| 'THC+CBD' | Secondary | CBD plasma concentration (T2) | 2 | 1 |
| 'PLA' | Secondary | THC plasma concentration (T2) | 1 | - |
| 'PLA' | Secondary | CBD plasma concentration (T2) | 1 | - |
| 'PLA' | Primary | PSI total | 1 | - |
| Baseline | n/a | BAI | 1 | - |
| Baseline | n/a | CTQ-25 | - | 1 |
| Baseline | n/a | CUD-MINI | 3 | - |

VAS=visual analogue scale, PSI=psychotomimetic states inventory, PANSS= Positive and negative syndrome scale. CTQ-25=Childhood trauma questionnaire-25, CUD-MINI=Cannabis Use Disorder Mini-International Neuropsychiatric Interview, BAI=Beck Anxiety Inventory, BDI=Beck depression inventory. T2 = 20 min after the start of cannabis inhalation; T5=2 hours 40 min after the start of cannabis inhalation.

#### Table S3. Number of extreme outliers for all outcomes

| **Drug** | **Outcome type** | **Variable** | **Adolescent** | **Adult** |
| --- | --- | --- | --- | --- |
| 'PLA' | Primary | PSI Total | 2 | - |
| 'PLA' | Secondary | THC concentration (T2) | 1 | 1 |
| 'PLA' | Secondary | Dislike drug effect (VAS T2) | 4 | - |
| 'PLA' | Secondary | PANSS Negative | 4 | 1 |
| 'PLA' | Secondary | PANSS Positive | 5 | 4 |
| 'PLA' | Secondary | PSI Delusory thinking | - | 1 |
| 'PLA' | Secondary | PSI Perceptual distortion | - | 1 |
| 'PLA' | Secondary | PSI Cognitive disorganisation | - | 1 |
| 'PLA' | Secondary | PSI Mania | 4 | - |
| 'PLA' | Secondary | PSI Anhedonia | 1 | 3 |
| 'THC' | Secondary | PANSS Negative | 1 | 5 |
| 'THC' | Secondary | Paranoid (VAS T2) | - | 2 |
| 'THC+CBD | Secondary | PSI Anhedonia | - | 2 |
| 'THC+CBD | Secondary | PSI Paranoia | - | 2 |
| 'THC+CBD | Secondary | Like drug effect (VAS T2) | - | 1 |

Questionnaire total/subscale scores or single item data values on each drug session for adolescents and adults. Extreme outlier defined as values outside range of: 3rd quartile + 3 x interquartile range and 1st quartile – 3 x interquartile range. VAS= visual analogue scale. PSI=psychotomimetic states inventory. PANSS= positive and negative syndrome scale. T2 = 20 min after the start of cannabis inhalation; T5= 2 hours 40 min after the start of cannabis inhalation.

## 2.2 Unexpected subjective and plasma results

One male adolescent during ‘THC’ had no THC detected in their plasma at T2 (20 minutes after the start of cannabis inhalation. However, this participant rated ‘feel drug effect’ as 6 during T2 and T3 (30 minutes after the start of cannabis inhalation).

One female adolescent during ‘THC’ rated ‘feel drug effect’ 1 for T2 and T3, however expected levels of THC were detected in plasma their samples.

## 2.3 Testing assumptions to conduct mixed-ANOVA on Primary Outcomes

**‘Feel drug effect’.** There was a full dataset across all drug conditions, with no extreme outliers. As expected, histograms and skewness/kurtosis values for ‘PLA’ in both adolescents and adults indicated non-normality (skewed towards 0), but these were acceptable for data from ‘THC’ and ‘THC+CBD’ conditions. ‘PLA’ residuals were also non-normally distributed. Box’s test of equality of covariance was not significant (assumption met). Mauchly’s test of sphericity was significant, so we applied Greenhouse-Geisser correction. Levene’s tests of equality of variances were non-significant (assumption met). Cook’s distances were acceptable. In line with psychopharmacology experiments with ‘feel drug effect’ outcome, we used parametric statistics, despite skew in the ‘PLA’ condition. This is accepted in psychopharmacology studies (1,22,23). Furthermore, ANOVA is thought to be mainly robust to violations in normality (24,25).

**Prose Recall (delayed)**. There was a full dataset across all drug conditions, with no extreme outliers. Kurtosis and skewness values were around or below 1, and inspected histograms showed a normal distribution. Assumptions were met for Box’s test of equality of covariance and Mauchly’s test of sphericity (these were non-significant). Levene’s tests of equality of variances were non-significant for ‘THC’ and ‘PLA’, but significant for ‘THC+CBD’. Cook’s distances were acceptable.

**PSI Total.** There was a full dataset across all drug conditions. There were 2 extreme outliers for adolescents during ‘PLA’. Histograms and skewness/kurtosis values for ‘PLA’ in both adolescents and adults indicated non-normality, but these were acceptable for data in the ‘THC’ and ‘THC+CBD’ conditions. Box’s test of equality of covariance **was significant (assumption not met).** Mauchly’s test of sphericity was non-significant (assumption met). Levene’s tests of equality of error variances was **significant for ‘PLA’ and ‘THC’, but not ‘THC+CBD’**. Cook’s distances were acceptable. In line with conventions in statistical analyses in psychopharmacology experiments, we used parametric statistics, despite the skew in the ‘PLA’ condition (1,22,23). Furthermore, ANOVA is thought to be mainly robust to violations in normality (24,25).

# **3. Results**

## 3.1 Participant Screening, recruitment and adverse events

In total, 933 potential participants completed the online pre-screening questionnaire, 340 were screened on the telephone, and 70 completed a baseline session (see CONSORT diagram, Figure S3). Twenty-two participants who started or completed the baseline session did not complete the study. Four were deemed ineligible at baseline, for the following reasons: MRI contraindications (n=1), judged unable to tolerate cannabis dose (n=2), and too frequent use of other illicit drugs (n=1). Seven dropped out between the baseline session and the first drug administration session: three due to COVID-19 lockdowns, four due to other reasons. Nine dropped out between the first and second session: two due to adverse reactions after drug administration, three due to COVID-19 related restrictions and four for other reasons. Two dropped out between the second and third sessions, for the following reasons: one following an adverse drug reaction and one for COVID-19 related restrictions. The participants who dropped-out not significantly different to our included participants. Table S7 summarises the baseline sociodemographic characteristics and drug use of the 11 participants who dropped out after at least one drug administration session, and compares it with those who were included. As planned, only participants who completed the whole study (all three drug administration sessions) were analysed (n=48; 24 adolescents and 24 adults).


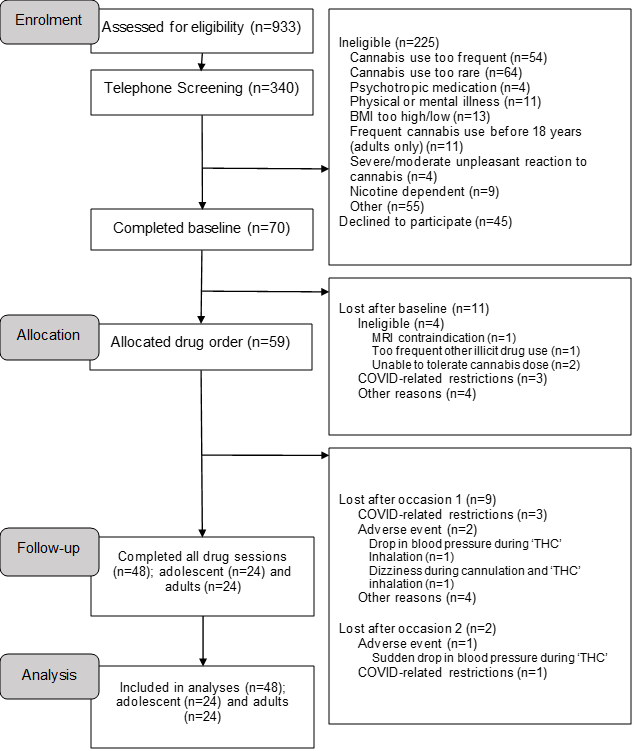


**Figure S3:** Consolidated Standards of Reporting Trials (CONSORT) flowchart for all participants. Participants’ eligibility was first assessed online and subsequently on the telephone, when we recorded reasons for ineligibility. Participants’ eligibility was further checked at an in-person or video-call baseline session. Participants who passed screening and baseline sessions were randomly allocated a drug order. We continued the experiment until 48 participants had completed all three drug administration sessions, in a per-protocol fashion. Participants who dropped out after one or two drug administration sessions were excluded. Other reasons for dropping out include scheduling conflicts, personal reasons, and no reason given. COVID-related restrictions were primarily due to government restrictions in March 2020 (after which the study was paused for seven months) and January 2021.

All drug-related withdrawals occurred on the ‘THC’ condition: one adult experienced a sudden drop in blood pressure during drug inhalation (on occasion 2), one adolescent experienced dizziness following both cannula insertion and at the start of drug inhalation, and one adolescent felt faint during the session and nauseous afterwards (on occasion 1). Of the participants who completed all three sessions (n=48), four adolescents experienced an adverse event of light headedness or faintness during or following cannabis administration, all of which were during ‘THC+CBD’.

## 3.2 Baseline characteristics, cannabis, and other drug use variables

For participant baseline characteristics with age-group comparisons see Table S4, with participants’ cannabis use information in Table S5. Within both the adolescent (n=24) and adult (n=24) group, 50% of participants were female. Participants’ cannabis use frequency (days/week) at baseline were closely matched between age-groups (adolescents: mean=1.41, SD=0.77; adults mean=1.45, SD=0.77, t_46_ = -0.187, p=0.852). As expected, adults first used cannabis at a later age than adolescents, had a greater time between first use and current age (i.e. cannabis use ‘duration’) and had a greater number of lifetime days of cannabis use, while adolescents had greater CUDIT-R scores (Table S5). Moreover, there were no significant differences between adolescent and adult use frequency of other illicit drugs or cigarettes (t_46_= 0.509, p=0.613; t_46_=1.89, p=0.065 respectively).

Adults used alcohol more frequently (M=2.16 days per week, SD=1.7) than adolescents’ (M=0.56, SD=0.62; t_46_ =-4.306, p<0.001), however AUDIT scores did not differ significantly across age group (t_46_=-1.268, p=0.211). Adolescents scored higher than adults on measures of cannabis use disorder symptoms (CUDIT-R; t_46_=3.175, p=0.003, Cohen’s d (d)=1.05), impulsivity (S-UPPS-P; t_46_=2.284, p=0.027, d=0.68), depression (BDI; t_46_= 2.325, p=0.025, d=0.70), and anxiety (BAI; t_46_=2.273, p=0.028, d=0.69).

#### Table S4. Baseline and demographic data**.** Either n (%) or mean (SD) [min-max].

|  | | |  | | **Adolescents (n=24)** | | | | **Adults (n=24)** | | **Both (n=48)** | | **Differences** | | | **Test statistic** | | | | | | |  |  |  |  |  |  |
| --- | --- | --- | --- | --- | --- | --- | --- | --- | --- | --- | --- | --- | --- | --- | --- | --- | --- | --- | --- | --- | --- | --- | --- | --- | --- | --- | --- | --- |
| **Gender** | | | Female | | 12 (50.0%) | | | | 12 (50.0%) | | 24 (50%) | |  | | | p=1 | | | | | | |  |  |  |  |  |  |
|  | | | Male | | 12 (50.0%) | | | | 12 (50.0%) | | 24 (50%) | | ns | | |  | | | | | | |  |  |  |  |  |  |
| **Age (years)** | | |  | | 17.17 (0.43) | | | | 27.77 (1.04) [26.33-29.58] | | 22.47 (5.41) [16.50-29.58] | | Adult> | | | t(46)=46.101, p<0.001 | | | | | | |  |  |  |  |  |  |
|  |  |  |  |  | [16.50-17.92] | | | |  |  |  |  | Adolescent*** | | |  |  |  |  |  |  |  |  |  |  |  |  |  |
| **Weight (kg)** | | |  | | 63.19 (8.51) [51.40-80.10] | | | | 69.18 (12.99) [51.00-99.90] | | 66.18 (11.28) [51.00-99.90] | | ns | | | t(46)=1.888, p=0.065 | | | | | | |  |  |  |  |  |  |
| **Height (m)** | | |  | | 1.70 (0.11) [1.50-1.86] | | | | 1.72 (0.11) [1.58-1.92] | | 1.71 (0.11) [1.50-1.92] | | ns | | | t(46)=0.632, p=0.531 | | | | | | |  |  |  |  |  |  |
| **BMI (kg/m^2^)** | | |  | | 21.89 (2.81) [17.36-26.67] | | | | 23.18 (2.73) [19.40-27.80] | | 22.53 (2.82) [17.36-27.80] | | ns | | | t(46)=1.613, p=0.114 | | | | | | |  |  |  |  |  |  |
| **BMI percentile (%)** | | |  | | 56.71 (29.21) [2.00-95.00] | | | | NA | |  | |  | | |  | | | | | | |  |  |  |  |  |  |
| **Ethnicity** | | | White | | 17 (70.8%) | | | | 18 (75.0%) | | 35 (72.9%) | | ns | | | χ^2^(1)=0.105, p=0.745 | | | | | | |  |  |  |  |  |  |
|  | | | Mixed | | 4 (16.7%) | | | | 1 (4.2%) | | 5 (10.4%) | |  | | |  | | | | | | |  |  |  |  |  |  |
|  | | | Asian | | 1 (4.2%) | | | | 3 (12.5%) | | 4 (8.3%) | |  | | |  | | | | | | |  |  |  |  |  |  |
|  | | | Black | | 0 (0.0%) | | | | 2 (8.3%) | | 2 (4.2%) | |  | | |  | | | | | | |  |  |  |  |  |  |
|  | | | Other | | 1 (4.2%) | | | | 0 (0.0%) | | 1 (2.1%) | |  | | |  | | | | | | |  |  |  |  |  |  |
|  | | | Prefer not to say | | 1 (4.2%) | | | | 0 (0.0%) | | 1 (2.1%) | |  | | |  | | | | | | |  |  |  |  |  |  |
| **Adjusted WTAR Score** | | | | | 111.25 (11.71) [85.00-122.00] | | | | 117.83 (6.12) [106.00-127.00] | | 114.4 (10.31) [78.00-127.00] | | Adult> | | t(46)=2.441, | | | | | |  |  |  |  |  |  |  |  |
|  |  |  |  |  |  |  |  |  |  |  |  |  | Adolescent* | | p=0.019 | | | | | |  |  |  |  |  |  |  |  |
| **SES** | Mother’s education below undergraduate degree | | | | 8 (33.3%) | | | | 8 (33.3%) | | 16 (33.3%) | | ns | | | p=1 | | | | | | |  |  |  |  |  |  |
|  | Mother’s education undergraduate degree or above | | | | 16 (66.7%) | | | | 16 (66.7%) | | 32 (66.7%) | | |  | | | | | |  | |  |  |  |  |  |  |  |
| ***Table S4 cont.*** | |  | | | *Adolescents (n=24)* | | | | | *Adults (n=24)* | | *Both (n=48)* | | *Differences* | | | | | | *Test statistic* | | | | | | |  |  |
| **BDI** | |  | | | 10.38 (8.55) [0.00-28.00] | | | | | 5.29 (6.45) [0.00-22.00] | | 7.83 (7.92) [0.00-28.00] | | Adolescent> | | | | | | t(46)=2.325, p=0.025 | | | | | | |  |  |
|  |  |  |  |  |  |  |  |  |  |  |  |  |  | Adult* | | | | | |  | | | | | | | | |
| **BAI** | |  | | | 10.42 (8.15) [0.00-32.00] | | | | | 6.00 (4.92) [0.00-17.00] | | 8.21 (7.02) [0.00-32.00] | | Adolescent> | | | | | | t(46)=2.273, p=0.028 | | | | | | | | |
|  |  |  |  |  |  |  |  |  |  |  |  |  |  | Adult* | | | | | |  |  |  |  |  |  |  |  |  |
| **S-UPPS-P** | |  | | | 48.17 (7.51) [34.00-61.00] | | | | | 42.75 (8.87) [30.00-64.00] | | 45.46 (8.58) [30.00-64.00] | | Adolescent> | | | | | | t(46)=2.284, p=0.027 | | | | | | | | |
|  |  |  |  |  |  |  |  |  |  |  |  |  |  | Adult* | | | | | |  |  |  |  |  |  |  |  |  |
| **AUDIT** | |  | | | 5.88 (5.39) [0.00-21.00] | | | | | 7.71 (4.59)  [1.00-18.00] | | 6.79 (5.04) [0.00-21.00] | | ns | | | | | | t(46)=1.268, p=0.211 | | | | | | | | |
| **Alcohol use frequency (days/week)** | | | | | 0.56 (0.62) [0.00-2.50] | | | | | 2.16 (1.71) [0.00-6.00] | | 1.36 (1.5) [0.00-6.00] | | Adults> | | | | | | t(46)=4.306, | | | | | | | | |
|  |  |  |  |  |  |  |  |  |  |  |  |  |  | Adolescents*** | | | | | | p<0.001 | | | | | | | | |
| **Weekly alcohol use** | | | | No | | 19 (79.2%) | | | | 6 (25.0%) | | 25 (52.1%) | | Adults> | | | | | | χ^2^(1)=14.108, p<0.001 | | | | | | |  |  |
|  |  |  |  |  |  |  |  |  |  |  |  |  |  | Adolescents*** | | | | | |  |  |  |  |  |  |  |  |  |
|  | | | | Yes | | 5 (20.8%) | | | | 18 (75.0%) | | 23 (47.9%) | |  | | | | | |  | | | | | | |  |  |
| **Cigarette/roll-up use frequency (days/week)** | | | | | 1.60 (2.25) [0.00-7.00] | | | | | 0.60 (1.30) [0.00-6.00] | | 1.1 (1.88) [0.00-7.00] | | ns | | | t(46)=1.890, | | | | | | |  |  |  |  |  |
|  |  |  |  |  |  |  |  |  |  |  |  |  |  |  | | | | p=0.065 | | | | | | |  |  |  |  |
| **Weekly cigarette/roll-up use** | | | | No | | | 15 (62.5%) | | | 19 (79.2%) | | 34 (70.8%) | |  | | | | | | χ^2^(1)=1.613, p=0.204 | | | | | | |  |  |
|  |  |  |  | Yes | | | 9 (37.5%) | | | 5 (20.8%) | | 14 (29.2%) | | ns | | | | | |  | | | | | | |  |  |
| **Other illicit drug use frequency (days/week)** | | | | | 0.46 (0.83) [0.00-3.00] | | | | | 0.33 (0.87) [0.00-4.00] | | 0.4 (0.84) [0.00-4.00] | | ns | | | | | t(46)=0.509, p=0.613 | | | | | | |  |  |  |
| **Monthly use other illicit drug** | | | | | No | | | 22 (91.7%) | | 22 (91.7%) | | 44 (91.7%) | | ns | | | P=1 | | | | | | | | | | |  |
|  |  |  |  |  | Yes | | | 2 (8.3%) | | 2 (8.3%) | | 4 (8.3%) | |  | | |  | | | | | | | | | | |  |

BMI= body mass index, SES=socioeconomic status, AUDIT=Alcohol use disorders identification test, BDI=Beck depression inventory, BAI=Beck anxiety inventory, CUDIT-R=Cannabis use disorder identification test-revised, S-UPPS-P= Short Urgency-Premeditation-Perseverance-Sensation Seeking-Positive Urgency, WTAR= Wechsler Test of Adult Reading. Group differences were assessed via independent t-tests or Chi-squared tests. Ethnicity test: white vs non-white. Alcohol, cigarette/roll-up, other illicit drug use data taken from baseline timeline follow-back. *p<0.05, **p<0.01, ***p<0.001.

#### Table S5. Cannabis use information. Either n (%) or mean (SD) [min-max].

|  | |  | | **Adolescents (n=24)** | | **Adults (n=24)** | | **Both (n=48)** | | **Group differences** | | | | **Test statistic** | | | | |  |  |
| --- | --- | --- | --- | --- | --- | --- | --- | --- | --- | --- | --- | --- | --- | --- | --- | --- | --- | --- | --- | --- |
| **Cannabis: use frequency (days/week) - Screening** | |  | | 1.67 (0.89) | | 1.88 (0.97) | | 1.79 (0.92) [0.50-3.00] | |  | | | | t(46)=0.774, | | | | |  |  |
|  |  |  |  | [0.50-3.00] | | [0.50-3.00] | |  |  |  |  |  |  | p=0.443 | | | | |  |  |
| **Cannabis use frequency (days/week) categorical - Screening** | | 0.5 | | 4 (16.7%) | | 4 (16.7%) | | 8 (16.7%) | |  | | | | χ^2^(3)=1.092, | | | | |  |  |
|  |  |  |  |  |  |  |  |  |  |  |  |  |  | p=0.779 | | | | |  |  |
|  |  | 1 | | 7 (29.2%) | | 5 (20.8%) | | 12 (25.0%) | |  | | | |  | | | | |  |  |
|  | | 2 | | 8 (33.3%) | | 7 (29.2%) | | 15 (31.3%) | |  | | | |  | | | | |  |  |
|  | | 3 | | 5 (20.8%) | | 8 (33.3%) | | 13 (27.1%) | |  | | | |  | | | | |  |  |
| **Cannabis use frequency (days/week) – Baseline TLFB** | |  | | 1.41 (0.77) | | 1.45 (0.77) | | 1.43 (0.77) [0.25-3.50] | |  | | | | t(46)=0.592, | | | | |  |  |
|  |  |  |  | [0.25-3.50] | | [0.25-2.75] | |  |  |  |  |  |  | p=0.852 | | | | |  |  |
| **Cannabis: amount used on a day of use (grams)** | |  | | 0.81 (0.56) | | 0.50 (0.52) [0.10-2.00] {n=23} | | 0.66 (0.55) [0.10-2.50] | |  | | | | t(45)=1.986, | | | | |  |  |
|  |  |  |  | [0.25-2.50] | |  |  |  |  |  |  |  |  | p=0.053 | | | | |  |  |
| **Cannabis: age of first use (years)** | |  | | 14.55 (1.03) [11.92-16.08] | | 18.17 (2.62) [14.00-24.42] | | 16.36 (2.69) [11.92-24.42] | | Adults> | | | | t(46)=6.317, | | | | |  |  |
|  |  |  |  |  |  |  |  |  |  | Adolescents*** | | | | p<0.001 | | | | |  |  |
| **Cannabis: time since first use (years)** | |  | | 2.63 (1.06) [0.75-5.42] | | 9.60 (2.37) [4.17-12.83] | | 6.11 (3.97) [0.75-12.83] | | Adults> Adolescents** | | | | t(46)=13.130,  p=0.001 | | | | |  |  |
| ***Table S5 cont.*** |  | | | | **Adolescents (n=24)** | | **Adults (n=24)** | | **Both (n=48)** | | **Group differences** | **Test statistic** | | | |  |  |  |  |  |
| **Cannabis: lifetime days of use** |  | | | | 153.67 (89.97) [11.00-418.00] | | 544.29 (630.94) [136.00-3127.00] | | 349.13 (487.63) [11.00-3127.00] | |  | | t(46)=3.008, | | | | | |  |  |
|  |  |  |  |  |  |  |  |  |  |  | Adults>Adolescents** | | p=0.004 | | | | | |  |  |
|  |  |  |  |  |  |  |  |  |  |  |  | |  | | | | | |  |  |
| **CUDIT-R** |  | | | | 10.17 (3.14) [5.00-16.00] | | 7.21 (3.31) | | 8.69 (3.53) [3.00-16.00] | | Adolescent> | | t(46)=3.175, | | | | | | |  |
|  |  |  |  |  |  |  | [3.00-15.00] | |  |  | Adult** | | p=0.003 | | | | | | |  |
| **Total number of DSM CUD symptoms** |  | | | | 1.42 (1.69) | | 0.92 (1.35) | | 1.17 (1.53) [0.00-6.00] | |  | | t(46)=1.132, | | | | | | |  |
|  |  |  |  |  | [0.00-6.00] | | [0.00-4.00] | |  |  |  |  | p=0.263 | | | | | | |  |
| **CUD category** | |  | None | | 14 (58.3%) | | 18 (75.0%) | | 32 (66.7%) | |  | | χ^2^(3)=2.318, | | | | | | |  |
|  |  |  |  |  |  |  |  |  |  |  |  |  | p=0.509 | | | | | | |  |
|  |  |  | Mild | | 7 (29.2%) | | 4 (16.7%) | | 11 (22.9%) | |  | |  | | | | | | |  |
|  |  |  | Moderate | | 2 (8.3%) | | 2 (8.3%) | | 4 (8.3%) | |  | |  | | | | | | |  |
|  |  |  | Severe | | 1 (4.2%) | | 0 (0.0%) | | 1 (2.1%) | |  | | | |  | | |  |  |  |
|  | |  | Strong | | 18 (75.0%) | | 19 (79.2%) | | 37 (77.1%) | |  | | χ^2^(2)=0.360, p=0.835 | | | |  |  |  |  |
| **Cannabis type most frequently used** | |  | Weak | | 4 (16.7%) | | 4 (16.7%) | | 8 (16.7%) | |  | |  | | | |  |  |  |  |
|  |  |  | Hash | | 2 (8.3%) | | 1 (4.2%) | | 3 (6.3%) | |  | |  | | | |  |  |  |  |

CUD= cannabis use disorder, DSM= diagnostic and Statistical Manual of Mental Disorders, 5th Edition, TLFB= timeline follow back. CUDIT-R=Revised Cannabis Use Disorder Identification Test. *p<0.05, **p<0.01, ***p<0.001.

#### Table S6. Lifetime drug use variables. N (%).

|  | **Adolescents (n=24)** | **Adults (n=24)** | **Both (n=48)** |
| --- | --- | --- | --- |
| Alcohol: ever used | 24 (100.0%) | 24 (100.0%) | 48 (100.0%) |
| Alcohol: used in past 12 weeks | 20 (83.3%) | 24 (100.0%) | 44 (91.7%) |
| Cigarettes: ever used | 21 (87.5%) | 23 (95.8%) | 44 (91.7%) |
| Cigarettes: used in past 12 weeks | 19 (79.2%) | 12 (50.0%) | 31 (64.6%) |
| Cocaine: ever used | 4 (16.7%) | 23 (95.8%) | 27 (56.3%) |
| Cocaine: used in past 12 weeks | 0 (0.0%) | 6 (25.0%) | 6 (12.5%) |
| Ecstasy: ever used | 15 (62.5%) | 24 (100.0%) | 39 (81.3%) |
| Ecstasy: used in past 12 weeks | 8 (33.3%) | 3 (12.5%) | 11 (22.9%) |
| Synthetic cannabinoids: ever used | 4 (16.7%) | 3 (12.5%) | 7 (14.6%) |
| Synthetic cannabinoids: used in past 12 weeks | 0 (0.0%) | 0 (0.0%) | 0 (0.0%) |
| Ketamine: ever used | 16 (66.7%) | 11 (45.8%) | 27 (56.3%) |
| Ketamine: used in past 12 weeks | 8 (33.3%) | 2 (8.3%) | 10 (20.8%) |
| Magic mushrooms: ever used | 4 (16.7%) | 20 (83.3%) | 24 (51.1%) |
| Magic mushrooms: used in past 12 weeks | 1 (4.2%) | 3 (12.5%) | 4 (8.3%) |
| LSD: ever used | 10 (41.7%) | 14 (58.3%) | 24 (50.0%) |
| LSD: used in past 12 weeks | 6 (25.0%) | 0 (0.0%) | 6 (12.5%) |
| Laughing gas: ever used | 18 (75.0%) | 19 (79.2%) | 37 (77.1%) |
| Laughing gas: used in past 12 weeks | 5 (20.8%) | 2 (8.3%) | 7 (14.6%) |
| Amphetamine: ever used | 2 (8.3%) | 11 (45.8%) | 13 (27.1%) |
| Amphetamine: used in past 12 weeks | 1 (4.2%) | 2 (8.3%) | 3 (6.3%) |
| Methamphetamine: ever used | 0 (0.0%) | 1 (4.2%) | 1 (2.1%) |
| Methamphetamine: used in past 12 weeks | 0 (0.0%) | 0 (0.0%) | 0 (0.0%) |
| Crack cocaine: ever used | 0 (0.0%) | 1 (4.2%) | 1 (2.1%) |
| Crack cocaine: used in past 12 weeks | 0 (0.0%) | 0 (0.0%) | 0 (0.0%) |
| Heroin: ever used | 0 (0.0%) | 0 (0.0%) | 0 (0.0%) |
| Heroin: used in past 12 weeks | 0 (0.0%) | 0 (0.0%) | 0 (0.0%) |
| Alprazolam ‘Xanax’: ever used | 8 (33.3%) | 5 (20.8%) | 13 (27.1%) |
| Alprazolam ‘Xanax’: used in past 12 weeks | 2 (8.3%) | 2 (8.3%) | 4 (8.3%) |
| Diazepam ‘Valium’: ever used | 0 (0.0%) | 8 (33.3%) | 8 (17.0%) |
| Diazepam ‘Valium’: used in past 12 weeks | 0 (0.0%) | 1 (4.2%) | 1 (2.1%) |

#### Table S7. Sociodemographic and cannabis use information of participants who dropped-out, compared with participants who were included. Either n (%) or mean (SD).

|  |  | Excluded |  | Included |  |  |
| --- | --- | --- | --- | --- | --- | --- |
|  |  | **Adolescent (n=5)** | **Adult (n=6)** | **Adolescent (n=24)** | **Adult (n=24)** | Comparison |
| Gender | Female | 2 (40%) | 2 (33%) | 12 (50%) | 12 (50%) | ns |
|  | Male | 3 (60%) | 4 (67%) | 12 (50%) | 12 (50%) |  |
| Age (years) |  | 17.66 (0.40) | 28.27 (1.48) | 17.17 (0.43) | 27.77 (1.04) | ns |
| Cannabis use frequency (days/week) |  | 1.00 (0.40) | 1.04 (0.80) | 1.41 (0.77) | 1.45 (0.77) | ns |

## 3.3 Time since last cannabis use

Greenhouse-Geisser correction was applied. A mixed-ANOVA with within subjects factor of drug (‘THC’, ‘THC+CBD’, and ‘PLA’), and between subjects factor of age-group (adolescents and adults) revealed no main effect of age-group (F(2,96)=0.000, p=0.996, η^2^_p_ =0.000) or drug (F(2,96)=2.592, p=0.101, η^2^_p_ =0.053) and no interaction between the two (F(2,96)=1.178, p=0.299, η^2^_p_ =0.025), for time since last cannabis use. The pattern of results found in mixed-ANOVA was somewhat supported via non-parametric analyses. Mann-Whitney U tests also showed there were no significant time differences in between adolescents and adults across all drug conditions: PLA (Z=-0.052, p=0.959), THC (Z=-0.722, p=0.470), and THC+CBD (Z=-0.897, p=0.370).  Wilcoxon rank sum test indicated a significant lower ‘time since last cannabis use’ during placebo condition vs ‘THC’ and ‘THC+CBD’ (‘THC’ vs ‘PLA’ Z=-3.016, p=0.003; THC+CBD v PLA: Z=-2.994, p=0.003) with no difference between ‘THC’ and ‘THC+CBD’ (Z=-1.355, p=0.175). Finally, there was no significant difference between the last time adolescents and adults used cannabis before the baseline session (t(46)=1.544, MD=58.875, p=0.134).

#### Table S8. Time since last cannabis use (hours) before each session. Mean (SD) [min – max].

|  | **Adolescent (n=24)** | **Adult (n=24)** | **Both (n=48)** |
| --- | --- | --- | --- |
| Baseline | 187.29 (176.12) [28.00-720.00] | 128.42 (62.45) [40.00 -288.00] | 157.85 (134.06) [28.00 – 720.00] |
| 'PLA' | 144.99 (193.34) [69.50-1032.00]* | 124.00 (81.52) [72.00-456.00] | 134.49 (147.16) [69.501032.00] |
| 'THC' | 192.17 (233.16) [72.00-1224.00] | 174.69 (102.31) [80.00-432.00] | 183.43 (178.34) [72.00-1224.00] |
| 'THC+CBD' | 131.03 (59.55) [74.33-288.00] | 169.99 (126.88) [69.00-624.00] **ǂ** | 150.51 (100.00) [69.00-624.00] |

***ǂ** One participant during ‘PLA’ and one participant during ‘THC+CBD’ used cannabis <72 hours prior to a drug administration session, in breach of abstinence rules. However, as the participants were unable to reschedule their session, experimenters made the decision to continue with the session considering the abstinence requirement was violated to a minor degree (i.e. only by 3 hours).

## 3.4 Primary Outcomes

#### Table S9. Descriptive statistics of primary outcomes

| **Outcome** | **Condition** | **ADOLESCENTS** | **ADULTS** | **BOTH** |
| --- | --- | --- | --- | --- |
| Feel Drug Effect (T2) | **‘PLA’** | 0.958 (1.546) [0-6] | 1.625 (1.952) [0-6] | 1.291 (1.774) [0-6] |
|  | **‘THC’** | 6.958 (2.156) [1-10] | 8.208 (1.444) [5-10] | 7.583 (1.922) [1-10] |
|  | **‘THC+CBD’** | 7.792 (1.560) [5-10] | 8.417 (1.213) [6-10] | 8.104 (1.418) [5-10] |
| PSI Total | **‘PLA’** | 16.416 (15.068) [1-55] | 10.875 (7.279) [2-35] | 13.646 (12.036) [1-55] |
|  | **‘THC’** | 23.458 (15.231) [4-61] | 19.375 (9.011) [2-37] | 21.417 (12.551) [2-61] |
|  | **‘THC+CBD’** | 24.792 (12.601) [4-55] | 24.083 (10.219) [5-45] | 24.438 (11.355) [4-55] |
| Delayed prose recall | **‘PLA’** | 8.375 (4.150) [3.5-18.5] | 9.291 (3.193) [3-14] | 8.833 (3.692) [3-18] |
|  | **‘THC’** | 6.125 (3.129) [1.5-14.5] | 6.063 (3.405) [1-11.5] | 6.094 (3.235) [1-14] |
|  | **‘THC+CBD’** | 6.042 (3.793) [1-12] | 5.833 (2.721) [1-11] | 5.938 (3.268) [1-12] |

‘Feel drug effect’ at T2, PSI total, and prose recall (delayed)) during each drug administration session for adolescents (n=24) and adults (n=24) and both combined (n=48). Mean (SD) [min-max]. PSI= psychotomimetic states inventory. Relative to the start of drug administration (time-inh) these were assessed as following: ‘feel drug effect’ at T2 = +20 min; prose recall (delayed) at +120 min (which was after a 100 min delay since hearing the story); PSI at +130 min.

### 3.4.1 Results from secondary analyses of primary outcomes.

For ‘feel drug effect’ at T2, prose recall delayed and PSI total results from primary analyses did not change significantly when conducting the following secondary analyses:

- Mixed-ANOVA with imputed data, with drug order as a covariate: no changes.
- Mixed ANOVA with imputed data, where participants with unexpected subjective* effects (n=1) or plasma THC levels (n=1) were excluded (either or both excluded): no changes.

*See section 2 for details.

## 3.5 Secondary outcome results

### 3.5.1 Subjective Effects-VAS over time.

**Feel drug effect (Figure S4a-c; Table S10a).** There was a main effect of drug (F(2,92)=284.486, p<0.001, η^2^_p_ =0.861) and time (F(3, 138)=323.986, p<0.001, η^2^_p_ =0.876), and a significant drug by time interaction (F(6, 276)=61.846, p<0.001, η^2^_p_=0.573). ‘Feel drug effect’ ratings were greater during ‘THC’ and ‘THC+CBD’ relative to ‘PLA’ (t(47)=16.179, p<0.001, MD=4.552 and t(47)=21.586, p<0.001, MD=5.12, respectively). Additionally, in contrast with the results from 2x3 ANOVA for ‘feel drug’ at T2, ratings were greater during ‘THC+CBD’ relative to ‘THC’ (t(47)=3.240, p=0.007, MD=0.568).

The drug by time interaction was driven by greater reductions in ‘feel drug effect’ for ‘THC’ and ‘THC+CBD’ than in ‘PLA’. The fall in average rating from T2 to T5 was very large for ‘THC’ (t(47)=18.470, p<0.001, MD=5.563) and ‘THC+CBD’ (t(47)=18.614, p<0.001, MD=5.458), and considerably smaller for ‘PLA’ (t(47)=5.042, p<0.001, MD=1.125).

**Secondary analysis**: When analysing non-imputed data there was a main effect of age-group (F(1,25), p=0.035, η^2^_p_=0.095), this was driven by adults rating higher than adolescents (t(46)=2.179, p=0.035, MD=0.656).

**Like Drug effect (Figure S4d-f; Table S10b).** There was a main effect of drug (F(2,92)=48.811, p<0.001, η^2^_p_=0.515); time (F(3,138)=34.495, p<0.001, η^2^_p_=0.429); and a significant interaction between drug and time (F(6,276)=6.432, p<0.001, η^2^_p_ =0.123) for ‘like drug’. Ratings of ‘like drug’ were higher during ‘THC’ and ‘THC+CBD’ relative to ‘PLA’ (t(47)=7.860, p<0.001, MD=3.589 and t(47)=6.914, p<0.001, MD=3.536, respectively), while ratings were similar for ‘THC’ and ‘THC+CBD’ conditions.

Across age-group and drug, ratings of ‘like drug’ decreased over time from T2 to T5 (t(47)=7.280, p<0.001, MD=1.722). The interaction between drug and time was driven by a greater reduction in ratings from T3 to T5 for ‘THC’ (t(47)=6.66, p<0.001, MD=2.458) and ‘THC+CBD’ (t(47)=4.953, p<0.001, MD=1.813), relative to a gradual reduction over time for ‘PLA’ (t(47)=1.926, p=0.362, MD=0.417).

**Secondary analysis:** This pattern of results was almost identical whether missing data were imputed or not.

**Dislike drug effect (Figure S4g-i; Table S10c).** There was a main effect of time (F(3,138)=11.416, p<0.001, η^2^_p_ =0.019) and significant interaction between age and time (F(3,138)=3.287, p=0.023, η^2^_p_=0.067). This interaction was driven by a greater decrease in ratings of ‘dislike drug’ for adults over time (T2 to T5: t(47)=5.07, MD=1.278, p<0.001) relative to adolescent’s ratings which did not significantly differ over time (T2 to T5: t(47)=2.039, MD=0.514, p=0.283).

There was a significant interaction between drug and time (F(6,276)=4.35, p=0.001, η^2^_p_=0.086). This interaction between drug and time was driven by significant decreases in ‘dislike drug’ ratings from T2 to T5 during ‘THC’ and ‘THC+CBD’ (t(47)=4.274, p=0.001, MD=1.167, and t(47)=3.968, p=0.001, MD=1.417, respectively) relative to no significant changes in rating during ‘PLA’ (t(47)=0.506, p=1, MD=0.104).

**Secondary analysis:** This pattern of results was almost identical whether missing data were imputed or not.


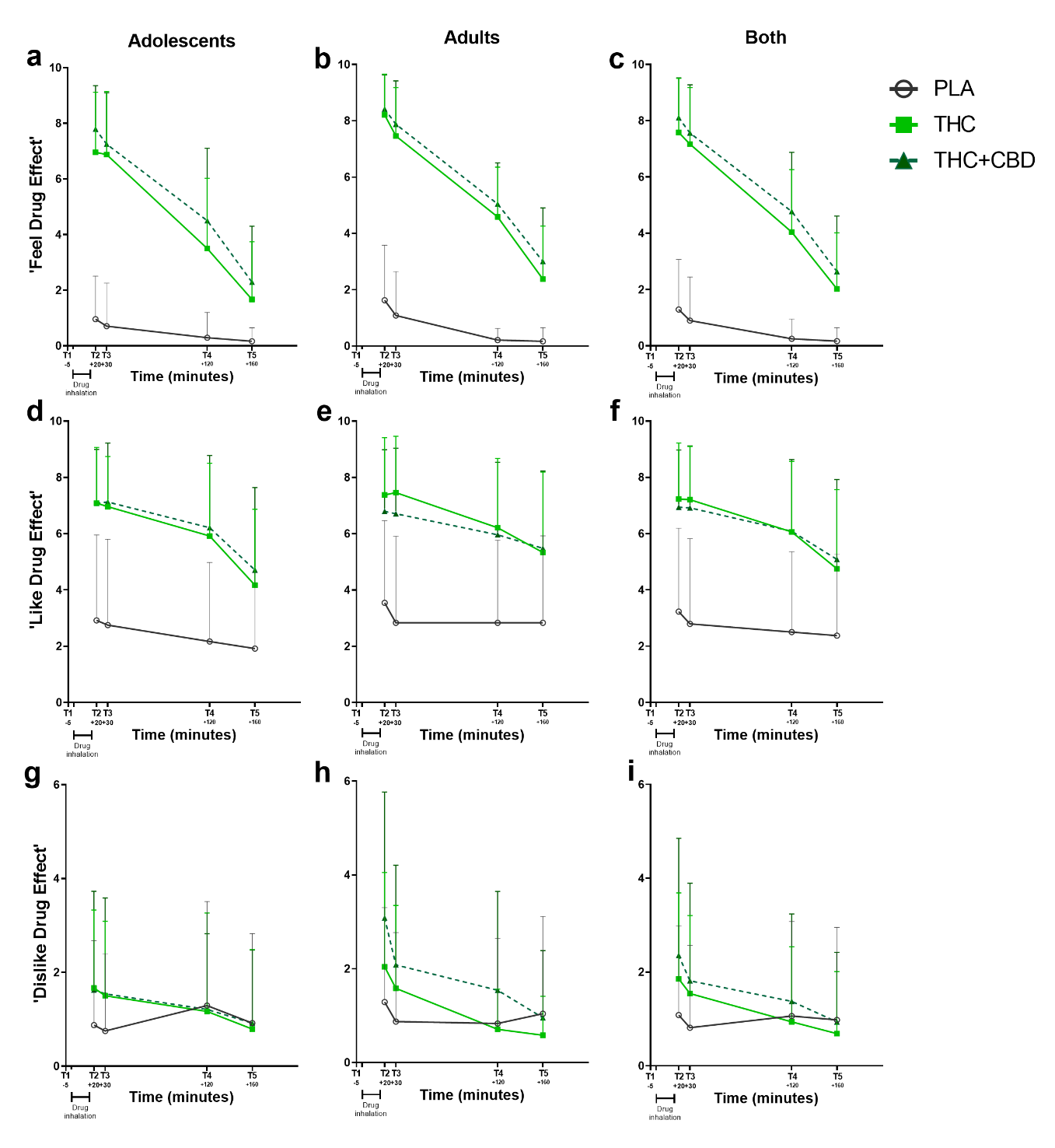


**Figure S4.** Mean values for subjective effect VASs (‘feel drug effect’, ‘like drug effect’, and ‘dislike drug effect’) with 95% confidence intervals over time for adolescents and adults (n=24 per group for full datasets) during ‘PLA’, ‘THC’ and ‘THC+CBD’ conditions. VASs ranged from 0=‘not at all’ to 10=‘extremely’. Data from the two age-groups has been combined to show overall drug effects in c, f and i (n=48). Relative to the start of drug inhalation (time-inh) the time-points correspond as following: T2 = +20 min, T3 = +30 min, T4 = +120 min, and T5 = +160 min. Subjective effect VASs was not collected before cannabis inhalation (-5mins). VAS=visual analogue scale. Data is missing for 1 adult during T5 of ‘THC+CBD’.

**Anxious (Figure S5a-c, Table S10d)**. Greenhouse-Geisser correction was applied to time and drug by time. There was a trend interaction between age-group and drug (F(2,92)=2.868, p=0.062, η^2^_p_=0.059). There were main effects of drug (F(2,92)=15.603, p<0.001, η^2^_p_=0.253), time (F(4,184)=42.463, p<0.001, η^2^_p_=0.48), and a significant interaction between the two (F(8,368)=6.514, p<0.001, η^2^_p_=0.124). Ratings of ‘anxious’ were greater during ‘THC’ and ‘THC+CBD’ relative to ‘PLA’ (t(92)=4.247, p<0.001, MD=0.792 and t(92)=5.398, p<0.001, MD=0.979, respectively), but similar for ‘THC’ and ‘THC+CBD’ (t(47)=0.986, p=0.989, MD=0.188). The drug by time interaction was driven by ratings during ‘PLA’ falling from T1 to T2 (t(47)=4.768, p<0.001, MD=0.792), while ratings during ‘THC’ remained similar (t(47)=0.150, p=1, MD=0.042) and ratings during ‘THC+CBD’ increased from T1 to T2 (t(47)=3.244, p=0.022, MD=0.792). There was an interaction between age and time (F(4,184)=3.422, p=0.021, η^2^_p_=0.069), which was driven by no significant changes in ‘anxious’ ratings from T1 to T3 in adolescents (T1 to T2: t(47)=0.591, MD=0.097, p=1, T2 to T3: t(47)=1.046, MD=0.181, p=1), followed by significant reductions in ratings after T3 (T3 to T4: t(47)=3.352, MD=0.431, p=0.016., T3 to T5: t(47)=4.238, MD=0.875, p=0.001. While in adults, there was no significant difference from ratings at T1 and T2 (t(47)=0.422, MD=0.069, p=1), but ratings after T2 were significantly lower (T2 to T3: t(47)=4.001, MD=0.556, p=0.002, T2 to T4: t(47)=6.694, MD=1.167, p<0.001, T2 to T5: t(47)=7.735, MD=1.597, p<0.001).

**Secondary analysis**: The age-group by time interaction became non-significant when analysing non-imputed data (F(4,180)=2.554, p=0.056, η^2^_p_ =0.054).

**Paranoid (Figure S5d-f, Table S10e).** Greenhouse-Geisser correction was applied to time and drug by time. There was a main effect of drug (F(2,92)=7.302, p=0.001, η^2^_p_ =0.137) and time (F(4,184)=17.416, p<0.001, η^2^_p_ =0.275), and an interaction between the two (F(8, 368)=5.041, p=0.001, η^2^_p_ =0.099). Ratings of ‘paranoid’ were greater during ‘THC’ and THC+CBD’ relative to ‘PLA’ (t(92)=3.174, p=0.008, MD=0.471 and t(92)=3.648, p=0.002, MD=0.458, respectively), but similar during ‘THC’ and ‘THC+CBD’. The drug by time interaction was driven by ratings during ‘THC+CBD’ increasing from T1 to T2 (t(47)=4.029, p=0.002, MD=1) and decrease from T2 to T5 (t(47)=4.393, p<0.001, MD=1.104), while there was a non-significant increase in ratings during ‘THC’ from T1 to T2, followed by significant decrease from T2 to T5 (t(47)=4.089, p=0.002, MD=0.813). For ‘PLA’ ratings remained similar across all time points (T2 to T5: t(47)=2.024, p=0.488, MD=0.146).

**Secondary analysis:** A trend main effect of age-group emerged when using non-imputed data (F(2,92)=3.529, p=0.067, η^2^_p_ =0.073), this was driven by adolescents rating higher than adults (t(57)=1.88, p=0.067, MD=0.308).

**Happy (Figure S5g-i, Table S10f).** There was a significant interaction between age-group and drug for ‘happy’ (F(2,92)= 4.637, p=0.012, η^2^_p_=0.092), but no main effect of age-group or drug. This interaction appears to be driven by adults rating higher than adolescents for ‘happy’ during ‘PLA’ (t(47)=2.741, p=0.009, MD=1.042), but responded similarly to each other during ‘THC’ (t(47)=1.328, p=0.191, MD=0.533) and ‘THC+CBD’ (t(47)=0.659, p=0.5135, MD=0.258). There was no main effect of time, and all other interactions were non-significant, however there was a trend interaction between age and time (F(3,138)=2.564, p=0.07, η^2^_p_=0.053). Across time, adolescents’ ratings for ‘happy’ were higher during ‘THC’ (M=6.85 SE=0.284) and ‘THC+CBD’ (M=6.867, SE=0.277) and lower during ‘PLA’ (MD=6.433, SE=0.269). While adults showed the opposite pattern; across time their ratings were highest during ‘PLA’ (M=7.475, SE=0.269) and lower during ‘THC’ (M=7.383, SE=0.284) and ‘THC+CBD’ (M=7.125, SE=0.277)

**Secondary analysis:** This pattern of results was almost identical whether missing data were imputed or not.

**Want cannabis (Figure S5j-l, Table S10g).** Greenhouse-Geisser correction was applied to time and drug by time. For ‘want cannabis’ there was a main effect of age (F(1,46)=5.606, p=0.022, η^2^_p_=0.109) and time (F(4, 184)=7.796, p=0.001, η^2^_p_=0.145). Adolescents rated higher than adults (t(46)=2.370, p=0.022, MD=1.194) across drug. While ratings of ‘want cannabis’ decreased over time.

**Secondary analysis:** These results were almost identical whether missing data were imputed or not.


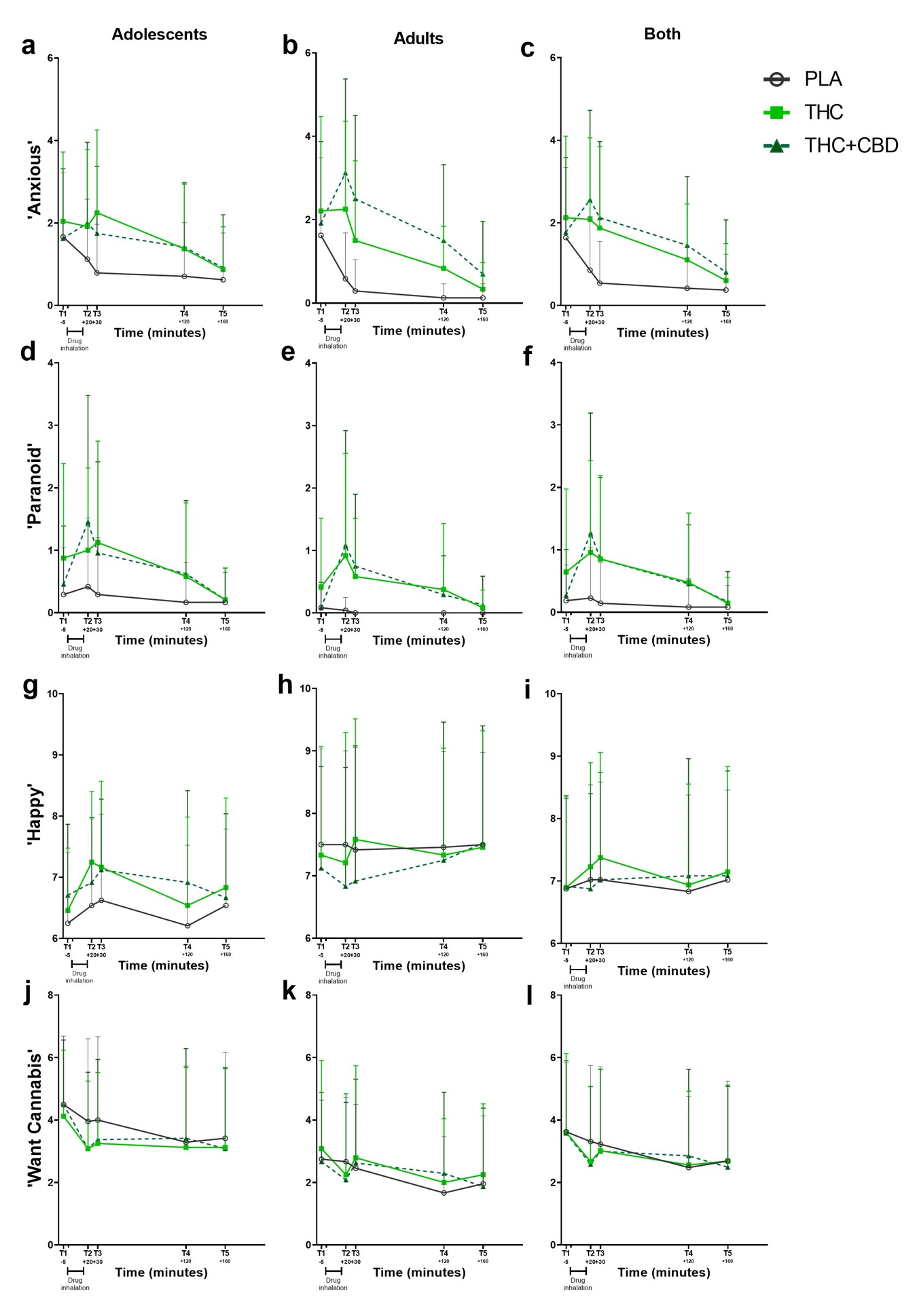


**Figure S5.** Mean values for subjective effect VASs (‘anxious’, ‘paranoid’, ‘want cannabis’, and ‘happy’) with 95% confidence intervals over time for adolescents and adults (n=24 per group for full datasets) during ‘PLA’, ‘THC’ and ‘THC+CBD’ conditions. VASs ranged from 0=‘not at all’ to 10=‘extremely’. Data from the two age groups has been combined to show overall drug effects in c, f, i, and l (n=48). Relative to the start of drug inhalation (time-inh) the time-points correspond as following: T1 = -5 min, T2 = +20 min, T3 = +30 min, T4 = +120 min, and T5 = +160 min. VAS=visual analogue scale. Data is missing for 1 adult during T5 of ‘THC+CBD’.

**Table S10a-g.** Results from 2x3x4 and 2x3x5 mixed ANOVA analyses of subjective effect VASs**,** showing main effects and interactions. Where each analysis had a within subject factors of drug (‘PLA’, ‘THC’, and ‘THC+CBD’) and time (T1-T5, or T2-T5*) and a between subject factor of age-group (adolescent, n=24; and adult n=24). ‘Feel drug effect’, ‘like drug effect’ and ‘dislike drug effect’ were completed T2-T5; ‘anxious’, ‘paranoid’, ‘want cannabis’ and ‘happy’ were completed T1-T5.

| 1. **Feel Drug Effect** | | | | |
| --- | --- | --- | --- | --- |
|  | **F** | **Df** | **P** | **η^2^_p_** |
| *Age* | 3.830 | 1,46 | 0.056 | 0.077 |
| ***Drug*** | **284.486** | **2,92** | **<0.001** | **0.861** |
| ***Time*** | **323.986** | **3, 138** | **<0.001** | **0.876** |
| ***Time*Drug*** | **61.846** | **6, 276** | **<0.001** | **0.573** |
| *Age*Drug* | 1.012 | 2,92 | 0.353 | 0.022 |
| *Age*Time* | 0.672 | 3, 138 | 0.527 | 0.014 |
| *Age*Drug*Time* | 0.701 | 6, 276 | 0.568 | 0.015 |

| 1. **Like Drug Effect** | | | | |
| --- | --- | --- | --- | --- |
|  | **F** | **Df** | **P** | **η^2^_p_** |
| *Age* | 0.638 | 1, 46 | 0.428 | 0.014 |
| ***Drug*** | **48.811** | **2, 92** | **<0.001** | **0.515** |
| ***Time*** | **34.495** | **3, 138** | **<0.001** | **0.429** |
| ***Time*Drug*** | **6.432** | **6, 276** | **<0.001** | **0.123** |
| *Age*Drug* | 0.357 | 2,92 | 0.609 | 0.008 |
| *Age*Time* | 2.250 | 3, 138 | 0.112 | 0.047 |
| *Age*Drug*Time* | 0.475 | 6, 276 | 0.778 | 0.010 |

| 1. **Dislike Drug Effect** | | | | |
| --- | --- | --- | --- | --- |
|  | **F** | **Df** | **P** | **η^2^_p_** |
| *Age* | 0.374 | 1,46 | 0.544 | 0.008 |
| *Drug* | 2.998 | 2,92 | 0.074 | 0.061 |
| ***Time*** | **11.416** | **3, 138** | **<0.001** | **0.199** |
| ***Time*Drug*** | **4.350** | **6, 276** | **0.001** | **0.086** |
| *Age*Drug* | 0.886 | 2,92 | 0.384 | 0.190 |
| ***Age*Time*** | **3.287** | **3, 138** | **0.023** | **0.067** |
| *Age*Drug*Time* | 0.625 | 6, 276 | 0.699 | 0.013 |

| 1. **Anxious** | | | | |
| --- | --- | --- | --- | --- |
|  | **F** | **Df** | **p** | **η^2^_p_** |
| *Age* | 1.102 | 1, 46 | 0.751 | 0.002 |
| ***Drug*** | **15.603** | **2, 92** | **<0.001** | **0.253** |
| ***Time*** | **42.463** | **4, 184** | **<0.001** | **0.480** |
| ***Drug*Time*** | **6.514** | **8, 368** | **<0.001** | **0.124** |
| *Drug*Age* | 2.868 | 2, 92 | 0.062 | 0.059 |
| ***Age*Time*** | **3.422** | **4, 184** | **0.021** | **0.069** |
| *Age*Drug*Time* | 1.652 | 8, 368 | 0.205 | 0.035 |

| 1. **Paranoid** | | | | |
| --- | --- | --- | --- | --- |
|  | **F** | **Df** | **P** | **η^2^_p_** |
| *Age* | 2.597 | 1, 46 | 0.114 | 0.053 |
| ***Drug*** | **7.302** | **2, 92** | **0.001** | **0.137** |
| ***Time*** | **17.416** | **4, 184** | **<0.001** | **0.275** |
| ***Time*Drug*** | **5.041** | **8, 368** | **0.001** | **0.099** |
| *Age*Drug* | 0.012 | 2, 92 | 0.988 | <0.001 |
| *Age*Time* | 0.522 | 4, 184 | 0.629 | 0.011 |
| *Age*Drug*Time* | 0.503 | 8, 368 | 0.734 | 0.011 |

| 1. **Happy** | | | | |
| --- | --- | --- | --- | --- |
|  | **F** | **Df** | **P** | **η^2^_p_** |
| *Age* | 2.862 | 1,46 | 0.097 | 0.059 |
| *Drug* | 0.837 | 2,92 | 0.436 | 0.018 |
| *Time* | 1.263 | 3, 138 | 0.286 | 0.027 |
| *Time*Drug* | 1.150 | 6, 276 | 0.334 | 0.024 |
| ***Age*Drug*** | **4.637** | **2,92** | **0.012** | **0.092** |
| *Age*Time* | 2.564 | 3, 138 | 0.070 | 0.053 |
| *Age*Drug*Time* | 1.120 | 6, 276 | 0.351 | 0.024 |

| 1. **Want Cannabis** | | | | |
| --- | --- | --- | --- | --- |
|  | **F** | **Df** | **P** | **η^2^_p_** |
| ***Age*** | **5.606** | **1, 46** | **0.022** | **0.109** |
| *Drug* | 0.234 | 2, 92 | 0.719 | 0.005 |
| ***Time*** | **7.796** | **4, 184** | **0.001** | **0.145** |
| *Time*Drug* | 1.547 | 8, 368 | 0.169 | 0.033 |
| *Age*Drug* | 0.738 | 2, 92 | 0.441 | 0.016 |
| *Age*Time* | 0.675 | 4, 184 | 0.512 | 0.014 |
| *Age*Drug*Time* | 0.356 | 8, 368 | 0.894 | 0.008 |

**Significant results in bold.** *Relative to the start of drug inhalation (time-inh) the time-points correspond as following: T1 = -5 min, T2 = +20 min, T3 = +30 min, T4 = +120 min, and T5 = +160 min.

### 3.5.2 Area under curve (AUC) and peak analysis for Subjective Effects-VAS over time

**Feel drug effect**

*AUC*

There was no significant interaction between drug and age-group (F(2, 92)=1.151, p=0.321). There was a main effect of drug (F(1.641,75.46)=251.8, p<0.001) and no main effect of age-group (F(1,46)=2.342, p=0.133). Across age-group, significant differences were: ‘THC’ > ‘PLA’, ‘THC+CBD’ > ‘PLA’, and ‘THC+CBD’ > ‘THC’.

*Peak effect*

There was no significant interaction between drug and age-group (F(2, 92)=0.398, p=0.673). There was a main effect of drug (F(1.563, 71.91)=284.4, p<0.001) and a main effect of age-group (F(1, 46)=8.738, p=0.0049). Across age-group, significant differences were: ‘THC’ > ‘PLA’, and ‘THC+CBD’ > ‘PLA’. Across drug, adolescents were significantly greater than adults.

**Like Drug effect**

*AUC*

there was no significant interaction between drug and age-group (F(2, 92)=0.343, p=0.710). There was a main effect of drug (F(1.325, 60.95)=46.05, p<0.001) and no main effect of age-group (F(1, 46)=0.202, p=0.656. Across age-group, significant differences were: ‘THC’ > ‘PLA’, and ‘THC+CBD’ > ‘PLA’.

*Peak effect*

There was no significant interaction between drug and age-group (F(2, 92)=0.424, p=0.656). There was a main effect of drug (F(1.274, 58.61)=71.620, p<0.001) and no main effect of age-group (F(1, 46)=0.261, p=0.612). Across age-group, significant differences were: ‘THC’ > ‘PLA’, and ‘THC+CBD’ > ‘PLA’.

**Dislike drug effect**

*AUC*

There was no significant interaction between drug and age-group (F(2, 92)=0.545, p=0.582). There was no main effect of drug (F(1.401, 64.43)=1.834, p=0.178) and no main effect of age-group (F(1, 46)=0.008, P=0.931).

*Peak effect*

There was no significant interaction between drug and age-group (F(2, 92)=2.729, p=0.071). There was a main effect of drug (F(1.673, 76.96)=8.671, p=0.001) and no main effect of age-group (F(1, 46) = 1.565, p=0.217). Across age-group, significant differences were: ‘THC+CBD’ > ‘PLA’.

**Anxious**

*AUC*

There was no significant interaction between drug and age-group (F(2, 92)=3.053, p=0.052). There was a main effect of drug (F(1.992, 91.63)=15.97, p<0.001) and no main effect of age-group (F(1, 46)=0.496, p=0.485). Across age-group, significant differences were: ‘THC’ > ‘PLA’, and ‘THC+CBD’ > ‘PLA’.

*Peak effect*

There was no significant interaction between drug and age-group (F(2, 92)=1.540, p=0.220). There was a main effect of drug (F(1.970, 90.63)=10.04, p<0.001) and no main effect of age-group (F(1, 46)=0.569, p=0.455). Across age-group, significant differences were: ‘THC’ > ‘PLA’, and ‘THC+CBD’ > ‘PLA’.

**Paranoid**

*AUC*

There was no significant interaction between drug and age-group (F(2, 92)=0.118, p=0.889). There was a main effect of drug (F(1.812, 83.36)=6.474, p=0.003) and no main effect of age-group (F(1, 46)=2.408, p=0.128). Across age-group, significant differences were: ‘THC’ > ‘PLA’ and ‘THC+CBD’ > ‘PLA’.

*Peak effect*

There was no significant interaction between drug and age-group (F(2, 92)=0.056, p=0.946). There was a main effect of drug (F(1.884, 86.65)=9.761, p<0.001) and no main effect of age-group (F(1, 46)=1.070, p=0.306). Across age-group, significant differences were: ‘THC’ > ‘PLA’, and ‘THC+CBD’ > ‘PLA’.

**Happy**

*AUC*

There was a significant interaction between drug and age-group (F(2, 92)=4.126, p=0.019). There was no main effect of drug (F(1.829, 84.13)=0.731, p=0.473) and no main effect of age-group (F(1, 46)=2.318, p=0.135). This interaction was driven by adults rating higher than adolescents on ‘PLA’ (t(47)=2.451, p=0.016), but the two groups rating similarly on ‘THC’ (t(47)=1.365, p=0.175) and ‘THC+CBD’ (t(47)=0.306, p=0.760).

*Peak effect*

There was no significant interaction between drug and age-group (F(2, 92)=2.701, p=0.073). There was a main effect of drug (F(1.894, 87.13)=4.999, p=0.010) and a main effect of age-group (F(1, 46)=4.458, P=0.040). Across age-group, significant differences were: ‘THC’ > ‘PLA’. Across drug, adolescents were significantly greater than adults.

**Want cannabis**

*AUC*

There was no significant interaction between drug and age-group (F(2, 92)=0.615, p=0.543). There was no main effect of drug (F(1.344, 61.81)=0.235, p=0.701) and a main effect of age-group (F(1, 46)=4.139, p=0.048).

*Peak effect*

There was no significant interaction between drug and age-group (F(2, 92)=0.794, p=0.455). There was no main effect of drug (F(1.735, 79.79)=0.598, p=0.530) and a main effect of age-group (F(1, 46)=5.222, p=0.027). Across drug, adolescents were significantly greater than adults.

### 3.5.3 Prose Recall

**Prose Recall immediate**

For immediate prose recall score, there was no interaction between drug and age-group (F(2,92)=0.236, p=0.790, η^2^_p_=0.005). There was a main effect of drug (F(2,92)=28.535, p<0.001, η^2^_p_= 0.383), but no main effect of age-group (F(1,46)=0.079, p=0.779, η^2^_p_=0.002). Across age-groups, scores during ‘THC’ and ‘THC+CBD’ were lower than ‘PLA’ (t(47)=5.932, p<0.001, MD=3.229 and t(47)=6.925, p<0.001, MD=3.573 respectively). However, there was no significant difference between ‘THC’ and ‘THC+CBD’ (t(47)=0.681, p=1, MD=-0.344).

| **Table S11.** Prose Recall Immediate and Delayed 2x2x3 mixed ANOVA, with within subject factors of time (immediate and delayed*) and drug (‘PLA’, ‘THC’, and ‘THC+CBD’), and a between subject factor of age-group (adolescent, n=24; and adult n=24). | | | | |
| --- | --- | --- | --- | --- |
|  | **F** | **df** | **p** | **η^2^_p_** |
| *Age* | 0.078 | 1,46 | 0.781 | 0.002 |
| ***Drug*** | **26.203** | **2,92** | **<0.001** | **0.363** |
| ***Time*** | **63.386** | **1, 46** | **<0.001** | **0.579** |
| ***Time*Drug*** | **3.462** | **2, 92** | **0.036** | **0.070** |
| *Age*Drug* | 0.432 | 2,92 | 0.711 | 0.007 |
| *Age*Time* | <0.001 | 1,46 | 1.000 | <0.001 |
| *Age*Drug*Time* | 2.327 | 2,92 | 0.103 | 0.048 |

**Significant results in bold. ***Immediate was completed straight away after listening to the short story (20 minutes after the start of drug inhalation) and delayed was completed after a 100 minute delay (120 minutes after the start of drug inhalation) filled with unrelated tasks.

**Prose Recall immediate and delayed 2x2x3 ANOVA.**

Immediate and delayed prose recall scores were analysed in a 2x2x3 mixed ANOVA. This analysis revealed there was no age-group by drug interaction, nor was there an age-drug-time interaction (Table S11). There was a main effect of drug (F(2,92)=26.203, p<0.001, η^2^_p_ =0.363) and time (F(1,46)=63.386, p<0.001, η^2^_p_ =0.579). The same pattern of drug effect was found i.e. scores were lower during ‘THC’ and ‘THC+CBD’ relative to ‘PLA’, but there was no significant difference between ‘THC’ and ‘THC+CBD’ conditions. Scores were lower during delayed prose recall relative to immediate recall (t(47)=7.969, p<0.001, MD=1.028). There was a significant interaction between the drug and time (F(2,92)=3.462, p=0.036, η^2^_p_ =0.070). This interaction was driven by a greater reduction in score from immediate to delayed during ‘PLA’ (t(47)=6.560, p<0.001, MD=1.417) compared with smaller reductions during ‘THC’ (t(47)=5.458, p<0.001, MD=0.927) and ’THC+CBD’ (t(47)=3.486, p=0.001, MD=0.740). There was no main effect of age, and age interactions with drug and time were non-significant.

### 3.5.4 PSI subscales

PSI subscales scores (Table S12) were analysed individually, assumptions were first checked. Greenhouse-Geisser correction was applied to the Cognitive Disorganisation subscale. There were no interactions between drug and age-group across subscales (Table S13). There was a main effect of drug for the subscales Perceptual Distortion (F(2,92)=13.630, p<0.001, η^2^_p_=0.229); Cognitive Disorganisation (F(2,92)=34.677, p<0.001, 0.002, η^2^_p_=0.430); Mania (F(2,92)=6.475, p=0.002, η^2^_p_ =0.123); and Paranoia (F(2,92)=3.151 , p=0.047, η^2^_p_ =0.064). There was no main drug effect for Anhedonia (F(2,92)=2.441, p=0.093, η^2^_p_ =0.050) or Delusory Thinking (F(2,92)=1.435, p=0.243, η^2^_p_ =0.030). Across subscales there was no main effect of age.

Pairwise comparisons for subscales with a main effect of drug are displayed in Table S14. Subscale scores were greater in ‘THC’ and ‘THC+CBD’ conditions relative to ‘PLA’ for Cognitive Disorganisation (t(47)=4.841, p<0.001, MD=3.938 and t(47)=7.721, p<0.001, MD=5.833, respectively) and Perceptual Distortion (t(47)=3.614, p=0.002, MD=1.667 and t(47)=4.689, p<0.001, MD=2.146, respectively). Additionally, scores for Cognitive Disorganisation were greater during ‘THC+CBD’ relative to ‘THC’ (t(47)=3.464, p=0.003, MD=1.896). For the Mania subscale, scores were greater during ‘THC+CBD’ relative to ‘PLA’ (t(47)=3.679, p=0.002, MD=1.250). All other between drug comparisons were non-significant (Table S14).

**Table S12.** PSI subscale scores for adolescents (n=24), adults (n=24), and both (n=48) across each drug session. Mean (SD), [median, min-max].

|  | **Adolescent (n=24)** | **Adult (n=24)** | **Both (n=48)** |
| --- | --- | --- | --- |
| **Cognitive Disorganisation** | |  |  |
| 'PLA' | 4.71 (5.58), [3, 0-20] | 1.96 (2.71), [1, 0-12] | 3.33 (4.55), [2, 0-20] |
| 'THC' | 8.04 (5.97), [7, 0-20] | 6.50 (4.31), [7, 0-14] | 7.27 (5.21), [7, 0-20] |
| 'THC+CBD' | 9.42 (4.37), [9, 0-18] | 8.92 (4.90), [9.5, 0-17] | 9.17 (4.60), [9, 0-18] |
| **Perceptual Distortion** | |  |  |
| 'PLA' | 1.67 (2.43), [0.5, 0-8] | 1.08 (2.04), [0, 0-7] | 1.38 (2.24), [0, 0-8] |
| 'THC' | 3.58 (3.40), [3, 0-11] | 2.50 (2.02), [2.5, 0-7] | 3.04 (2.82), [2.5, 0-11] |
| 'THC+CBD' | 3.54 (3.22), [3, 0-11] | 3.50 (2.15), [3.5, 0-8] | 3.52 (2.71), [3, 0-11] |
| **Delusory Thinking** | |  |  |
| 'PLA' | 1.88 (2.89), [0, 0-9] | 1.17 (2.50), [0.5, 0-12] | 1.52 (2.70), [0, 0-12] |
| 'THC' | 2.29 (2.60), [1.5, 0-9] | 1.54 (2.02), [1, 0-7] | 1.92 (2.33), [1, 0-9] |
| 'THC+CBD' | 2.42 (2.32), [2, 0-7] | 1.96 (2.63), [1, 0-10] | 2.19 (2.46), [1, 0-10] |
| **Anhedonia** |  |  |  |
| 'PLA' | 4.29 (2.73), [4, 0-12] | 3.71 (2.65), [3, 0-10] | 4.00 (2.67), [4, 0-12] |
| 'THC' | 4.50 (2.30), [4.5, 0-9] | 4.88 (2.89), [4.5, 0-10] | 4.69 (2.59), [4.5, 0-10] |
| 'THC+CBD' | 4.29 (2.35), [4, 1-8] | 4.25 (2.67), [5, 0-10] | 4.27 (2.49), [5, 0-10] |
| **Paranoia** |  |  |  |
| 'PLA' | 0.67 (1.24), [0, 0-5] | 0.29 (0.81), [0, 0-3] | 0.48 (1.05), [0, 0-5] |
| 'THC' | 1.25 (2.05), [0, 0-7] | 0.38 (0.65), [0, 0-2] | 0.81 (1.57), [0, 0-7] |
| 'THC+CBD' | 1.21 (1.32), [1, 0-4] | 1.00 (2.02), [0, 0-8] | 1.10 (1.69), [0, 0-8] |
| **Mania** |  |  |  |
| 'PLA' | 3.21 (2.59), [3, 0-9] | 2.67 (1.43), [3, 0-6] | 2.94 (2.09), [3, 0-9] |
| 'THC' | 3.79 (2.81), [3, 0-9] | 3.58 (2.04), [4, 0-7] | 3.69 (2.43), [3, 0-9] |
| 'THC+CBD' | 3.92 (2.72), [3, 0-12] | 4.46 (2.08), [5, 0-8] | 4.19 (2.41), [4.5, 0-12] |

PSI= psychotomimetic states inventory.

**Table S13**. Two-way mixed ANOVA results for PSI subscale scores with a within subjects factor of drug (‘PLA’, ‘THC’, and ‘THC+CBD’) and a between subjects factor of age-group (adolescent, n=24; and adult n=24).

| **PSI Subscale** |  | **F** | **df** | **p** | **η_p_^2^** |
| --- | --- | --- | --- | --- | --- |
| **Delusory Thinking** | Drug | 1.435 | 2,92 | 0.243 | 0.030 |
|  | Age-group | 1.296 | 1,46 | 0.261 | 0.027 |
|  | Drug*Age | 0.079 | 2,92 | 0.924 | 0.002 |
| **Perceptual Distortion** | **Drug** | **13.630** | **2,92** | **<0.001** | **0.229** |
|  | Age-group | 1.025 | 1,46 | 0.317 | 0.022 |
|  | Drug*Age | 0.729 | 2,92 | 0.485 | 0.016 |
| **Cognitive disorganisation** | **Drug** | **34.677** | **2,92** | **<0.001** | **0.430** |
|  | Age-group | 2.118 | 1,46 | 0.152 | 0.044 |
|  | Drug*Age | 1.241 | 2,92 | 0.294 | 0.026 |
| **Anhedonia** | Drug | 2.441 | 2,92 | 0.093 | 0.050 |
|  | Age-group | 0.016 | 1,46 | 0.900 | <0.001 |
|  | Drug*Age | 1.175 | 2,92 | 0.313 | 0.025 |
| **Mania** | **Drug** | **6.475** | **2,92** | **0.002** | **0.123** |
|  | Age-group | 0.017 | 1,46 | 0.898 | <0.001 |
|  | Drug*Age | 1.259 | 2,92 | 0.289 | 0.027 |
| **Paranoia** | **Drug** | **3.151** | **2,92** | **0.047** | **0.064** |
|  | Age-group | 2.836 | 1,46 | 0.117 | 0.053 |
|  | Drug*Age | 0.970 | 2,92 | 0.383 | 0.021 |

**Significant results in bold.** PSI = psychotomimetic states inventory scale; df=degrees of freedom. This scale was completed approximately 130 minutes after the start of drug inhalation.

#### Table S14. Pairwise comparisons (following mixed-ANOVA) of each PSI subscale scores between drug conditions following a main effect of drug.

| **PSI Subscale** | **'THC' > 'PLA'** | **'THC+CBD' > 'PLA'** | **'THC+CBD' > 'THC'** |
| --- | --- | --- | --- |
| Cognitive disorganisation | **t(47)=4.841, p<0.001, MD=3.938** | **t(47)=7.722, p<0.001, MD=5.833** | **t(47)=3.464, p=0.003, MD=1.896** |
| Perceptual distortion | **t(47)=3.614, p=0.002, MD=1.667** | **t(47)=4.689, p<0.001, MD=2.146** | t(47)=-1.298, p=0.602, MD=0.479 |
| Mania | t(47)=2.064, p=0.134, MD=0.750 | **t(47)=3.679, p=0.002, MD=1.250** | t(47)=1.448, p=0.463, MD=0.500 |
| Paranoia | t(47)=1.553, p=0.382, MD=0.333 | t(47)=2.273, p=0.083, MD=0.625 | t(47)=1.15, p=0.771, MD=0.292 |

**Significant results in bold.** PSI=psychotomimetic states inventory; MD=mean difference

### 3.5.5 PANSS

**PANSS Positive Subscale**

There was no significant interaction between age-group and drug for the Positive-PANSS score (F(2,92)=0.043, p=0.958, η^2^_p_=0.001). There was a main effect of drug (F(2,92)=33.846 p<0.001, η^2^_p_=0.424), but not of age-group (F(2,92)=0.340, p=0.854, η^2^_p_=0.001). Positive psychotic symptoms were greater on ‘THC’ (MD=2.667, 95% CI 1.503–3.831, t(47)=5.694, p<0.001) and ‘THC+CBD’ (MD=3.417, 95% CI 2.493–4.341, t(47)=9.189, p<0.001) compared with ‘PLA’. However, there was no significant difference between ‘THC’ and ‘THC+CBD’ (MD=-0.750, 95% CI -1.899–0.399, t(47)=1.622, p=0.335). This pattern of results was further supported by an analysis of the number of participants who had clinically significant positive psychotic reactions（score >3 greater than minimum score of 7, i.e. >10) (see supplementary materials).

The pattern of results found in mixed-ANOVA (see main text) was further supported via binary logistic generalised estimating equation models (GEE). There was no age-group by drug interaction (Wald χ^2^ (2)=1.032, p=0.597); there was a main effect of drug (Wald χ^2^ (2)=25.733, p<0.001), but no main effect of age-group (Wald χ^2^ (1)=0.001, p=0.891). Across age-group, participants were more likely to have a psychotic response (i.e. PANSS Positive subscale score >10) during both ‘THC’ (OR=19.489, p<0.001) and ‘THC+CBD’ (OR=23.164, p<0.001) than during ‘PLA’.

**PANSS Negative Subscale**

There was no significant interaction between age-group and drug for the Negative-PANSS score (F(2,92)=1.132, p=0.327, η^2^_p_=0.024). There was a main effect of drug (F(2,92)=9.392, p<0.001, η^2^_p_=0.170), but not of age-group (F(2,92)=1.615, p=0.210, η^2^_p_=0.034). Negative psychotic symptoms were greater on ‘THC’ (MD=0.646, 95% CI 0.138–1.154, t(47)=3.160, p=0.008) and ‘THC+CBD’ (MD=0.958, 95% CI 0.384–1.532, t(47)=4.147, p<0.001) than ‘PLA’. However, there was no significant difference between ‘THC’ and ‘THC+CBD’ (MD=-0.312, 95% CI -0.908–0.283, t(47)=1.304, p=0.596). This pattern of results was further supported by an analysis of the number of participants who had clinically significant increases in negative psychotic symptoms (see supplementary materials).

The pattern of results found in mixed-ANOVA (see main text) was further supported via chi-square and McNemar’s tests. We could not conduct GEEs, because there were zero cases of clinically significant negative psychotic reactions during ‘PLA’. For both ‘THC’ and ‘THC+CBD’, there were no significant differences in the number of adolescents and adults having clinically significant reactions (both tests: χ_1_^2^=0.223, p=0.637). Across age-group, the same number of participants in ‘THC’ and ‘THC+CBD’ had clinically significant reactions, so there was no significant effect of drug (p=1).

#### **Table S15**. Descriptive Statistics for PANSS Positive and Negative subscale scores across each drug condition for adolescents (n=24) and adults (n=24), mean (SD) [median, min-max].

| **Subscale** | **Condition** | **Adolescents (n=24)** | **Adults (n=24)** | **Both (n=48)** |
| --- | --- | --- | --- | --- |
| **PANSS Positive** | 'PLA' | 7.46 (1.18) [7, 7-12] | 7.33 (0.82) [7, 7-10] | 7.40 (1.01) [7, 7-12] |
|  | 'THC' | 10.17 (3.58) [10, 7-23] | 9.96 (3.03) [10, 7-19] | 10.06 (3.28) [10, 7-23] |
|  | 'THC+CBD' | 10.79 (3.12) [10, 7-18] | 10.83 (2.10) [10, 8-15] | 10.81 (2.63) [10, 7-18] |
| **PANSS Negative** | 'PLA' | 7.17 (0.38) [7, 7-8] | 7.08 (0.41) [7, 7-9] | 7.13 (0.39) [7, 7-9] |
|  | 'THC' | 8.13 (1.87) [7, 7-15] | 7.42 (0.97) [7, 7-11] | 7.77 (1.54) [7, 7-15] |
|  | 'THC+CBD' | 8.17 (1.63) [7.5, 7-13] | 8.00 (1.41) [7.5, 7-13] | 8.08 (1.51) [7.5, 7-13] |

PANSS= positive and negative syndrome scale.

**Table S16**. Clinically significant psychotic reaction on PANSS Positive and Negative (i.e. score >3 greater than minimum) for adolescents (n=24) and adults (n=24). n (%) is shown.

| **Subscale** | **Condition** | **Adolescents (n=24)** | | **Adults (n=24)** | | **Both (n=24)** | | |
| --- | --- | --- | --- | --- | --- | --- | --- | --- |
|  |  | **Yes** | **No** | **Yes** | **No** | **Yes** | **No** |  |
| PANSS Positive | 'PLA' | 2 (8.3%) | 22 (91.7%) | 1 (4.2%) | 23 (95.8%) | 3 (6.25%) | 45 (93.75%) |  |
|  | 'THC' | 13 (54.2%) | 11 (45.8%) | 14 (58.3%) | 10 (41.7%) | 27 (56.25%) | 21 (43.75%) |  |
|  | 'THC+CBD' | 13 (54.2%) | 11 (45.8%) | 16 (66.7%) | 8 (36.4%) | 29 (60.42%) | 19 (39.58%) |  |
| PANSS Negative | 'PLA' | 0 (0%) | 24 (100%) | 0 (0%) | 24 (100%) | 0 (0%) | 48 (100%) |  |
|  | 'THC' | 4 (16.7%) | 20 (83.3%) | 1 (4.2%) | 23 (95.8%) | 5 (10.42%) | 43 (89.58%) |  |
|  | 'THC+CBD' | 4 (16.7%) | 20 (83.3%) | 2 (8.3%) | 22 (91.7%) | 6 (12.5%) | 42 (87.5%) |  |

PANSS= positive and negative syndrome scale.

**
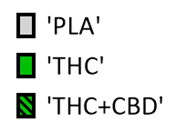
**

**Figure S6**. PANSS Positive and Negative subscale score, presented as mean values with 95% confidence intervals for adolescents (n=24) and adults (n=24) during ‘THC’, ‘THC+CBD’ and ‘PLA’ conditions. Main effect of drug across age-groups displayed in ‘Both’ (b and d) graphs where participant data is combined (n=48). **p<0.01, ***p<0.001. PANSS= positive and negative syndrome scale.

### 3.5.6 Plasma THC and CBD levels

#### 3.5.6.1 Plasma levels at T1 (pre-drug administration)

**Table S17a.** Plasma THC and CBD concentrations (ng.ml^-1^) in adolescents and adults at T1 (5 minutes before start of cannabis inhalation). Adolescent (n=24), adult (n=24) and both (n=48) for complete datasets. Mean (SD) [median, range].

|  | **Adolescent (n=24)** | | | **Adult (n=24)** | | | **Both (n=48)** | | |
| --- | --- | --- | --- | --- | --- | --- | --- | --- | --- |
|  | **THC (ng.ml^-1^)** | **n missing** | **n** <**LLQ** | **THC (ng.ml^-1^)** | **n missing** | **n <LLQ** | **THC (ng.ml^-1^)** | **n missing** | **n <LLQ** |
| 'PLA' | *0.05 (0.25) [0, 0-1.18] | 1 | 0 | *0.05 (0.25) [0, 0-1.23] | 0 | 2 | ⱡ 0.05 (0.25) [0, 0-1.23] | 2 | 2 |
| 'THC' | 0 (0) [0, 0-0] | 0 | 1 | *0.02 (0.12) [0, 0-0.58] | 0 | 1 | *0.01 (0.08) [0, 0-0.58] | 2 | 2 |
| 'THC+CBD' | *0.03 (0.15) [0, 0-0.71] | 1 | 3 | 0 (0) [0, 0-0] | 0 | 1 | *0.02 (0.1) [0, 0-0.71] | 4 | 4 |
|  |  |  |  |  |  |  |  |  |  |
|  | **CBD (ng.ml^-1^)** |  |  | **CBD (ng.ml^-1^)** |  |  | **CBD (ng.ml^-1^)** |  |  |
| 'PLA' | 0 (0) [0, 0-0] | 1 | 0 | 0 (0) [0, 0-0] | 0 | 3 | 0 (0) [0, 0-0] | 1 | 3 |
| 'THC' | 0 (0) [0, 0-0] | 0 | 3 | 0 (0) [0, 0-0] | 0 | 3 | 0 (0) [0, 0-0] | 0 | 6 |
| 'THC+CBD' | 0 (0) [0, 0-0] | 1 | 2 | 0 (0) [0, 0-0] | 0 | 4 | 0 (0) [0, 0-0] | 1 | 6 |

LLQ= lower limit of quantification (i.e. <0.5 ng.ml^-1^), these were replaced with 0s for descriptive statistics. *n=1 THC plasma concentration >0 ng.ml^-1^; ⱡ n=2 THC plasma concentration >0 ng.ml^-1^.

#### 3.5.6.2 Plasma levels at T2 (20 minutes after the start of drug inhalation)

For plasma THC level, there was no significant age-group by drug interaction (F(2,90.311)=1.606, p=0.206). There was a main effect of drug (F(2,94.374)=136.279, p<0.001), but no effect of age-group (F(1,47.045=2.455 p=0.124). Pairwise comparisons revealed that THC levels were greater during ‘THC’ and ‘THC+CBD’ relative to ‘PLA’ (t(90.059)=8.964, p<0.001, MD=14.813 and t(90.367)=16.490, p<0.001, MD=27.717, respectively). Moreover, there were higher levels of THC during ‘THC+CBD’ relative to ‘THC’ (t(90.514)=7.760, p<0.001, MD=12.903).

For plasma CBD levels there was an age-group by drug interaction (F(2,91.252)=7.006, p=0.001), and main effects of both drug (F(2,91.252)=191.898, p<0.001) and age-group (F(1,49.006)=7.262, p=0.010). The age-group by drug interaction was driven by greater CBD concentration during ‘THC+CBD’ in adults compared to adolescents (t(133)=4.569, p<0.001, MD=21.018), but no significant differences between adults and adolescents during ‘PLA’ or ‘THC’, when values were at or near zero. The main drug effect was driven by greater plasma CBD concentration during ‘THC+CBD’ relative to ‘THC’ and ‘PLA’ (t(91.434)=17.106, p<0.001, MD=54.753 and t(91.508)=16.945, p<0.001, MD=54.831, respectively). CBD concentrations during ‘THC’ and ‘PLA’ were similar (t(90.828)=0.024, p=1, MD=0.078).

These results did not significantly change when excluding the participant with unexpected plasma levels of THC.

**Table S17b**. Plasma THC and CBD concentrations (ng.ml^-1^) in adolescents and adults at T2 (20 minutes after start of cannabis inhalation). Adolescent (n=24), adult (n=24) and both (n=48) for complete datasets. Mean (SD) [range].

|  | **Adolescent (n=24)** | | | **Adult (n=24)** | | | **Both (n=48)** | | |
| --- | --- | --- | --- | --- | --- | --- | --- | --- | --- |
|  | **THC (ng.ml^-1^)** | **n missing** | **n <LLQ** | **THC (ng.ml^-1^)** | **n missing** | **n <LLQ** | **THC (ng.ml^-1^)** | **n missing** | **n <LLQ** |
| 'PLA' | 0.02 (0.12) [0.00-0.54] | 2 | 1 | 0.10 (0.47) [0.00-2.30] | - | 2 | 0.06 (0.35) [0.00-2.30] | 2 | 3 |
| 'THC' | 13.67 (6.74) [0.00-28.74] | - | - | 16.10 (7.48) [4.04-34.65] | - | - | 14.88 (7.15) [0.00-34.65] | 0 | 0 |
| 'THC+CBD' | 24.74 (9.00) [10.30-45.00] | 2 | - | 30.84 (17.51) [7.21-70.46] | 1 | - | 27.86 (14.20) [7.21-70.46] | 3 | 0 |
|  |  |  |  |  |  |  |  |  |  |
|  | **CBD (ng.ml^-1^)** |  |  | **CBD (ng.ml^-1^)** |  |  | **CBD (ng.ml^-1^)** |  |  |
| 'PLA' | 0 (0) [0-0] | 2 | 1 | 0 (0) [0-0] | - | 4 | 0 (0) [0-0] | 2 | 5 |
| 'THC' | 0 (0) [0-0] | - | 2 | 0.16 (0.54) [0.00-2.28] | - | 2 | 0.08 (0.39) [0-2.28] | 0 | 4 |
| 'THC+CBD' | 44.32 (15.33) [21.39-69.46] | 2 | - | 65.34 (34.84) [16.13-144.14] | 1 | - | 55.06 (28.84) [16.13-144.14] | 3 | 0 |

LLQ= lower limit of quantification (i.e. <0.5 ng.ml^-1^), these were replaced with 0s for descriptive statistics.


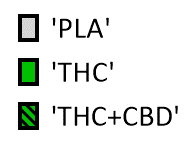


**Figure S7.** Mean values with 95% confidence intervals displayed with all data points for a) THC plasma levels at T2, c) CBD plasma levels at T2 and e) heart rate at T2 for adolescents and adults during ‘THC’, ‘THC+CBD’ and ‘PLA’ conditions. Main effect of drug across age-groups displayed in b, d, and f where their data is combined. **p<0.01, ***p<0.001. Relative to the start of drug administration T2= +20 minutes. Bpm = beats per minute.

### 3.5.7 Heart Rate (T2)

Greenhouse-Geisser correction was applied. There was no interaction between drug and age-group for heart rate at T2 (F(2,92)=0.034, p=0.953, η^2^_p_ =0.001). There was a large main effect of drug (F(2,92)=53.625), p<0.001, η^2^_p_ =0.538), but no main effect of age-group (F(1,46)=0.773, p=0.384, η^2^_p_ =0.017). Heart rate was higher during ‘THC’ and ‘THC+CBD’ than ‘PLA’ (t(47)=8.448, p<0.001, MD=18.604, and t(47)=8.746, p<0.001, MD=26.771, respectively). Additionally, heart rate during ‘THC+CBD’ was higher than ‘THC’ (t(47)=3.122, p=0.009, MD=8.167). This pattern of results was almost identical when using either imputed or non-imputed data.

**Table S18.** Heart rate at T2 (20 minutes after beginning cannabis inhalation). Adolescent (n=24), adult (n=24) for complete datasets. Mean (SD) [min-max].

| **Drug** | **Adolescents (n=24)** | **Adults (n=24)** | **Both (n=48)** |
| --- | --- | --- | --- |
| 'PLA' | 73.63 (10.47), [52.00-95.00] | 69.04 (15.57), [43.00-104.00] | 71.33 (13.33), [43.00-104.00] |
| 'THC' | *91.96 (15.93), [63.00-127.00] | 87.92 (22.04), [54.00-152.00] | *89.89 (19.19), [54.00-152.00] |
| 'THC+CBD' | 99.71 (24.00), [54.00-159.00] | 96.50 (21.60), [59.00-132.00] | 98.10 (22.65), [54.00-159.00] |

*n=1 missing.

### 3.5.8 Participant Drug Guess

Participants guessed which drug they had on each occasion (Table S19). Results from a GEE model with only main effects of drug and age-group, revealed a significant difference between drugs (Wald χ^2^(2)=25.678, p<0.001), but no difference between age-groups (Wald χ^2^(1)=0.178, p<0.673). Participants were worse at guessing correctly on ‘THC’ than ‘PLA’ (OR=0.049, p<0.001) and worse on ‘THC+CBD’ than ‘PLA’ (OR=0.132, p<0.001). The GEE model with main effects of age-group and drug, and an interaction effect of age-group by drug, revealed no significant interaction between drug and age-group (Wald χ^2^(2)=4.212, p=0.122).

On ‘PLA’, 43 (89.6%) participants (n=48) correctly guessed placebo, while 1 (2.1%) guessed ‘THC without CBD’ and 4 (8.3%) guessed ‘THC with CBD’. On ‘THC’, 14 (29.2%) of participants (n=47) guessed correctly, while 28 (58.3%) guessed ‘THC with CBD’, and 5 (10.4%) guessed placebo. On ‘THC+CBD’, 25 (52.1%) of participants (n=47) guessed correctly, while 22 (45.8%) guessed ‘THC without CBD’ and 0 (0%) participants guessed placebo.

**Table S19**. Participants’ guess regarding which drug they received at the end of each drug administration session. Adolescent (n=24), adult (n=24) for complete datasets. n(%).

|  | **Adolescents' Guess (n=24)** | | | |  | **Adults' Guess (n=24)** | | | | |  |
| --- | --- | --- | --- | --- | --- | --- | --- | --- | --- | --- | --- |
| **Actual Drug** | **'PLA'** | **'THC'** | **'THC+CBD’** | **n**  **missing** | | **'PLA'** | **'THC'** | **'THC+CBD’** | **n**  **missing** |  |  |
| **'PLA'** | 22 (91.7%) | 0 (0%) | 2 (8.3%) | - | | 21 (87.5%) | 1 (4.2%) | 2 (8.3%) | - |  |  |
| **'THC'** | 2 (8.3%) | 9 (37.5%) | 12 (50.0%) | 1 | | 3 (12.5%) | 5 (20.8%) | 16 (66.7%) | - |  |  |
| **'THC+CBD'** | 0 (0%) | 13 (54.2%) | 10 (41.7%) | 1 | | 0 (0%) | 9 (37.5%) | 15 (62.5%) | - |  |  |

##

## 3.6 Drug Administration

### 3.6.1 Deviations from inhalation procedure

**Time taken**: During ‘THC+CBD’, n=4 participants took >18 minutes to complete inhalation (n=3 adults and n=1 adolescent). These participants took an average of 19.40 minutes (min=18.33, max=20.80). During the ‘PLA’ and ‘THC’, all participants completed inhalation within the allocated 18 minutes (9 minutes per balloon).

**Incomplete inhalation**: During ‘THC+CBD’, n=1 adolescent did not inhale the entire contents of the balloon the second time it was filled (estimated 1/10^th^ of contents remained).

**Inhaled while sitting**: During ‘THC+CBD’ n=5 participants sat down for a portion of the inhalation procedure due to light-headedness (n=3 adolescents, n=2 adults). During ‘PLA’ and ‘THC’, all participants stood for the entire duration of cannabis inhalation.

### 3.6.2 Time taken to inhale vapourised cannabis.

The cumulative time taken for participants to inhale the contents of both cannabis vapour filled balloons is presented in Table S20.

Greenhouse-Geisser correction was applied. For time taken to inhale the contents of the two balloons filled with cannabis vapour, there was a main effect of drug (F(2,86)=28.434, p<0.001, η^2^_p_=0.398), but no main effect of age-group (F(2,43)=0.212, p=0.648, η^2^_p_=0.005), or drug by age-group interaction (F(2,86)=0.154, p=0.802, η^2^_p_=0.004). Participants took longer to inhale the contents of the balloons during ‘THC’ and ‘THC+CBD’ compared to ‘PLA’ (t(42)=2.864, p=0.019, MD=0.998 and t(42)=5.990, p<0.001, MD=3.426, respectively). Additionally, participants took longer to complete inhalation during ‘THC+CBD’ compared to ‘THC’ (t(42)=5.340, p<0.001, MD=2.429).

**Table S20.** Cumulative time taken (minutes) to inhale both balloons filled with cannabis vapour during each session. Adolescents (n=24) and adults (n=24) unless otherwise stated. Mean (SD), [median, min-max].

| **Condition** | **Adolescents (n=24)** | **Adults (n=24)** | **Both (n=24)** |
| --- | --- | --- | --- |
| 'PLA' | 8.26 (2.73), [7.84, 4.63-16.25] | 8.65 (2.49), [8.50, 4.22-12.87] | 8.45 (2.59) [8.47, 4.22-16.25] |
| 'THC' | 9.17 (3.07), [9.06, 5.08-18.00] | 9.92 (3.07), [9.63, 6.13-17.30] | 9.53 (3.06) [9.24, 5.08-18.00] |
| 'THC+CBD' | *11.85 (3.43), [12.22, 5.27-20.80] | *11.76 (4.37), [11.87, 5.57-19.30] | **ⱡ**11.81 (3.87) [12.15, 5.27-20.80] |

*n=1 missing; **ⱡ** n=2 missing.

### 3.6.3 Acute Coughing Rating

Greenhouse-Geisser correction was applied. There was no age-group by drug interaction (F(2,34)=0.490, p=0.539, η^2^_p_=0.014) there was a main effect of drug (F(2,34)=19.129, p<0.001, η^2^_p_,=0.360), but no main effect of age-group (F(1,34)=2.009, p=0.165, η^2^_p_=0.056). The main effect of drug was driven by higher ratings of coughing during ‘THC’ (t(34)=1.230, p=0.681, MD=0.158) and ‘THC+CBD’ (t(34)=4.748, p<0.001, MD=1.348) relative to ‘PLA’, and greater during ‘THC+CBD’ relative to ‘THC’ (t(34)=4.396, p<0.001, MD=1.19).

## 3.7 Exploring impact of inhalation time on THC plasma levels

Two MLMs were run to determine if inhalation time of second balloon explained any differences in THC plasma levels in ‘THC’ and ‘THC+CBD’. The addition of time to inhale second balloon as a fixed effect, time-varying predictor in Model 2 significantly improved model fit compared to Model 1: χ^2^_Change_=4.608, *df*_Change_=1, p<0.05. Moreover, in Model 2, time to inhale second balloon was a significant predictor of THC plasma levels at T2 (B =1.1405, SE=0.638, p=0.03). However, Drug (dummy variable encoding ‘THC’ and ‘THC+CBD’) and between person variance (intercept) are still highly significant (Table S21).

#### **Table S21.** Multilevel model results for THC plasma levels (T2)

|  |  | **Beta estimate** | | **S.E.** | **P value** | **-2LL** |
| --- | --- | --- | --- | --- | --- | --- |
| Model 1 | Intercept | 27.834 | 1.626 | | <0.001 | 699.934 |
|  | Drug | -12.950 | 1.719 | | <0.001 |  |
|  |  |  |  | |  |  |
| Model 2 | Intercept | 20.057 | 3.877 | | <0.001 | 695.326 |
|  | Drug | -11.946 | 1.698 | | <0.001 |  |
|  | Time to inhale 2nd balloon | 1.405 | 0.638 | | 0.03 |  |

Intercept: between-person variance; Drug: ‘THC+CBD’ set to zero as reference. S.E.=standard error; -2LL= -2 log-likelihood

# **4 Supplementary Discussion**

*CBD’s effects*

The presence of CBD appeared to increase ‘feel drug effect’, the PSI subscale cognitive disorganisation, and heart rate. It should be noted that the scientific literature concerning the ability of CBD to moderate the acute effects of THC is mixed and inconclusive (26). Typically, CBD appears not to modulate or temper THC’s acute effects (26). However, one vaporisation study reported CBD-induced augmentation of THC’s effects in infrequent but not frequent users, using 8mg of THC and 4mg of CBD (27).

*Pharmacokinetics of THC and CBD*

THC and CBD plasma concentrations vary greatly, between individuals and between studies, even when similar doses are given via the same route of administration. Furthermore, the impact of CBD on THC plasma levels is unclear. In Arkell et al (2019)(28), two vaporised cannabis conditions containing 13.75mg THC produced peak THC plasma concentrations of ~34ng/ml and ~42ng/ml. In Arkell et al (2020) (29), doses of 13.75mg THC produced peak THC plasma concentrations of ~20ng/ml. While in Van de Donk et al (2019)(30), 22.4mg vaporised THC produced a mean peak plasma THC concentration of 82ng/ml. These studies co-administered CBD with the following doses: 13.75mg CBD(28), 13.75mg CBD (29), and 17.8mg CBD and 18.4mg CBD (30), producing peak CBD plasma concentrations of ~60ng/ml (28), ~15ng/ml (29) and 80ng/ml and 155ng/ml (30).

It is theoretically plausible that CBD could pharmacokinetically augment plasma THC levels but simultaneously pharmacodynamically reduce the effect of THC (26,30) via allosteric modulation of the CB1 receptor (31). This could reconcile the greater plasma THC levels with similar subjective and behavioural effects, seen here and elsewhere (28). Alternatively, the presence of placebo cannabis somehow inhibited the vaporisation of THC in the Bedrocan cannabis (in the ‘THC’ condition). Our differences in THC plasma level were not driven by a small number of outliers; 93% (n=42) of the participants with available data (n=45) had greater plasma THC levels in ‘THC+CBD’ than in ‘THC’ condition.

Participants took significantly longer to complete the inhalation procedure during ‘THC+CBD’ compared to ‘THC’. This could conceivably impact THC plasma levels at T2, as blood samples were always drawn at +20 minutes after the start of inhalation. Previous studies involving smoking cannabis found that THC plasma levels reached their peak before the end of smoking (around 3 minutes after the start of inhalation) (32,33). Results from exploratory analyses testing this hypothesis revealed that inhalation time of the second balloon was a significant predictor of THC plasma levels. This makes sense given that those who took longer to inhale the balloon would have finished inhaling closer in time to the blood sample being drawn (T2, +20 minutes). However, when accounting for inhalation time, there was still a large and significant effect of drug. These results suggest inhalation time might account in part, but not entirely, for differences in THC plasma levels.

*Trend effects*

We also detected a trend interaction between age-group and drug for ‘anxious’, in which adults became more anxious following administration of active cannabis, but this was not seen in adolescents. Although this replicates the pattern of Mokrysz et al (2016) (1), it was not significant (p=0.062) and the effect size was half that of the previous study (our η^2^_p_=0.05, previous η^2^_p_=0.1). If we corrected for the number of VASs tested, this interaction would no longer be a trend.

*Key strengths*

Our study has numerous strengths. It is the first double-blind, placebo-controlled, randomised experiment to compare the acute effects of cannabis with and without cannabidiol in adolescents and adults. Our adolescent and adult cannabis users were carefully matched on cannabis use frequency, with similar means and distributions. Furthermore, most participants in both groups reported strong herbal cannabis to be their typical cannabis type, and they did not significantly differ on the estimated quantity of cannabis used on a day of use. Our adolescent and adult groups were generally well-matched, including on gender, socioeconomic status, ethnicity, problematic alcohol use, and frequency of illicit drug use. We biochemically verified drug abstinence using saliva tests and an alcohol breathalyser, which was corroborated by our pre-drug plasma results showing extremely low levels of THC and CBD at the start of each session. We measured plasma THC and CBD concentrations, again improving pharmacokinetic evidence on previous work (1,34). Our experiment was powered to detect an interaction between age-group and drug on PSI and we accounted for winner’s curse. We had 80% power to detect interactions on our outcome variables with an effect size of Cohen’s f≥0.24. Our experiment and primary outcome variables were registered on ClinicalTrials.gov and our analysis plan pre-registered on the Open Science Framework.

*Further limitations*

There were some group differences, namely adults had higher verbal intelligence, more frequent alcohol consumption, and less severe depression and anxiety symptoms, and lower impulsiveness than adolescents.

We recruited adults who began using cannabis at least weekly after age 18 to limit the impact of pre-18 cannabis use; however, adult cannabis users had a longer duration of use (since first trying cannabis) and had more lifetime days of cannabis use than adolescent cannabis users. It is theoretically possible that adolescent and adult cannabis users matched on lifetime cannabis exposure could show differential responses to cannabis, if long-term adult tolerance to cannabis counteracted natural adolescent tolerance. However, this hypothesis is not supported by existing work into tolerance to cannabis (35). In addition, it would be extremely difficult to recruit adolescent and adult cannabis users with matched lifetime cannabis histories.

# **References**

1. Mokrysz C, Freeman TP, Korkki S, Griffiths K, Curran HV. Are adolescents more vulnerable to the harmful effects of cannabis than adults? A placebo-controlled study in human males. Transl Psychiatry. 2016;6(11):e961–e961.

2. Morgan CJA, Freeman TP, Hindocha C, Schafer G, Gardner C, Curran HV. Individual and combined effects of acute delta-9-tetrahydrocannabinol and cannabidiol on psychotomimetic symptoms and memory function. Transl Psychiatry. 2018;8(1):1–10.

3. Lanz C, Mattsson J, Soydaner U, Brenneisen R. Medicinal cannabis: in vitro validation of vaporizers for the smoke-free inhalation of cannabis. PloS one. 2016;11(1):e0147286.

4. Hazekamp A, Ruhaak R, Zuurman L, van Gerven J, Verpoorte R. Evaluation of a vaporizing device (Volcano®) for the pulmonary administration of tetrahydrocannabinol. Journal of pharmaceutical sciences. 2006;95(6):1308–17.

5. Abrams DI, Vizoso HP, Shade SB, Jay C, Kelly ME, Benowitz NL. Vaporization as a smokeless cannabis delivery system: a pilot study. Clinical Pharmacology & Therapeutics. 2007;82(5):572–8.

6. Robinson SM, Sobell LC, Sobell MB, Leo GI. Reliability of the Timeline Followback for cocaine, cannabis, and cigarette use. Psychology of addictive behaviors. 2014;28(1):154.

7. Beck AT, Steer RA, Brown GK. Beck depression inventory. 1996;

8. Beck AT, Epstein N, Brown G, Steer RA. An inventory for measuring clinical anxiety: The Beck Anxiety Inventory. J Consult Clin Psychol. 1988;56(6):893–7.

9. Cyders MA, Littlefield AK, Coffey S, Karyadi KA. Examination of a short English version of the UPPS-P Impulsive Behavior Scale. Addictive behaviors. 2014;39(9):1372–6.

10. Holdnack HA. Wechsler test of adult reading: WTAR. San Antonio, TX: The Psychological Corporation. 2001;

11. Bohn MJ, Babor TF, Kranzler HR. The Alcohol Use Disorders Identification Test (AUDIT): validation of a screening instrument for use in medical settings. Journal of studies on alcohol. 1995;56(4):423–32.

12. Adamson SJ, Kay-Lambkin FJ, Baker AL, Lewin TJ, Thornton L, Kelly BJ, et al. An improved brief measure of cannabis misuse: the Cannabis Use Disorders Identification Test-Revised (CUDIT-R). Drug and alcohol dependence. 2010;110(1–2):137–43.

13. Sheehan D V, Lecrubier Y, Sheehan KH, Amorim P, Janavs J, Weiller E, et al. The Mini-International Neuropsychiatric Interview (MINI): the development and validation of a structured diagnostic psychiatric interview for DSM-IV and ICD-10. Journal of clinical psychiatry. 1998;59(20):22–33.

14. Mason OJ, Morgan CJM, Stefanovic A, Curran HV. The psychotomimetic states inventory (PSI): measuring psychotic-type experiences from ketamine and cannabis. Schizophr Res. 2008;103(1–3):138–42.

15. Baddeley A, Wilson BA. Prose recall and amnesia: Implications for the structure of working memory. Neuropsychologia. 2002;40(10):1737–43.

16. Kay SR, Fiszbein A, Opler LA. The positive and negative syndrome scale (PANSS) for schizophrenia. Schizophr Bull. 1987;13(2):261–76.

17. D’Souza DC, Perry E, MacDougall L, Ammerman Y, Cooper T, Wu Y te, et al. The psychotomimetic effects of intravenous delta-9-tetrahydrocannabinol in healthy individuals: implications for psychosis. Neuropsychopharmacology. 2004;29(8):1558–72.

18. Englund A, Morrison PD, Nottage J, Hague D, Kane F, Bonaccorso S, et al. Cannabidiol inhibits THC-elicited paranoid symptoms and hippocampal-dependent memory impairment. Journal of psychopharmacology. 2013;27(1):19–27.

19. Hawthorne G, Hawthorne G, Elliott P. Imputing cross-sectional missing data: comparison of common techniques. Australian & New Zealand Journal of Psychiatry. 2005;39(7):583–90.

20. Gmel G. Imputation of missing values in the case of a multiple item instrument measuring alcohol consumption. Statistics in medicine. 2001;20(15):2369–81.

21. Shrive FM, Stuart H, Quan H, Ghali WA. Dealing with missing data in a multi-question depression scale: a comparison of imputation methods. BMC medical research methodology. 2006;6(1):1–10.

22. Curran VH, Brignell C, Fletcher S, Middleton P, Henry J. Cognitive and subjective dose-response effects of acute oral Δ 9-tetrahydrocannabinol (THC) in infrequent cannabis users. Psychopharmacology (Berl). 2002;164(1):61–70.

23. Hindocha C, Freeman TP, Xia JX, Shaban NDC, Curran H V. Acute memory and psychotomimetic effects of cannabis and tobacco both ‘joint’and individually: a placebo-controlled trial. Psychological Medicine. 2017;47(15):2708–19.

24. Blanca Mena MJ, Alarcón Postigo R, Arnau Gras J, Bono Cabré R, Bendayan R. Non-normal data: Is ANOVA still a valid option? Psicothema, 2017, vol 29, num 4, p 552-557. 2017;

25. Schmider E, Ziegler M, Danay E, Beyer L, Bühner M. Is it really robust? Methodology. 2010;

26. Freeman AM, Petrilli K, Lees R, Hindocha C, Mokrysz C, Curran HV, et al. How does cannabidiol (CBD) influence the acute effects of delta-9-tetrahydrocannabinol (THC) in humans? A systematic review. Neuroscience & Biobehavioral Reviews. 2019;107:696–712.

27. Solowij N, Broyd S, Greenwood L marie, van Hell H, Martelozzo D, Rueb K, et al. A randomised controlled trial of vaporised Δ9-tetrahydrocannabinol and cannabidiol alone and in combination in frequent and infrequent cannabis users: acute intoxication effects. European archives of psychiatry and clinical neuroscience. 2019;269(1):17–35.

28. Arkell TR, Lintzeris N, Kevin RC, Ramaekers JG, Vandrey R, Irwin C, et al. Cannabidiol (CBD) content in vaporized cannabis does not prevent tetrahydrocannabinol (THC)-induced impairment of driving and cognition. Psychopharmacology (Berl). 2019;236(9):2713–24.

29. Arkell TR, Lintzeris N, Mills L, Suraev A, Arnold JC, McGregor IS. Driving-related behaviours, attitudes and perceptions among Australian medical cannabis users: results from the CAMS 18-19 survey. Accident Analysis & Prevention. 2020;148:105784.

30. van de Donk T, Niesters M, Kowal MA, Olofsen E, Dahan A, van Velzen M. An experimental randomized study on the analgesic effects of pharmaceutical-grade cannabis in chronic pain patients with fibromyalgia. Pain. 2019;160(4):860.

31. Hayakawa K, Mishima K, Hazekawa M, Sano K, Irie K, Orito K, et al. Cannabidiol potentiates pharmacological effects of Δ9-tetrahydrocannabinol via CB1 receptor-dependent mechanism. Brain research. 2008;1188:157–64.

32. Barnett G, Chiang CWN, Perez-Reyes M, Owens SM. Kinetic study of smoking marijuana. Journal of pharmacokinetics and biopharmaceutics. 1982;10(5):495–506.

33. Ohlsson A, Lindgren J, Wahlen A, Agurell S, Hollister LE, Gillespie HK. Plasma delta‐9‐tetrahydrocannabinol concentrations and clinical effects after oral and intravenous administration and smoking. Clinical Pharmacology & Therapeutics. 1980;28(3):409–16.

34. Murray CH, Huang Z, Lee R, de Wit H. Adolescents are more sensitive than adults to acute behavioral and cognitive effects of THC. Neuropsychopharmacology. 2022;1–8.

35. D’Souza DC, Cortes-Briones JA, Ranganathan M, Thurnauer H, Creatura G, Surti T, et al. Rapid changes in cannabinoid 1 receptor availability in cannabis-dependent male subjects after abstinence from cannabis. Biological psychiatry: cognitive neuroscience and neuroimaging. 2016;1(1):60–7.
